# Acknowledgment to Reviewers of *Journal of Clinical Medicine* in 2020

**DOI:** 10.3390/jcm10040865

**Published:** 2021-02-19

**Authors:** Journal of Clinical Medicine Editorial Office

**Affiliations:** MDPI AG, St. Alban-Anlage 66, 4052 Basel, Switzerland; jcm@mdpi.com

Peer review is the driving force of journal development, and reviewers are gatekeepers who ensure that *Journal of Clinical Medicine* maintains its standards for the high quality of its published papers. Thanks to the cooperation of our reviewers, in 2020, the median time to first decision was 17 days and the median time to publication was 36 days. The editors would like to express their sincere gratitude to the following reviewers for their precious time and dedication, regardless of whether the papers were finally published:
Aasa, UlrikaLieske, John C.Abate, AndreaLiesveld, JaneAbbara, AulaLietz-Kijak, DanutaAbbas-Aghababazadeh, FarnooshLièvre, AstridAbbate, Jessica L.Lighthall, Jessyka G.Abbina, SrinivasLildballe, Dorte LaunholtAbbott, DavidLillehei, KevinAbdelaziz, HusseinLillehoj, Erik P.Abderrahmani, AmarLilleri, DanieleAbdulmalik, OsheizaLillicrap, Thomas PatrickAbdulreda, Midhat H.Lim, Andy K. H.Abe, SusumuLim, Byung GunAbeles, ShiraLim, EdwinAberg, DavidLim, Hyun TaekAberra, Faten N.Lim, Jae-YolAbicht, AngelaLim, Jeong-HoonAbid, Karim AlexandreLim, JonathanAbraham, Michael G.Lim, Jun-SikAbramovitz, ItzhakLim, Mi YoungAbramson, Hanley N.Lim, SiewAbu Freha, NaimLim, Sung-HoonAbufaraj, MohammadLim, Wai H.Abuin, VanesaLiman, JanAbulseoud, Osama A.Limanowski, JakubAbutarboush, RaniaLimberg, Jacqueline K.Abuzaid, AhmedLimbucci, NicolaAccardi, GiuliaLimongelli, LuisaAccogli, EsteritaLin, AmyAchamra, NajateLin, Chih-HaoAchard, SophieLin, GuitingAchiron, AsafLin, Guo-HaoAcién, MaribelLin, Kuo-ShyanAcién, PedroLin, Michael M.Acosta, MonicaLin, ShengdaAcosta, StefanLin, WenActis, Giovanni C.Lin, Yen-KoAcuna-Villaorduna, AnaLin, Yi-TingAdachi, EisukeLin, Yu-HsuanAdam, ElisabethLinardon, JakeAdamczak, MarcinLindeman, JanAdamczewski, ZbigniewLindemann, StephanAdamczyk, PrzemysławLinden, SaraAdamek, AgnieszkaLindheimer, JacobAdamik, BarbaraLindqvist, Ebba K.Adamopoulos, PanagiotisLindqvist, PerAdams, LisaLindroth, HeidiAdams, SolomonLinetsky, ElinaAdamska, AgnieszkaLink, AlexanderAdamson, Christopher L.Link, Bjoern ChristianAdamson, Philip D.Linsenmeier, Robert A.Adan, AnaLinton, NatalieAdapa, SreedharLinz, ChristianAdegbola, Samuel O.Lio, AntonioAdekanmbi, VictorLio, Peter A.Adeleye, AmandaLionetti, VincenzoAdes, AlexLiopyris, KonstantinosAdey, DeborahLiou, Jr-JiunAdhikari, PrakashLiporace, FrankAdhikari, RajanLipšic, ErikAdonakis, GeorgeLipson, Michael J.Adornetto, AnnagraziaLipton, JeffreyAdriani, AlessandroLisi, LuciaAdriani, EzioLisi, SabrinaAeddula, Narothama ReddyLiskovykh, Mikhail A.Afelik, SolomonLisowska, BarbaraAfrashtehfar, KelvinLisowski, ZofiaAfrashtehfar, Kelvin IanLissa, DelphineAgarwal, Manyoo A.Lisy, KarolinaAgati, LucianoLitofsky, Norman ScottAgbaedeng, Thomas A.Litschauer, BrigitteAgerbæk, Mette Ø.Litscher, GerhardAggarwal, MeghaLittarru, GianpaoloAggarwal, SandeepLittle, BertAggarwal, SaurabhLittle, JaneAgil, AhmadLitvan, IreneAgoro, RafiouLitwin, TomaszÁgoston, CsillaLiu, AipingAgrafioti, AnastasiaLiu, AndrewAgrawal, AlokLiu, ChangAgrawal, PriyankaLiu, DeliAgrawal, VineetLiu, FengAgresta, FerdinandoLiu, JiaAguadé-Bruix, SantiagoLiu, LingAguglia, UmbertoLiu, NingAguilar-Company, JuanLiu, T. Y. AlvinAguilar-Diosdado, ManuelLiu, YanAguilera Correa, John JairoLiu, YonggangAguilera, MariLiu, YutaoAguirre, José A.LiWang, PatriciaAguirre-Urízar, José ManuelLizarraga, Karlo J.Agus, MarcoLjubimov, Alexander V.Ahamed, JasimuddinLlamas-Velasco, MarAhearne, MarkLlanes, CarlosAhern, DanielLlavero, FranciscoAhlman, Mark A.Llena, Carmen C.Ahluwalia, MeenakshiLleo De Nalda, AnaAhmad, AamirLleo, AnaAhmad, Baseer U.Llompart-Pou, Juan A.Ahmad, KhurshidLlorenç, VictorAhmad, ShakilLlorente Cantarero, Francisco JesusAhmadi, SaraLloyd, MeganAhmadzia, Homa K.Llueca, AntoniAhmed, Atif AliLo Faro, CarmeloAhn, Chang HoLo Muzio, LorenzoAhn, JeonghyunLo, Benjamin W. Y.Ahnert, PeterLo, Shih-YenAhrenfeldt, Linda JuelLobanova, Ekaterina S.Ahuja, SanchitLobdell, KevinAi, WaldenLocatelli, Anna Aiba, TakeshiLocatelli, DavideAibar-Almazán, AgustínLocatelli, FrancescoAiello, ValeriaLocatello, Luca GiovanniAigner, FelixLodi, RaffaeleAigner, ReneLoeffler, IvonneAimo, AlbertoLoewen, NilsAiraksinen, JuhaniLoffler, Kelly A.Aishima, ShinichiLofrumento, Dario DomenicoAizawa, ToruLogantha, SunilAizenberg, MicheleLogger, JadeAjaimy, MariaLoggini, AndreaAjduk, AnnaLöhr, MatthiasAjjan, RamziLoiudice, PasqualeAkagi, SatoshiLollobrigida, MarcoAkalin, AliLombardi, GiovanniAkalin, EnverLombardi, Vincent C.Akcalı, AliyeLombardo, GiorgioAkelina, YelenaLonardo, AmedeoAkhavan-Sigari, RezaLoncoman, CarlosAkhmetzhanov, Andrei R.Lonetti, AnnalisaAkhurst, Rosemary J.Long, AudreyAkiba, ChihiroLong, ElizabethAkiko, OshiroLongbrake, ErinAkiyama, HisashiLonghini, FedericoAkkari, LeilaLonghurst, HilaryÁkos Szabó, CharlesLongo, AntonioAkosile, WoleLongoni, SalvatoreAkouri, RandaLopes, LuísAkushevich, Igor V.Lopez Lopez, JoseAkwasi Atakora, AmoakoLópez Malo, DanielAlabraba, EdwardLópez, DanielAl-Ahmad, AbrahamLopez, HugoAlaimo, AlessandroLopez, JavierAlam, SaminaLopez, Jose IgnacioAlapati, Satish B.Lopez, MaribelAlawami, MohammedLopez-Campos, Jose LuisAlbaladejo Vicente, RomanaLopez-Cortes, LuisAlbani, DiegoLopez-Cruzan, MarisaAlbaugh, VanceLópez-Higes, RamónAlberio, LorenzoLópez-Medina, ClementinaAlbert, GrinshpunLópez-Miguel, AlbertoAlbert, SilviaLópez-Navarro, EmilioAlbert, StewartLópez-Parra, Ana M.Albert, UmbertoLópez-Pedrera, CharyAlberton, PaoloLopez-Picado, AmandaAlbertsen, Ida EhlersLópez-Pintor Muñoz, Rosa MaríaAlbertsmeier, MarkusLópez-Rodríguez, María L.Albini, AdrianaLopresti, PatriziaAlbo, ErikaLops, DiegoAlbrecht, Jennifer S.Lording, TimothyAlbrektsson, TomasLoregian, AriannaAlbro, Michael B.Lorentz, AxelAlcaraz, MiguelLorenz, KristinaAlcock, LisaLorenz, SusanneAlda, MartinLorenzo, MicheleAleksandrovych, VeronikaLorenzo-Gómez, Maria-FernandaAleksova, AnetaLoring, Ralph H.Aleksyniene, RamuneLorusso, RobertoAlessio, AnnovazziLosco, LuigiAlessio, HelaineLosi, Maria AngelaAlexiev, UlrikeLossnitzer, DirkAlexy, TamasLoster, Jolanta ElżbietaAlfano, GaetanoLosurdo, GiuseppeAlfarouk, Khalid OmerLottenberg, Lawrence L.Alfieri, SergioLoubet, PaulAlfieri, ValentinaLoucks, Laura AlexanderAlfonso, PicóLoughney, LisaAlgalarrondo, VincentLoughrey, DavidAlghetaa, Hasan F. K.Loukides, SteliosAlgood, HollyLourbopoulos, AthanasiosAli, FarwaLove, RobertAli, JuzarLovell, JonathanAlicic, RadicaLowe, AndrewAlijotas-Reig, JaumeLowe, GillianAl-Imam, AhmedLowe, JohnAljuboori, ZaidLowrie, LiaAlkhachroum, AyhamLowry, KristinAlkhateeb, AbedalrhmanLozupone, MadiaAlkhubaizi, QootLu, LingengAlkilany, ReemLu, PaulAl-kinani, Ali AthabLubas, ArkadiuszAllan, HarrietLubetzky, Anat V.Allaouchiche, BernardLubicky, John P.Allé, Mélissa C.Lubrano, ValterAllegaert, KarelLuby, MarieAllegra, EugeniaLuca, GianinaAllemann, FlorinLuca, GiovanniAllen, HerbertLucarelli, MarcoAllen, Lisa M.Lucas, JaneAllen, Robert J.Lucas, SebastianAllen-Brady, Kristina L.Lucas-Jiménez, OlaiaAllevi, FabianaLucchese, AlessandraAlmalla, MohammadLucia, AlejandroAlmansa, RaquelLuciani, PierfrancescoAlmeida, Gabriela M.Luckenbaugh, Amy N.Almeida, LuisLuddy, Kimberly A.Almeida, ShamikaLudwin, ArturAlmeida-Santos, Ana TeresaLufler, Rebecca S.Almekhlafi, Mohammed A.Lui, Roger YAlmerich-Silla, José ManuelLuigetti, MarcoAlnuaimat, HassanLukac, DavidAloisi, Anna MariaLukas, JanAlonso Salinas, Gonzalo LuisLukaszewska-Kuska, MagdalenaAlpay Savasan, ZeynepŁukaszewski, MarceliAl-Qawasmi, Riyad A.Lukin, Dana J.Al-Samkari, HannyLukkahatai, NadaAlsharedi, MohamedŁukomska, AgnieszkaAlt, VolkerLukomska-Szymanska, MonikaAltin, S. ElissaLum, Ying WeiAlTurki, AhmedLuminari, StefanoAlva, NormaLundh Hagelin, CarinaÁlvarez, DanielLundström, ClaesAlvarez, RogerLundström, LindaÁlvarez-Mercado, Ana IsabelLundstrom, TorbjornAlves, RicardoLundy, JoanneAlwahsh, SalamahLunenfeld, EitanAlzaid, FawazLunghi, MoniaAmano, TetsuyaLuo, Ji-DungAmano, ToshiyasuLupa, M. ConcettaAmarelli, CristianoLupattelli, AngelaAmaricai, ElenaLupberger, JoachimAmati, FrancescaLupi, Saturnino MarcoAmato, RosarioLuppi, FabrizioAmemiya, AyumiLupsor-Platon, MonicaAmerio, PaoloLurz, EberhardAmialchuk, AliaksandrŁuszczki, EdytaAmin, Minal M.Luthert, PhilipAmin, Nirav H.Lutkiewicz, KarolinaZylla, Maura M.Luzak, BoguslawaAmirav, IsraelLuzi, LivioAmirian, E. SusanLuzzago, StefanoAmital, HowardLyhne, Mads DamAmiya, EisukeLykoudis, Panagis M.Ammar, AchrafLynar, Sarah A.Ammirabile, AngelaLynch, KateAmmirati, EnricoLyons, KathleenAmoaku, Winfried M.Lyons-Weiler, JamesAmodio, PieroLyssek-Boroń, AnitaAmorim, AnitaLystad, Reidar P.Amorim-Costa, CéliaLytvynchuk, LyubomyrAmorim-de-Sousa, AnaMa, HongAmoroso, FrancescaMa, LiangsuoAmos, Christopher I.Ma, SunyoungAmundsen, Cindy L.Maalouf, WadihAmundsen, ToreMaass, Alexander H.An, Jeung HeeMaayah, ZaidAn, SeungMaccari, AlbertoAnand, KartikMacdonald, Heather R.Anapolski, MichaelMacDonald, IanAndabak Rogulj, AnaMacey, RichardAndereggen, LukasMachado-Aranda, DavidAndersen, Poul ErikMachen, Graham LukeAndersen, VidarMachin, DanielAnderson, AnnetteMachol, KerenAnderson, Greg M.Machoy, Monika ElżbietaAnderson, LarryMachuca-Portillo, GuillermoAnderson, LeslieMaciejczyk, MateuszAnderson, MichaelaMaciejewska, DominikaAnderson, RobertMacIntyre, NeilAndersson, Mattias K.Mackay, Emily J.Andò, GiuseppeMackey, DavidAndoniadou, CynthiaMaclachlan, LiamAndrea, CoppolaMacLeod, MeganAndreae, MichaelMacmillan-Crow, Lee AnnAndrew, JonesMaculewicz, EwelinaAndrews, Brian T.Madaan, SanjeevAndrews, JeanMadaline, TheresaAndrianello, StefanoMadariaga, AinhoaAndriessen, AnnekeMadaro, LucaAndriessen, KarlMaddalone, MarcelloAndroulakis, Ioannis P.Maddaloni, ErnestoAndrzej, NowickiMaddipati, VeerannaAnelli, LuisaMaddux, AlineAnesi, AlexandreMadeddu, CleliaAngelico, RobertaMadhukar, BurraAngelidi, AngelikiMadjid, MohammadAngelini, Dana E.Madka, VenkateshwarAngelone, TommasoMadkour, AichaAngelova, Plamena R.Madonna, RosalindaAngelovich, ThomasMadotto, FabianaAngelucci, EmanueleMadrigal, IreneAnichini, RobertoMaeda, KazuhiroAnifandis, GeorgeMaeder, Micha TobiasAnile, MarcoMaemoto, HitoshiAnis, EmanMafalda, SantosAnnesley, SarahMaffulli, NicolaAnnex, BrianMagal, MeirAnsa, BenjaminMagallón Neri, ErnestoAnsani, LuciaMagdanz, VeronikaAntal, MárkMager, Johannes J.Antelo, María LuisaMaggi, UmbertoAntignani, Pier LuigiMaggio, Maria GraziaAntinozzi, CristinaMaggioni, GiorgioAntipova, VeronicaMagiavini, LauraAnton, FernandMagit, AnthonyAntonarakis, Gregory S.Magli, AdrianoAntonelli Incalzi, RaffaeleMaglitto, FabioAntoni, AnnaMagliulo, GiuseppeAntoni, Michael H.Magni, ElenaAntoniak, SilvioMagni, PaoloAntonio, LeenMagnussen, ChristinaAntoniou, EvangeliaMagrassi, LorenzoAntonogeorgos, GeorgeMagri, FlaviaAntonucci, IvanaMagrina, JavierAntonuzzo, LorenzoMahabadi, Amir A.Antwi-Boasiako, CharlesMahata, SushilAnyfantakis, Dimitrios I.Maheswari, AkhilAnzivino, RobertaMahjani, BehrangAoki, MakotoMähler, AnjaAoki, MasayoMahli, AbdoAoki-Nonaka, YukariMahmud, NadimAonuma, KazutakaMahtani, ReshmaAouad, PhillipMai, Hang NgaAoyama, NaokiMaiello, MarcoAoyama, NorioMailhot, GenevieveAparicio, JorgeMaillard, JulienAparicio, Jose RamonMainra, RahulAparicio-Ugarriza, RaquelMains, Richard E.Apra, CarolineMaione, Angela SerenaAqrawi, LaraMaiorano, EugenioAquavella, James V.Maisano, DomenicoAquino-Martinez, RubenMaisch, BernhardArad, MichaelMaisel, KatharinaAragao, IreneMaiti, GeorgeAragona, OrianaMaitland-van Der Zee, Anke H.Arai, ChihiroMaitra, RadhashreeArai, KunizoMaj, MalgorzataArai, MasamiMajeed, HaroonArakaki, TatsuyaMajem, BlancaAraki, TetsuroMajewski, SebastianAranda-García, SilviaMaji, DebnathArase, YoshitakaMajmundar, MonilArba, FrancescoMajorana, AlessandraArbini, ArnaldoMajumder, RinkuArcidiacono, Paolo GiorgioMakaryus, RanyArcurio, Lindsay R.Maki, TakakuniArdakani, Nima MesbahMakin, StephenArdizzone, SandroMakkia, RashaArefian, HabibollahMakrygiannakis, Miltiadis A.Arena, ClaudiaMakvandi, PooyanArends, IrisMalachowska, BeataArgentiero, AntonellaMalagu', MicheleAriani, AlaricoMalapelle, UmbertoArias-Cabrales, Carlos E.Malaspina, DoloresArici, CeciliaMalatino, LorenzoArima, YuichiroMalebranche, MaryAris, Izzuddin M.Malejczyk, JacekArita, ReikoMalek, AlexandreAriza, Maria EugeniaMałek, ŁukaszArmario, PedroMaletsky, LorinArmeni, TatianaMaletzki, ClaudiaArmstead, William M.Malfitano, Anna MariaArnaboldi, PaulMałgorzewicz, SylwiaArnaud, ClaireMalik, Rayaz A.Arnbjörnsson, EinarMalik, TaimurArnhold, JürgenMalinowska, Anna M.Arnold, RudolfMalins, SamuelArnoldner, ChristophMalkiewicz, BartoszArnoult, DamienMallamaci, FrancescaAroke, EdwinMallela, Shamroop KumarArora, GunjanMallet, JasminaArora, SohrabMalli, FoteiniArponen, HeidiMallmann, Michael RudolfArps, KellyMallows, AdrianArranz, Laura-IsabelMaloberti, AlessandroArrich, JasminMalosio, Maria LuisaArroyave, Nora VanegasMalo-Urriés, MiguelArruza, LuisMalpeli, GiorgioArshad, HassanMaltais, MathieuArteel, Gavin E.Malterud, KirstiArthur, HelenMaltesen, RalucaArthurs, BryanMałysz - Cymborska, IzabelaArtigas, AntonioMamas, Mamas A.Arunamata, AlisaMamelund, Svenn-ErikArvanitaki, MariannaMammadova-Bach, ElminaArvelakis, AntoniosMamouni, KenzaArweiler-Harbeck, DianaMañas, AsierAsa’ad, FarahMancinelli, RominaAsaad, WaelMancini, AnnamariaAsadipooya, KamyarMancini, CeciliaAsaeda, MakotoMancini, FrancescaAsahina, IzumiMancini, ManueleAsahina, KinjiMancini, ValentinaAsano, NaokiManco, LicínioAsano, YoshihideMancuso, AndreaAsatryan, BabkenMancuso, Maria ElisaAscoli Marchetti, AndreaMandal, AmritlalAseervatham, JayaManenti, AntonioAshimatey, Bright SenyoManfredini, FabioAshktorab, HassanManfredini, RobertoAshrafian Bonab, MaziarMang, Cameron S.Ashton, JohnMangla, AnkitAsif, MohammedMangoni, Arduino A.Aslanian, HarryMani, AlirezaAslanides, Ioannis M.Manicone, Paolo FrancescoAslanidis, TheodorosManios, AndreasAsleh, RabeaMankia, Kulveer S.Aso, EsterMankowska-Wierzbicka, DorotaAssadsangabi, RezaMann, MichaelAsselin-Labat, Marie-LiesseMann, StewartAssenza, GiovanniMannelli, LorenzoAszmann, Oskar C.Mannello, FerdinandoAta-Ali, JavierManni, GianlucaAtchison, David A.Männikkö, NikoAthanasia, PrintzaManninger, MartinAthinarayanan, ShaminieMannino, DavidAthyros, VasiliosMannino, David MAtkin, Stephen L.Manolopoulos, Vangelis G.Atkinson, AndrewManor, IrisAtkinson, Henry Dushan E.Mansson, ChristopherAtrash, ShebliMansuri, ShahidAtsuta, YoshikoManta, RaffaeleAttali, BernardMantilla Herrera, Ana MariaAtwal, Paldeep S.Manto, MarioAtzler, DorotheeMantripragada, Venkata P.Au, LewisManuel Giorgi, FedericoAudi, SaidManuelli, MaurizioAuer, Rebecca C.Manzo, CiroAugello, MarcelloMapelli, MassimoAugusto, Jean-FrançoisMaple, PeterAurilio, GaetanoMar Rus, CalafellAuvinen, AnssiMarabese, MirkoAuzmendi-Iriarte, JaioneMarambaud, PhilippeAvanzas, PabloMaranchie, JodiAvelino, AntonioMarano, MassimoAvery, Ryan J.Marasca, ClaudioAvian, AlexanderMarascio, NadiaAvitabile, TeresioMarasco, GiovanniAvtanski, DimiterMaraví-Poma, EnriqueAwad, MilenaMarc, LaineAwada, HassanMarchesi, FedericoAy, CihanMarchi, GiacomoAyache, SamarMarchioni, MicheleAyerbe, LuisMarchuk, Douglas A.Aymami, MarieMarciello, MarziaAyoub, AshrafMarco, ArdigoAysegül, Ilhan-MutluMarco, SchiavoneAyuse, TakaoMarconi, GiovanniAyyadurai, PuvanalingamMarcos, LopezAyyathurai, RajinikanthMarcos-Pérez, DiegoAzad, Nilofer SabaMarculescu, RodrigAzaïs, HenriMarcusson, JanAzarnia Tehran, DomenicoMarcuzzo, StefaniaAzem, FoadMardin, Christian Y.Azer, Samy A.Marenzi, GiancarloAziz, FahadMareschi, KatiaAzodi, MasoudMaresh, MichaelAzrad, MariaMargalit, IliAzrad, MayaMargulis, Andrea V.Azuma, Mariane MaffeiMaria Irene, BelliniAzuma, NobuyoshiMaria Nuzzo, AnnaAzziz, RicardoMaria, Giner-SorianoB. Mariottoni, EduardoMarie, Jean-PaulBabajko, SylvieMarignol, LaureBabapoor-Farrokhran, SavalanMarín, MercedesBabel, PrzemyslawMarinelli, LucaBabst, RetoMarini, SandroBaccarani, UmbertoMarino, Marco VitoBacci, ChristianMarinò, MicheleBaccouche, AsmaMarino, Paolo NicolaBacher, UlrikeMario, AlovisiBacherini, DanielaMario, MiniatiBach-Gansmo, ToreMarion, Marie-HeleneBachler, MirjamMariotti, CesareBachmann, FriederikeMariscalco, GiovanniBäcker, Henrik C.Marjanski, TomaszBacker, JantienMark, Tomer M.Badau, DanaMarker, Ryan J.Badhiwala, JetanMarks, RayBadiali, GiovanniMarkuszewski, MichałBadour, Christal L.Markvart, MereteBadros, AshrafMarley, Julia V.Bae, JeehyeonMarlicz, WojciechBaek, Kyung InMarmor, Meir TibiBaek, SehyunMarongiu, GiuseppeBaek, Seung-HakMaroon, JosephBaert, ThaisMarote, AnaBaertling, FabianMarques, DuarteBaeyens, Jean-PierreMarques, MarisaBaeyens, NicolasMarques, PedroBaezconde-Garbanati, LourdesMarques, SusanaBagavant, HariniMarrazzo, PasqualeBagetta, GiacintoMarrocco-Trischitta, Massimiliano M.Baghdadi, Ziad D.Marrone, OresteBagheri, ZahraMarrow, LynneBagiu, Iulia-CristinaMarsch, AmandaBagnato, GianlucaMarsch, StephanBagot, Catherine N.Marsh, Courtney A.Bagrov, AlexeiMarshall, ChristopherBagüés, AnaMarshall, SkyeBahmad, HishamMarsili, StefaniaBahra, MarcusMarson, PieroBaht, GurpreetMartens, GerardBai, MingfengMartín Valero, RocíoBaile Ayensa, José IgnacioMartin, Allan R.Bailey, PippaMartin, DanielBailly, SébastienMartin, FranciscoBain, SteveMartin, Luc J.Baine, Michael J.Martin, LudovicBaj, JacekMartina, StefanoBajaj, JeevishaMartinelli, GiovanniBajaj, PuneetMartinez Santos, AnaBajaj, SahilMartínez, IsidoroBajic, PetarMartinez, Luis R.Bajo Lorenzana, Victoria MMartinez, Stephanie E.Bakht, MartinMartinez, UrsulaBakris, George L.Martínez-Åguila, AlejandroBakulski, Kelly M.Martínez-Arnau, Francisco MiguelBalani, JyotiMartínez-Hackert, ErikBalaphas, AlexandreMartinez-Jarreta, BegoñaBalashova, NataliyaMartínez-Loredo, VíctorBalasubramani, Goundappa K.Martínez-Martínez, IreneBalasubramanian, PriyaMartinez-Saguer, InmaculadaBalbi, BrunoMartinez-Useros, JavierBaldacci, FilippoMartinez-Vispo, CarmelaBaldassarre, Maria ElisabettaMartin-Galiano, Antonio J.Baldereschi, MarziaMartini, AlbertoBaldetti, LucaMartín-Montalvo, AlejandroBaldi, IleanaMartino Alba, RicardoBaldinger-Melich, PiaMartino, AlessandroBaldini, NicolaMartinod, KimberlyBalducci, ClaudiaMartins, Maria Do RosárioBalédent, OlivierMartins, NatáliaBalica, Adrian CMartins, Sandra F.Balistreri, Carmela RitaMartín-Sánchez, Francisco JavierBalkau, BeverleyMartire, BaldassarreBallard, ZacharyMartirosian, GayaneBallmann, ChristopherMartone, Anna Maria.Ballweg, Jean A.Martucci, GennaroBalmer, LoisMartynowicz, HelenaBalne, Praveen KumarMaruhashi, TatsuyaBalogh, Esther A.Maruya, KoheiBalogh, ZsoltMaruyama, TesshoBaloira Villar, AdolfoMaruyama, YukioBaloni, PriyankaMary, DidierBalzano, TizianoMarzano, Angelo ValerioBalzano-Nogueira, LeandroMarzi, IngoBalzarro, MatteoMarzilli, MarioBambling, MatthewMarzuillo, PierluigiBamforth, SimonMasarone, DanieleBanach, MaciejMascagni, DomenicoBandapalli, Obul ReddyMascitti, MarcoBandmann, OliverMaselli, Diego J.Banerjee, AnweshaMaselli, FilippoBanerjee, GouravMasiak, JolantaBang, Chang SeokMasic, UnaBangash, MansoorMasior, ŁukaszBanitsas, Konstantinos A.Maslin, KateBankhead, ArmandMasmoudi, HichamBanks, LoriMason, JayBansal, AmitaMason, LauraBaptista, FátimaMaspero, CinziaBär, SarahMassari, FrancescoBarabas, PeterMassaro, MarikaBaragetti, AndreaMassaro, SebastianoBarak, BoazMassé-Alarie, HugoBarak, MiraMassin, PhilippeBarak, SharonMassó, PalomaBaran, BengiMast, T. DouglasBaran, WojciechMast, ThomasBaranchuk, AdrianMasterson, Timothy A.Barassi, GiovanniMasthoff, MaxBaratto, LuciaMastoraki, AikateriniBarault, LudovicMastroianni, RiccardoBarauskas, GiedriusMastropietro, AlfonsoBarb, Adam WMastrotto, FrancescaBarbalho, Sandra MariaMasturzo, BiancaBarbanti, MarcoMasuda, FumiBarbaresko, JanettMasumoto, HidetoshiBarbaro, NatáliaMasutani, KosukeBarbas, Andrew S.Mata, ManuelBarbe, Mary F.Mataix-Cols, DavidBarber, DomingoMatalliotakis, MichailBarbero, CristinaMatarazzo, Maria GraziaBarbhaiya, Chirag R.Matarese, GiovanniBarbone, AlessandroMatet, AlexandreBarbonetti, ArcangeloMathai, Susan K.Barbosa-Breda, JoaoMathes, Alexander M.Barcellona, DorisMatheson, BrittanyBarcz, EwaMathevet, PatriceBardaji, AlfredoMathew, MonaBardin, RonMathieson, StephanieBarisano, GiuseppeMathys, VanessaBarlier, AnneMatiakis, ApostolosBarnas, EdytaMatin, AbdulBarnea, Eytan R.Mato, EugeniaBarnes, MareeMatrone, AntonioBarone, RitaMatsubara, KeikoBaron-Stefaniak, JoannaMatsubara, ShigekiBar-Or, DavidMatsuda, AkiraBarracano, RosariaMatsuda, HiroshiBarralet, JakeMatsuda, NaoyukiBarrasa, HelenaMatsuda, ShinjiBarratt, JonathanMatsuda, TakashiBarratt, ShaneyMatsugi, AkiyoshiBarre, EdwardMatsukawa, YoshihisaBarrea, LuigiMatsumoto, HiromiBarreiro, EstherMatsumoto, KenjiBarreto, Jason N.Matsumoto, KunihitoBarrett, Andrew WilliamMatsumoto, MasanoriBarrio, JesúsMatsumoto, ShokeiBarrios-Rodríguez, RocioMatsumoto, ToshiharuBarroso-García, VerónicaMatsunaga, YasukaBartáková, VendulaMatsuo, KojiBartel, SabineMatsuoka, TeruyukiBarth, Rolf N.Matsusaki, TakashiBartlett, HannahMatsushita, KensukeBartunek, JozefMatsuzaki, JuntaroBarutcu, RasimMatsuzaki, ShinyaBarwick, AlexMatta, RagaiBaryshnikova, EkaterinaMattace-Raso, Francesco U. S.Barzi, AfsanehMatte, AlessandroBasak, IndranilMatthew, BeneschBasecke, JorgMatthews, EvanBasiri, RaedehMatthews, Ray VernBasit, AbdulMatthiesen, RuneBasnayake, ChamaraMattina, AlessandroBassand, Jean-PierreMattner, JochenBassareo, Pier PaoloMattogno, Pier PaoloBassetti, StefanoMattsson, ErneyBassetto, FrancoMattsson, JanetBassi, Anna MariaMatucci Cerinic, MarcoBassir, HosseinMatusik, PawelBastarrachea, Raul A.Matusik, PawełBaster, ZbigniewMatute-Bello, GustavoBastola, PrabhakarMauceri, RodolfoBasu, RupsaMaugeri, GraziaBasu, SayanMaulucci, Christopher M.Batailler, CécileMaura, ArosioBateman, BronwynMaurea, NicolaBateman, Ryon MMaurer, BarbaraBatista, MilanMauri, GianlucaBatko, BogdanMauriello, AlessandroBatool, MariaMaurin, MaxBatra, ManavMauro, Adolfo GabrieleBattaglia, YuriMauro, LoredanaBattaglini, DeniseMavilio, DomenicoBattista, MarcoMavragani, AmaryllisBattista, Valeria Marinella AugustaMawhinney, ThomasBatty, Kevin T.May, Christian AlbrechtBaturova, Maria A.Mayberry, JohnBauckneht, MatteoMayer, Deborah K.Bauer, Christopher E.Mayer-Pickel, KarolineBauer, Philippe R.Maylor, ElizabethBaumeister, DavidMaynard, OliviaBaumgartner, JosefMay-Newman, KarenBavle, AbhishekMazidi, MohsenBawadekar, MandarMazur, Marcus D.Baxter, EvaMazur, MartaBaydur, AhmetMazzola, MicheleBayoumi, AliMazzone, ElioBazrafshan, ZahraMazzone, PatrizioBeaudart, CharlotteMazzotta, CosimoBeaumont, MarcMbagwu, Smart IkechukwuBeaune, SébastienMcAdams, CarrieBeavis, Kathleen G.McAllister, James PattersonBecher, Peter MoritzMcBride, KateBechis, Seth K.McBride, OrlaBechsgaard, ThorMcCall, Catherine A.Becker, Allan B.McCall, Maureen A.Becker, StephenMcCann, Matthew R.Beckermann, Kathryn EbyMcCaughan, Geoffrey W.Bédard, AnnabelleMcCaughan, Geoffrey WilliamBednařík, JosefMcClain, Craig D.Beer, Ambros J.McClure, Philip K.Beetz, OliverMcCluskey, StuartBegemann, Marieke J. H.McComb, JacalynBegemann, MartinMcCommis, Kyle S.Begin, PhilippeMcCord, Joe M.Begnoni, GiacomoMcCormick, ThomasBehar, AlbertoMcCrudden, MaeliosaBehbehani, SadikahMcCully, JamesBehem, Christoph R.McCutcheon, KeirBehr, BjornMcDermott, MollieBehrmann, LenaMcDermott-Roe, ChrisBelayew, AlexandraMcDonald, ChristineBelcarz, AnnaMcDonald, Emily G.Belik, JaquesMcDonnell, AlisonBell, DianaMcDowell, MichaelBellanti, FrancescoMcFadden, SandraBellary, SrikanthMcFadyen, JamesBellasi, AntonioMcgee, David J.Bellavia, DanieleMcGilvray, KirkBellin, MilenaMcGregor, NeilBellini, Maria IreneMcGregor, Stephanie M.Bellini, MassimoMcGuire, Joseph F.Bellini, PierantonioMcIlroy, MarieBello, SalvadorMcIntosh, KennethBellosta, StefanoMcIntosh, Virginia V. W.Bellot-Arcís, CarlosMcKay, DianeBelluzzi, ElisaMcKay, TaraBelmin, JoëlMcKay, TinaBeltran-Alvarez, PedroMcKay, William P.Belzunce, Martín AlbertoMcKelvie, Penny A.Bemeur, ChantalMcKenna, Malachi J.Ben Hammouda, ManelMcKenna, Peter EdwardBenaglia, LauraMcLay, LaurieBen-Ami-Shor, DanaMcLennan, PeterBenca, EmirMcLeod, Niall M. H.Bence M., NagyMcLucas, BruceBen-Eliyahu, ShamgarMcMahon, DerekBenfaremo, DevisMcMahon, FrancisBen-Haroush, AviMcMullin, Mary FrancesBenini, StefaniaMcNally, Martin A.Bennardo, LuigiMcnutt, PatrickBennett, Robert G.McPherson, Nicole O.Bennis, YoussefMcSorley, HenryBenson, SarahMcVey, David ScottBenzeroual, Kenza E.McVicker, BenitaBen-Zvi, DannyMeadows, StryderBeom, JaewonMeans, Robert T.Berardi, Anna ConcettaMeccariello, LuigiBerardi, RossanaMedeiros-Domingo, ArgeliaBerbrayer, DavidMedina, Reinhold J.Berentsen, SigbjornMedina-Inojosa, JoseBerg, Britt-IsabelleMedjeral-Thomas, NicholasBerg, PhilippMedrano, F. J.Bergandi, LoredanaMędrzycka, WiolettaBergantini, LauraMeduri, AlessandroBergdahl, AndreasMedvedev, AlexanderBerger, Adam C.Meek, Patrick D.Berger, Christoph T.Megaloikonomos, Panayiotis D.Berger, FabianMegna, MatteoBerger, SlavaMehilli, JulindaBerger-Groch, JosephineMehl, Christian JohannesBergholz, RobertMehrabi, ArianebBergler, TobiasMehta, Ambereen KurwaBergmann, TroelsMehta, BellaBergmeijer, ThomasMehta, DhruvBergstra, Sytske AnneMehta, GeetaBeri, KavitaMehta, HirenBermo, Mohammed S.Mehta, NabilBernabei, FedericoMehta, NoshirBernabéu, CarmeloMehta, SanjayBernabeu-Wittel, MáximoMehta, Shameer J.Bernard, DidemMehta, ShwetalBernardes, RafaelMeier, JensBernardi, SimonaMeier, JilBernardino, Raquel L.Meier, KimberlyBernardo, CarinaMeier, Raphäel P. H.Bernard-Valnet, RaphaelMeier, UllrichBernaudin, FrancoiseMeier-Gibbons, FrancesBernaudin, Jean FrançoisMeier-Stephenson, VanessaBernstein, Elana J.Meinel, ThomasBerntorp, ErikMeinhold-Heerlein, IvoBernts, Lucas H. P.Meinitzer, AndreasBeropoulis, EfthymiosMeisel, Hans JoergBerrington, JanetMeisel, Robert L.Berry, NeilMeister, Melanie R.Berryman, CarolynMei-Zahav, MeirBershteyn, AnnaMejersjö, ChristinaBertaina, MaurizioMekhemar, MohamedBertani, LorenzoMekraksakit, PoemlarpBerteau, Jean-PhilippeMelcher, MarcBerth, HendrikMelegari, GabrieleBerthelot, LaurelineMelexopoulou, Christina A.Berthier, CelineMeli, VijaykumarBerti, AlviseMelilli, EdoardoBertin, Francois-ReneMelina, GiovanniBertol, ElisabettaMelis, MelaniaBertolaccini, LucaMelly, LudovicBertolazzi, ChiaraMelnick, GlennBertoldo, FrancescoMelo, PauloBertolino, PhilippeMeloni, MarcoBertsias, GeorgeMelsens, KarinBesnier, FlorentMena-Álvarez, JesúsBessaud, MaëlMenahem, SolomonBesse, LenkaMendall, MichaelBesser, MartinMende, MeinhardBeswick, Ellen J.Mendelowitz, DavidBetsch, MarcelMéndez-Barbero, NereaBettencourt-Silva, RitaMendez-Lagares, GemaBeukes, EldreMendonça, MarceloBeuriat, Pierre-AurélienMenè, PaoloBeversdorf, DavidMeneguzzo, PaoloBezabhe, Woldesellassie M.Menendez, AlfredoBEZU, LucilliaMenéndez, RosarioBhan, IrunMenezo, YvesBhanji, Rahima A.Meng, Qing-heBhardwaj, BhaskarMengel, DavidBhatawadekar, Swati A.Mengozzi, ManuelaBhatia, DivyaMenin, DamianoBhatia, RakeshMenini, MariaBhattacharjee, SandipanMenkhorst, EllenBhattacharya, AnannyaMenon, RejeeshBhattarai, YogeshMenon, RijuBhatti, DanishMenozzi Jäckli, MarinoBhavadharini, BalajiMentis, Alexios-Fotios A.Bhave, DevayaniMenzaghi, ClaudiaBhavsar, Mit B.Meraviglia, VivianaBhutani, Manoop S.Mercadante, SebastianoBhute, ShrikantMercado, FranciscoBiagetti, BetinaMerchán-Baeza, Jose AntonioBiagioni, AlessioMercury, Santo RaffaeleBiancardi, VivianMergoni, GiovanniBiancari, FaustoMerico, ValeriaBianchetti, Mario G.Merigliano, StefanoBianchi, AlbertoMerino, BeatrizBianchi, JonasMerino, Jaime M.Bianchini, FrancescaMerla, CristinaBianco, FrancescoMerlino, GiovanniBianco, Maria RitaMerlo, MarcoBianconi, VanessaMerlotti, DanielaBiasin, MaraMerola, ElettraBibb, RichardMertz, Kirsten D.Bicalho, Rodrigo CarvalhoMerviel, Philippe.Biebl, MatthiasMerz, BenediktBiecker, ErwinMerz, C. Noel BaireyBiegon, AnatMerzon, EugeneBiehl, ChristophMeshram, ChetanBielinska-Waz, DorotaMesquita, JoanaBielsa, SilviaMessenio, DarioBień, AgnieszkaMesserli, Shanta M.Biener, MoritzMessias, AnaBierle, CraigMessiha, HadiBiernacka, Elżbieta Katarzyna.Messineo, LudovicoBiglia, NicolettaMessini, Christina I.Bijak, MichałMessmer, Anna S.Bijnsdorp, IreneMestre, DanielBikas, AthanasiosMestre, TiagoBikov, AndrásMestre-Pinto, Joan IgnasiBikson, MaromMestres, CarlosBilancio, AntonioMeszaros, MagdalenaBilińska, Zofia T.Metere, AlessioBillaud, MarieMethenitis, SpyridonBillia, FilioMetovic, JasnaBilski, JanMettler, LiselotteBilsky, Mark HMetyas, SamyBinda, CeciliaMetzl, MarkBingham, CoralieMeyer, JesseBingisser, RolandMeyer-Ficca, Mirella L.Binkley, ElaineMeyer-Lueckel, HendrikBiondi, AntonioMeylan, SylvainBird, Thomas G.Meyssonnier, VaninaBirdir, CahitMezi, SilviaBirjandi, Anahid AhmadiMiano, MaurizioBirlea, MariusMiao, JiBirnbaum, YochaiMiazgowski, TomaszBirrell, MarkMicali, GiuseppeBiscetti, FedericoMichael, MarmuraBishop, LauriMichael, MichaelBisson, CatherineMichaelidou, Alexandra-MariaBiviá-Roig, GemmaMichal, MatthiasBIVONA, GiuliaMichalak, EwaBixel, KristinMichálek, PavelBizzoca, DavideMichaliszyn, Sara FleetBjelland, SveinMichalopoulos, EfstathiosBjorkavoll-Bergseth, MagnusMichalski, AndrzejBjørndal, LarsMichaud, LangisBjursten, HenrikMichel, LarsBlack, Beth PerryMichel, OlafBlack, Dennis D.Middleton, Peter G.Blackard, JasonMiedema, Jelle R.Blackburn, JessicaMieke, HulensBlackshaw, AshleyMiere, AlexandraBlackstone, CraigMierzwiński, JózefBlackstone, SarahMierzyński, RadzisławBlagev, Denitza P.Migdalis, IliasBlair, John E. A.Migita, OhsukeBlakely, Andrew M.Migliaccio, Anna RitaBlanchard, Thomas G.Migliorati, MarcoBlanco, Juan F.Migliore, FedericoBlanco-Donoso, Luis ManuelMignano, AntoninoBlanker, MarcoMihaicuta, StefanBlanker, Marco H.Mihaila, IuliaBlanton, Robert M.Mihailovic, NatasaBlaser, MarkMihala, GaborBleeck, StefanMihalca, Andrei DanielBleilevens, ChristianMihashi, ToshifumiBlesson, Chellakkan SelvanesanMikami, MikioBloemberg, GuidoMikhail, MikelBloemers, JosMiki, AkikoBloksgaard, MariaMiki, KojiroBlomgren, KarinMiki, YasuhiroBlomme, EricMikołajczyk-Chmiela, MagdalenaBloor, IanMikulis, David J.Blum, ArnonMilan, AlbertoBlümel, BenjaminMilanetto, Anna CaterinaBlumenfeld Olivares, Javier AndresMilani, Gregorio P.Boaz, KarenMilani, Gregorio PaoloBobiński, MarcinMilano, AnnalisaBoccardi, VirginiaMilanowski, LukaszBoccatonda, AndreaMilazzo, MarioBocchieri, SalvatoreMilenkovic, AndreaBocci, TommasoMiletta, MariaBocedi, AlessioMilewska, AleksandraBochicchio, BrigidaMiliauskas, SkaidriusBock, JonathanMillan-Cayetano, Jose-FranciscoBodi, VicenteMillar, Audrey LynnBodner-Adler, BarbaraMillar, J. CameronBoduszek, DanielMillecamps, MagaliBody, SimonMiller, Anthony B.Boe, Brian A.Miller, Eliza CBoeldt, Derek SMiller, Jillian VinallBoerma, MarjanMiller, John B.Boffano, MicheleMiller, Rachel G.Boffano, PaoloMiller, Virginia MBogani, GiorgioMills, Devin J.Bogdanos, Dimitrios P.Mills, Jesse N.Bogdański, PawełMills, KenBoggi, UgoMilne, StephenBogomolovas, JulijusMilosevic, MichaelBogusiewicz, MichałMin, Jeong JinBogusławska, JoannaMin, Jung-joonBohlmann, MichaelMin, Kyung-SanBöhm, RuwenMin, ThinzarBoilève, AliceMina, RobertoBoinska, JoannaMinakata, YoshiakiBoissier, FlorenceMinami, ShujiroBojar, IwonaMinami, TakashiBojarska, JoannaMinamiguchi, HitoshiBoland, BenoîtMiñana, GemaBolaris, MichaelMinardi, CarmeloBoletis, IoannisMineharu, YoheiBollag, WendyMinervini, GiuseppeBollinger, Kathryn ElizabethMinhas, Paras SinghBoltze, JohannesMinhas, SuksBömer, NilsMinian, NadiaBompolaki, DespoinaMinistrini, StefanoBonacina, FabriziaMinna, EmanuelaBonanad, ClaraMinnelli, CristinaBonanad, SantiagoMinniti, AntoninaBonanno, SilviaMinotti, BrunoBonato, MatteoMintz, Gary S.Bonaventura, AldoMinuz, PietroBonavia, AnthonyMiotla, PawelBonay, MarcelMiotto, KarenBonder, AlanMiozzo, MonicaBonderman, DianaMir, MonicaBonello, FrancescaMirabelli, MariaBonetti, GraziellaMiranda Jr., RobertBonfrate, LeonildeMiranda, João M.Bongiovanni, AlbertoMirbeik-Sabzevari, AmirBongiovanni, DarioMirijello, AntonioBongiovanni, LauraMirmohammadi, Hesam H.Bonichini, SabrinaMiron, NicolaeBonifacio, AloisMiron, Richard J.Bonifacio, Maria AddolorataMirsaeidi, MehdiBonifacio, MassimilianoMirshahi, MassoudBonifazi, MartinaMiserocchi, GiuseppeBonnemains, LaurentMishina, MasahiroBonomi, MarcoMishra, AbheepsaBonsignore, Maria RosariaMishra, AlaknandaBooij, LindaMishra, Jay S.Boomars, KarinMishra, ManishBoone, Brian A.Mishra, MeerambikaBoota, MariamMiskiewicz, PiotrBooth, Kevin T.Miśkiewicz, PiotrBorbély, YvesMisra, RanjitaBordon, JoseMistiaen, WilhelmBorg, Danielle J.Mistraletti, GiovanniBorghesi, AndreaMita, KojiBorghesi, SimonaMitacchione, GianfrancoBorghini, AndreaMitani, SeiichiroBorgnakke, Wenche S.Mitchell A, SullivanBorja, Pedro MolinerMitchell, AnnekaBorjini, NozhaMitchell, JenniferBorkowska, Edyta MartaMitchison, Hannah M.Bornstein, EranMitnitski, ArnoldBorody, ThomasMitola, StefaniaBorovac, Josip A.Mitra, Amal K.Borras, TeresaMitra, JoyBorrelli, Eric P.Mitrakas, LamprosBorrelli, JosephMitrofanova, AllaBorroni, DavideMitsch, ChristophBorsani, OscarMitsiadis, Thimios A.Bortkiewicz, AlicjaMitsunari, KensukeBortnick, AnnaMittlmeier, Thomas W. F.Bortolotti, LisaMitulovic, GoranBortolotti, UbertoMiu, Adriana S.Bortoluzzi, CristianoMiura, MizukiBorys, MichałMixon, AmandaBos, Lieuwe D. J.Miyachi, HidekiBos, ManonMiyama, TakeshiBosch, RicardoMiyamoto, HiroshiBoschetti, GiovanniMiyamoto, KyoheiBose, BhadranMiyamoto, NobukazuBosello, Silvia LauraMiyamoto, TakeshiBoskey, Elizabeth R.Miyamoto, YoheiBosman, Conrado A.Miyasaka, MasakiBosnjak, Andrija PetarMiyata, YasuyoshiBosnjak, BerislavMiyazaki, AyaBossé, YnukMiyoshi, TakekazuBossu, MaurizioMizia-Stec, KatarzynaBoström, Kristina BengtssonMizota, ToshiyukiBotchu, RajeshMizukami, KazuhiroBotelho, JoãoMizumoto, KenjiBotella, Luisa MaríaMizuno, KohBotta, AnnalisaMizuno, MasashiBottagisio, MartaMizuno, ShihoBöttcher, BettinaM'Koma, AmosyBottenberg, PeterMlaga, Kodjovi D.Botticelli, DanieleMlynarska, AgnieszkaBottinor, WendyMlynarski, RafalBottio, TomasoMo, Clifton C.Boubred, FaridMo, FeiBoufraqech, MyriemMoaven, OmeedBoukhibar, Lamia MestekMoazzen, SaraBoukpessi, TchilaloMobley, David F.Boulouis, GrégoireMoccia, MarcelloBouloukaki, IzoldeMocciaro, FilippoBouma, Berto J.Mocco, Jay D.Bourcier, RomainModesti, Pietro A.Bourdel, NicolasMoffatt, PierreBourdin, ArnaudMogilski, SzczepanBourdon, EmmanuelMohamed, Salah A.Boureau, Anne SophieMohamed, Tarek MagdyBourgeois, DenisMohammed, AkramBourlet, ThomasMohan, Babu P.Bourque, Daniel LeeMohiddin, Saidi A.Bourret, VincentMöhlenbruch, MarkusBoursier, GuilaineMohr, AliciaBoutros, JacquesMoik, FlorianBouts, AntoniaMoiseev, Roman V.Boutsiadis, AchilleasMojtahedi, ZahraBouvier, Anne-MarieMoktefi, AnissaBouvier, DamienMoles, LauraBowles, Kristian M.Moletta, LuciaBowry, RitvijMolina-Cerrillo, JavierBoyages, JohnMolins, BlancaBoyden, Penelope A.Molitch, MarkBoylan, KhristaMollace, VincenzoBozic, JoskoMoller, Ann MereteBozzini, SaraMollica, MichelleBraatz, FrankMolmenti, Ernesto P.Bracero, Luis A.Momi, StefaniaBracht, Jillian Wilhelmina PaulinaMomose, HitoshiBradbury, AllisonMona, MahmoudBradley, DavidMonacelli, FiammettaBrady, Mary S.Monaco, AnnalisaBrady, MollyMonaghan, ThomasBraekkan, SigridMonastero, RobertoBraga, AndreaMonavarfeshani, AboozarBraga, SofiMonchatre-Leroy, ElodieBragazzi, Nicola LuigiMond, HarryBrainson, ChristineMondaca, SebastiánBraithwaite, TasaneeMondal, PritishBrambilla, MarcoMondal, Utpal KumarBramham, KateMondejar-Lopez, PedroBramley, KyleMondet, JulieBramness, Jørgen GustavMondini, MicheleBramuzzo, MatteoMongillo, MarcoBranca, Jacopo Junio ValerioMonib, SherifBrancaccio, MariaritaMoniche, FranciscoBrand, SergeMonickaraj, FinnyBrandl, AndreasMoniuszko, AnnaBrandt, ChristianMonji, AkiraBrantley, Milam AMonllau, Juan CarlosBraquehais, María DoloresMonnier, PatriciaBrault, Jeffrey J.Monroe-DeVita, Maria B.Braun, SilkeMonroy Gonzalez, AndreaBräunlich, JensMonsarrat, PaulBraverman, EricMonsen, Anne Lise BjørkeBravo, IsabelMontag, DirkBray, IssyMontaruli, GrazianoBrehm, AlexMonteagudo, AndreaBrehm, Maria AlexandraMontecucco, FabrizioBreik, OmarMontero, ÁngelBrekk, OeysteinMontero, EduardoBrémond-Gignac, DominiqueMontgomery, Courtney GrayBrennan, ElleMontgomery, ErwinBrennan, PaulMontisci, AndreaBressem, Keno-KyrillMontminy, Eric M.Bretelle, FlorenceMontone, Rocco AntonioBretschneider, Carol EmiMontorio, DanielaBreuker, AnjaMoon, Suk-HoBrewster, LukeMoonesinghe, RamalBriana, Despina D.Moore, Axel C.Briassoulis, GeorgeMoore, KarenBridgewater, DarrenMoore, RodBrier, Michael E.Moore, Ronald BBrierly, Gary I.Moore, Shona C.Briggs, FarrenMorabito, AntoninoBrindisi, MatteoMoraitis, AlexandrosBrini, Anna T.Morales, HumbertoBrinker, Jeffrey A.Morales, SerafinBrinkmann, ChristianMoraska, Albert F.Brinkmann, MaxMörchen, ManfredBritt, Rodney D.Morciano, GiampaoloBrkic, Faris F.Morel, OlivierBrocco, EnricoMorel-Kopp, Marie ChristineBrodehl, AndreasMorelli, LorenzoBrodmann, MarianneMorelli, LucaBrodosi, LuciaMorelli, MarcoBroek, Richard P. G. TenMoreno-Navarrete, José MaríaBroggi, MorganMoreso, FrancescBrogna, ClaudiaMoretti, BiagioBrosens, ErwinMoretti, MatteoBrotfain, EvgeniMorga, RafałBrown, Austin L.Morgan, IanBrown, Belinda M.Morgan, KatieBrown, Eric L.Morgenstern, ChristianBrown, GregoryMori, HidekiBrown, Joshua D.Mori, HiroyoshiBrown, Joshua DavidMori, KiyoshiBrown, RichardMori, MasaakiBrown, Ronald B.Morichi, ShinichiroBrown, Scott DerekMorillo, Daniel SánchezBrown, Steven R.Morimoto, KozoBrowne, AndrewMorimura, SohshiBrowne, MarilynMorin, MathieuBrownstein, Zippora (Zippi)Morino, TadaoBruce, DavidMorinobu, AkioBruchfeld, AnnetteMorio, YoshiteruBrucker, Sara Y.Moris, LisaBrucoli, MatteoMorita, YasuyukiBrudnicki, AndrzejMoritz, Michael L.Brugliera, LuigiaMoriya, ManabuBrügmann, TobiasMoriyama, NoriakiBrugts, Jasper J.Morlet, ThierryBruhn-Olszewska, BozenaMorling, Joanne R.Bruinsma, BoteMornos, CristianBrukner, IvanMoro, EnricoBruland, TorunnMoroni, LucaBrun, Jean-FrédéricMorosini, MonicaBrunetti, OronzoMorra, FrancescoBrunetti, ValerioMorris, Brian J.Brungs, DanielMorris, ClaudiaBrunne, MaximilianMorris, DavidBruns, Danielle R.Morris, JodieBrunt, ElizabethMorrison, Brett M.Brunt, Elizabeth M.Morrison, PeterBrusciano, LuigiMorseth, Marianne SandsmarkBruscolini, AliceMorsica, GiuliaBruserud, ØysteinMortelmans, LucBrusini, PaoloMortensen, EricBruzzese, DarioMortiboys, HeatherBruzzese, FrancescaMortimer, PeterBryant, KimberleyMorton, EmmaBryndza, Magdalena A.Moschovi, MariaBryniarski, Mark A.Moscicka, PaulinaBrzecka, AnnaMoscucci, FedericaBubb, KristenMoses, Jeffrey W.Bublitz, MargaretMosqueira, DiogoBucci, MarcoMoss, Esther LouiseBucci, RosariaMostofa, Agm G. M.Buchanan, BrianMotamed, CyrusBuchbinder, ElizabethMotomura, NoboruBuchet, ReneMotoo, YoshiharuBuchholz, MirkoMotwani, MonaBuchhorn, ReinerMou, HaiweiBuchs, NicolasMoulana, MohadethehBuchta, ChristophMoulier, VirginieBuchtele, NinaMoulin, ValerieBuckalew, NeillyMounien, LourdesBuckert, DominikMount, Peter F.Bucki, RobertMoura-Coelho, Nuno.Buckley, RichardMourits, Maarten PhBuckley, Shannon M.Moursy, MohamedBucolo, ClaudioMourtzinis, GeorgiosBudde, KlemensMoussa, DinaBudge, Philip J.Movahed, AssadBudimirovic, DejanMozas-Moreno, JuanBudzyń, MagdalenaMozos, IoanaBuechter, MatthiasMráz, JaroslavBuga, Ana-MariaMucci, ArmidaBugajski, AndrewMueller, Sarina K.Bugajski, MarekMueller, SvenBugge, Thomas H.Müeller, ThomasBuisson, RémiMueller, Wolf C.Buja, L. MaximilianMugisho, OdunayoBujak-Gizycka, BeataMuhammad, NaoshadBujan, JuliaMuhsen, KhitamBujold, EmmanuelMuiesan, Maria LorenzaBukavina, LauraMuir-Cochrane, EimearBukhari, Marwan A. S.Mukaetova-Ladinska, Elizabeta BBukkuri, AnuraagMukherjee, DwaipayanBuljac, MartinaMukherjee, KusumikaBullens, Dominique M. A.Mukherjee, NabanitaBuller, HarryMukherjee, RahulBultot, LaurentMukherjee, RatnadeepBundschuh, Ralph A.Mukherjee, SarbajitBunge, Jeroen J. H.Mukherjee, SudipBunn, JenniferMukhopadhyay, SubhradipBuonamici, FrancescoMulhearn, BenBuonsenso, DaniloMulier, Jan PaulBurashnikov, AlexanderMuller, JacobusBuratti, EmanueleMuller, MartinBuratti, LauraMüller, NorbertBurchiel, Kim J.Müller, NotgerBurdukiewicz, MichałMüller, PatrickBurg, Matthew M.Müller, RüdigerBurger, Michael C.Müller, ThomasBurggraaf, Koos JacobusMüller-Decker, KarinBurggraf, ManuelMüller-Edenborn, BjörnBurgos Alonso, NataliaMullier, FrancoisBurgos-Artizzu, Xavier P.Mullier, FrançoisBurgunder, Jean-MarcMullins, Robert F.Burkard, NatalieMulloy, BarbaraBurkard, ThiloMulvihill, ErinBurke, Thomas F.Mulya, AnnyBurkhalter, HannaMun, Je-HoBurns, KatherineMunakata, SatoruBurns, Stephen A.Mundo, LuciaBurrello, JacopoMuniyan, SakthivelBurris, Heather H.Munker, ReinholdBursztyn, Lulu L. C. D.Muñoz, Cynthia E.Burton, Claire L.Munro, AlisonBurucoa, ChristopheMunshi, SoumyabrataBusch, AlbertMünstedt, KarstenBusch, MartinMunusamy, ShankarBussel, James B.Munz, MatthiasBussotti, MaurizioMünzker, JuliaBustos, RobertoMurab, SumitBustuchina Vlaicu, MihaelaMurakami, NaokaBusuttil, Rita A.Murakami, TomoakiBuszko, KatarzynaMurakami, YoshikiButt, Julia A.Murakhovskaya, IrinaButt, WarwickMuraleedharan, Ranjith MenonButterworth, ChrisMuramatsu, TakashiButtò, StefanoMurashima, MihoBuus, Niels HenrikMurdaca, GiuseppeByeon, HaewonMurdica, ValentinaByrne, GregMurea, MarianaCaba, OctavioMuriach, MaríaCabanas, HeleneMuro, ShigeoCabello, MariaMurotani, KentaCabeza, LauraMurphy, AndrewCabiati, ManuelaMurphy, BernadetteCabrera, SílviaMurphy, GregoryCabrera, Wellington M.Murphy, RonanCabrera-Munoz, NestorMurphy, Susan L.Caccianiga, GianluigiMurray, Peter E.Cacciola, IreneMurray-Leisure, KatherineCacciottola, LucianaMurri, RitaCáceda, RicardoMurthy, Guru SubramanianCacopardo, BrunoMurtinger, MaximilianCadamuro, MassimilianoMuruganandan, ShanmugamCadeddu Dessalvi, ChristianMusialik, Joanna ACademartiri, FilippoMusk, BillCadenaro, MilenaMusolf, AnthonyCadranel, Jean François D.Mußbacher, MarionCaffarelli, CarlaMusso, NataleCaggia, SilviaMustafa, TehminaCaggiano, GiuseppinaMusu, DavideCaggiati, AlbertoMusumeci, GiuseppeCagnacci, AngeloMutalib, MohamedCai, JiyangMuthukumar, Alagar R.Caiati, CarloMutis, TunaCaignard, AnneMutoh, TatsushiCairrão, ElisaMutti, LucianoCalabrese, GiovannaMuzzafar, TariqCalabrò, PaoloMyers, Alyson K.Calabuig-Farinas, SilviaMyers, StephenCalañas, AlfonsoMyles, Ian A.Calcaterra, IleniaMynampati Arunadithya, Bharani KrishnaCaldarola, GiacomoMyung, DavidCalderón De Anda, FroylanN. Balamurugan, AppakalaiCaldovic, LjubicaNa, Dong GyuCaldwell, R. WilliamNa, KiyongCalinescu, AlexandraNa, KunCallejas, AntonioNabavizadeh, RezaCalle-Pascual, Alfonso L.Nabeta, TakeruCalle-Pascual, Alfonso LuisNaccache, Jean-MarcCaltabiano, RosarioNaccarato, MarcelloCalvet, XavierNadeem, OmarCalvisi, Diego FrancescoNagai, NoriakiCalvo, Maria Icíar ArteagoitiaNagai, NorihiroCalvo-Escalona, RosaNagai, YoshioCalvo-Lobo, CésarNagakura, YukinoriCamacho-Alonso, FabioNaganuma, ToshihideCamafort-Babkowski, MiquelNagarajah, JamesCambi, AlessandraNagasaka, KazunoriCameli, PaoloNagata, KoseiCameron, EmilyNagatomo, YujiCaminati, AntonellaNägele, Matthias P.Cammalleri, ValeriaNagler, ArnonCammisuli, Davide MariaNaguib, TarekCamoni, LucaNah, Deuk-YoungCampagna, GiuseppeNahéma, IssaCampana, Luca GiovanniNahum, AmitCampbell, KathleenNaidu, Samisubbu R.Campello, ElenaNaik, AkshataCampi, RiccardoNaik, Bhiken I.Campioni, IlariaNaiki, TakuCampise, MariarosariaNair, Girish B.Campisi, GiuseppinaNair, RanjitCampochiaro, CorradoNaito, RyoCampos, Camila D. MadeiraNajafi, NadiaCampos, Michael A.Nakachi, TatsuyaCampra, DonataNakagawa, TohruCamps, GuidoNakai, MichikazuCamps-Font, OctaviNakai, YousukeCampuzano, OscarNakajima, KeiCañas, José A.Nakajima, YasuakiCanavese, FedericoNakamoto, TakashiCancelas, Jose A.Nakamura, AkinobuCaneiras, CátiaNakamura, HidekiCanepa-Escaro, FabrizioNakamura, KensukeCangiano, BiagioNakamura, MasanaoCannella, RobertoNakamura, MasayaCano, AinaraNakamura, MotokiCano, David A.Nakamura, TomokiCano-García, Francisco JavierNakanishi, MahitoCantarella, FrancescoNakanishi, NaohikoCantarero-Villanueva, IreneNakano, KenjiCantor, JonathanNakano, MasahitoCanullo, LuigiNakano, YukikoCaocci, GiovanniNakase, HiroshiCapala, JacekNakashizuka, HiroyukiCapaldo, B.Nakaya, NaokiCaparello, ChiaraNakayama, JiroCaparros-Gonzalez, Rafael A.Nakayama, JunCaparrós-González, Rafael ArcángelNakazawa, MinatoCapasso, VincenzoNakhjavani, MaryamCapek, Karel D.Nalesso, FedericoCapel, FredericNam, Hae-SeongCapela E Silva, FernandoNam, WoongCapetanaki, Yassemi G.Nam, Young-HeeCapoccia, MassimoNamba, FumihikoCapone, AntonioNamihira, MasakazuCaponio, Vito Carlo AlbertoNamisaki, TadashiCapoulade, RomainNanda, NehaCapozzi, AnnaNanda, VivekCapozzi, Vito AndreaNap, AnnemiekCapparè, PaoloNapolioni, ValerioCappellano, GiuseppeNapolitano, MariasantaCappelletti, VeraNappi, Anna GiuliaCappon, GiacomoNarang, AkhilCappuccilli, MariaNaranje, SameerCappuccio, SerenaNarasimhulu, Chandrakala AlugantiCaprari, PatriziaNarayan, PrakashCaprini, JosephNarayan, Ram NarendraCaptur, GabriellaNardini, ChristineCapurso, GabrieleNarloch, JerzyCaputi, MassimoNaro, AntoninoCaputi, ValentinaNaruishi, KojiCarames, João Manuel MendesNarzisi, AntonioCarbone, AntonioNashine, SonaliCarbone, JavierNasierowska-Guttmejer, AnnaCarbone, SalvatoreNasiłowski, JacekCarbone, StefanoNasios, GrigoriosCarboni, FabioNasioudis, DimitriosCardali, Salvatore MassimilianoNasry, WalaaCardenas, Jessica C.Nasser, Nicola J.Cardoso, LuísNassisi, MarcoCardozo, Erwing FabianNassour, IbrahimCarducci, ClaudiaNastiuk, Kent L.Carella, Angelo MNatale, FrancaCarey, John J.Natale, VincenzoCariati, FedericaNatarajan, SivaramanCarl, EmilyNath, ShubhankarCarlan, SteveNathan, NadiaCarle, FlaviaNatile, GiovanniCarli, FrancescoNatsis, KonstantinosCarlier, StephaneNatsuko, ChibaCarlitz, Ruth D.Naud, PatriceCarll, AlexNaugler, ChristopherCarlsen, EsbenNava, ElenaCarlson, DouglasNavarrete, CristianCarlucci, AnnalisaNavarro Flores, EmmanuelCarmalt, James L.Navarro, EstanisCarmona Mejías, RitaNavarro, Jose FranciscoCarnagarin, RevathyNavarro-Flores, EmmanuelCarona, CarlosNavasiolava, NastassiaCaroppo, EttoreNawaz, MuhammadCarosso, Andrea R.Naydenov, StefanCarotti, SimoneNdrepepa, GjinCarow, BeritNebe, StephanCarpenter, GuyNedios, SotiriosCarpenter, LewisNedkoff, LeeCarrabba, NazarioNedoszytko, BoguslawCarreau, Anne-marieNeeb, AntjeCarrera, PilarNegoro, HiromitsuCarricajo, AnneNegrea, AdinaCarriedo, AlejandroNegroni, LucCarrier, PaulNelson, Amanda M.Carrizzo, AlbinoNelson, GreggCarro, EvaNelson, MaxCarroll, ThomasNelson, Michael D.Carron, MicheleNelson, Richard LCarter, BenjaminNemeș, Roxana MariaCarugno, JoseNemeth, BanneCarulli, ChristianNemeth, Zoltan HCarullo, GabrieleNenna, AntonioCaruso, GiuliaNeri, ManuelaCarvalho, Ana Sofia LeitãoNeri, MatteoCarvalho, CristinaNerla, RobertoCarvalho, Hugo N.Nesbitt-Hawes, ErinCarvalho, Lina A. S.Neshatian, LeilaCary, LynnetteNestelberger, ThomasCasadei, RiccardoNestor, MelissaCasadevall, ArturoNestrasil, IgorCasado, AlfonsoNetchiporouk, ElenaCasagrande, MariaNetherton, Stuart J.Casalino, GiuseppeNeuerburg, CarlCasanova, Emily L.Neufeld, Susan M.Cascone, PieroNeuhaus, NinaCascone, RobertoNeuman, ManuelaCasella, GiovanniNeumann, Johannes TobiasCasey, Jonathan DaleNeumann, ThomasCasey, PatriciaNeumann, TimCasini, GiambertoNeumann, Ulf PeterCasper, Regina C.Neunaber, ClaudiaCasserly, IvanNevalainen, Mika T.Cassetta, MicheleNeves, BrunoCassi, DianaNewall, Philip W. S.Cassol, ClarissaNeymotin, FlorenceCassone, G.Neyt, JeroenCastaldi, AlessandraNg, ChinCastel, Arturo LópezNg, Chong ShenCastelino, RonaldNg, RayCastellana, MarcoNg, Wai-LungCastellani, DanieleNguyen, AnnieCastellanos, Miguel ÁngelNguyen, JustinCastelli, DarlaNguyen, LinhCastellino, PietroNguyen, Minhhuyen T.Castellví, IvánNGUYEN, PhilippeCastelo-Branco, MiguelNguyen, Thi-HuongCastillo Olea, CristianNguyen, ThomasCastillo, SilviaNgwa, Che JuliusCastorina, AlessandroNiaz, TalhaCastro Alves, RicardoNicastro, NicolasCastro, Ana B.Nichetti, FedericoCastro, GraciaNicholas, JenniferCastro, PedroNicholas, LaurenCastro-Alonso, CristinaNicholls, DashaCastro-Giner, FrancescNichols, Frank C.Casu, CinziaNicholson, Ainsley C.Casu, GavinoNicholson, KathrynCasu, GiuliaNickbakhsh, SemaCasuso-Pérez, RafaelNickel, BrookeCatalano, AntoninoNickel, ChristianCataldi, AmeliaNickel, KathrinCate, Hugo TenNickinson, AndrewCaterino, UmbertoNicod-Lalonde, MarieCatic, Angela GeorgiaNicolai, MicheleCatlow, SarahNicolaides, StevenCaturano, AlfredoNicolakakis, NektariaCauli, OmarNicolas-Silvente, Ana IsabelCavagnetto, DavideNicole, GrünCavalcanti, Bruno NevesNicoletti, LoredanaCavaliere, CarloNidorf, Stefan M.Cavallari, LarissaNiederseer, DavidCavallaro, GiuseppeNielsen, BirtheCavalli, LoredanaNielsen, Elisabet I.Cavallo, ArmandoNielsen, Mette KjaergaardCavallo, MarcoNielsen, NathanCavassini, MatthiasNielsen, Vance GirardCavayas, Yiorgos AlexandrosNielsen-Kudsk, Jens ErikCave, John W.Niemczyk, MariuszCavestro, Giulia MartinaNiemira, MagdalenaCaviggioli, FabioNier, AnikaCaviglia, Gian PaoloNiethammer, Thomas RichardCavo, MartaNieto Fontarigo, Juan JoseCavodeassi, FlorenciaNieto, RubénCavoretto, PaoloNighot, PrashantCeballos-Laita, LuisNihalani, DeepakCebrià I Iranzo, Maria Dels ÀngelsNii, MasafumiCeccato, AdriánNijakowski, KacperCederroth, Christopher R.Nijhof, IngerCeesay, Muhammed MansourNijs, JoCegielska, OlgaNikaki, AlexandraCela, VitoNikolaos, MachairasCelentano, ValerioNikolaou, Charoula KonstantiaCeli, AlessandroNikolopoulos, GeorgiosCella, EleonoraNilsson, Björn MikaelCelletti, ClaudiaNilsson-Wikmar, LenaCelsa, CiroNilubol, NarisCengel, KeithNilvebrant, JohanCengiz, Ibrahim FatihNimbi, FilippoCensi, SimonaNinni, SandroCento, ValeriaNirwane, AbhijitCentola, MarcoNishi, HiroshiCerdán, María-EsperanzaNishida, NaokiCerejeira, JoaquimNishida, ToshiroCerletti, ChiaraNishida, TsutomuCerny, JanNishikawa, TetsuoCerretti, RaffaellaNishikimi, KyokoCervantes, JorgeNishikimi, MitsuakiCervera Juanes, Rita PNishikiori, HirotakaCervera, AnaNishimura, ReikiCervera-Garvi, PabloNishino, TomofumiCerwenka, HerwigNishio, MizuhoCesareo, RobertoNishio, ShinCesaro, ArturoNishioka, ShintaCestmir, AltanerNishiwaki, MasatoChaber, RadosławNishiyama, MasahiroChae, Jung-wooNishizawa, DaisukeChae, Min SukNisolle, MichelleChaitanya, Ganta VijayxNissan, JosephChakrabarty, SamitNissim, SaharChakrabarty, TrishaNiu, TianhuaChakraborty, AmlanNkire, NnamdiChakraborty, SandipanNobel, YaelChalah, Moussa AntoineNobile, StefanoChalla, DilipNobles, JamesChallis, Benjamin G.Nobre Cardoso, JoãoChambellan, ArnaudNobre, Miguel De AraújoChambers, Melissa RenéeNocini, RiccardoChami, BelalNoel, Christopher W.Chamorro, Antonio-JavierNoflatscher, MariaChan, AlexanderNogami, KeijiChan, HenryNogler, Michael M.Chan, Kevin C.Noguchi, TeruoChan, StanleyNogueira, Paulo JorgeChan, WinnieNolan, DavidChand, Hitendra SinghNolde, Janis MarcChander, HarishNold-Petry, Claudia A.Chandler, HeatherNoll-Hussong, MichaelChandler, Wayne L.Nolte, Christian H.Chandrasegaram, ManjuNoma, HidetakaChandrasekar, AkilaNonoyama, Mika L.Chandrasekhar, AnandNoonan, VikkiChang, Andrew A.Nopp, StephanChang, Emily H.Nordio, MaurizioChang, EricNordmann, PatriceChang, HongNordmark, GunnelChang, Jae HyunNorén, TorbjörnChang, Kun-CheNorgan, Andrew P.Chang, Mary P.Norgren, LarsChang, Min CheolNoriega, CristinaChang, Shuo-HsiuNorman, TrevorChang, StanleyNormann, ClausChanna, RoomasaNorrbom, JessicaChantzichristos, DimitriosNorton, Kerri-AnnChapman, RyanNorton, NadineCharalampopoulos, LoannisNosotti, Maria GiuliaCharbonneau, MichelNotas, GeorgeCharest-Morin, RaphaëleNothlings, UteCharlewood, RichardNouri, AriaCharlier, CarolineNoutsias, MichelCharman, William NeilNovák, JanCharmsaz, SaraNoviello, CarmineCharoenngam, NipithNovío, SilviaChatterjee, ArkaNowak, GlenChatterjee, IshitaNowakowska-Zajdel, EwaChatzellis, EleftheriosNowbar, Alexandra N.Chatzikyrkou, ChristosNowicka, AlicjaChaudhry, RajeevNowicki, MichałChaudhuri, DipayanNowomiejska, KatarzynaChaudhuri, NaziaNozaki, FumihitoChauhan, MunishNtanasis-Stathopoulos, IoannisChauhan, NeerajNuara, ArturoChaushu, GavrielNucci, PaoloChaushu, StellaNucera, EleonoraChavoin, Jean-PierreNuessler, AndreasChawla, SumirNunes, Ane Claudia FernandesCheak-Zamora, NancyNunes, MónicaCheasley, DaneNúñez-Pereira, SusanaChecchi, VittorioNurmi, Erika L.Chedid, VictorNurmohamed, M. T.Cheemarla, Nagarjuna ReddyNurwidya, FarizChehroudi, BabakNussinovitch, UdiChemouny, Jonathan M.Nüssler, AndreasChen, BinNüssler, Andreas K.Chen, DongNuvoli, SusannaChen, Emerson Y.Nuytemans, KarenChen, GuifenNwosu, Benjamin UdokaChen, GuoxunNyangoh Timoh, KrystelChen, HongyuNyante, SarahChen, Inna MarkovnaNyati, Kishan KumarChen, JennyNyberg, AndreChen, Jian-MinNylander, SvenChen, JianzhongO. Othman, MohamedChen, JingO’Beirne, JamesChen, Ji-YihO’kane, MauriceChen, Kong Y.O’Neal, SuzanneChen, QiObermayr, EvaChen, ShanquanObolski, UriChen, ShaojieO'Brien, LisaChen, Shing-JyeO'Brien, Timothy E.Chen, TingtingObuchi, ChieChen, Vincent L.Ocak, SebahatChen, XiO'Callaghan, JeffreyChen, Xi StevenOcchetta, EraldoChen, XingchengOchayon, David E.Chen, XiongwenOchoa, SusanaChen, YangOchsenkühn, ThomasChen, Yu-ChunO'Connell, GraceChen, ZhaojingO'Connor, RaymondChendrasekhar, AkellaOczko-Wojciechowska, MałgorzataCheng, ChunmingOdom, VernonCheng, HaiziOe, MakatoCheng, HaoOetjen, ElkeCheng, JiaOgawa, ShumpeiCheng, JizhongOgawa, TakahiroCheng, Phil F.Ogawa, TakahisaCheng, ShibinOgawa-Ochiai, KeikoCheng, Yuen YeeOgden, AllisonCheon, Dong-JooOgino, ShujiCherchi, MarcelloOgunjimi, Abayomi T.Cherian, Sujith.V.Ogura, TakayukiChesnais, Cédric B.Ogura, TakeshiChesnutt, MarkOh, ByeongsangCheson, Bruce D.Oh, JaehoonCheung, AdaOh, Je HyeokCheungpasitporn, WisitOh, Joo YounChevalier, FrançoisOh, JungHwanChhabra, GaganOh, Scott S.Chi, GeraldOh, Se-LimChiang, Tung-chinOh, SewonChieppa, MarcelloOh, SooyoungChigbu, De Gaulle I.Oh, SoramChilimuri, Sridhar S.Oh, Sung YongChilla, AnastasiaOh, Yong-SeogChinegwundoh, FrancisO'Hagan, HeatherChinitz, Jason S.O'Hare, LouiseChintalapudi, SumanaOhashi, NaroChiosi, FlaviaOhba, ShigeoChiou, Shin-YiO'Higgins, AmyChipollini, Juan J.Ohira, MasahiroChirkov, Yuliy Y.Ohishi, TomokazuChistolini, AntonioOhki, MasafumiChitale, ShalakaOhko, KentaroChittiboina, PrashantOhlander, Samuel J.Chiumello, DavideOhlin, AckeChiva-Blanch, GemmaOhlmann, BrigitteChizzolini, CarloOhshima, TomokoChmielewska, DariaOhta, TakahisaChmielowiec, KrzysztofOhtsuka, TakashiChmielowski, BartoszOjima, ToshiyasuCho, HanjinOk, Soo-MinCho, Hwi-youngOka, SatokoCho, InOkada, SeigoCho, Jai YoungOkajima, HideakiCho, Kang SuOkamoto, MotokiCho, Kwang ReeOkamura, TakuroChoi, InhoOkayama, YoshimichiChoi, Ji-YeobOkayasu, IsaoChoi, Joon YoungOkazaki, TomoyaChoi, Kang YoungOkochi, MinaChoi, MichaelOks, MargaritaChoi, Seong HyeOksi, JarmoChoi, Seong-SooOku, HidehiroChoi, WonOkuda, KatsuhiroChoi, Woo JuneOkuda, NagakoChoi, WungrakOkumura, TakahiroChoi, Yoon JeongOlander, Ellinor K.Cholkar, KishoreOlasińska-Wiśniewska, AnnaCholongitas, EvangelosOłdak, MonikaChomicz, LidiaOldfield, BrianChong, Gun OhO'Leary, Valerie BrídChoo, Myung-SooOlena, OliveiraChoonara, ImtiOleszko, AdamChoong, PeterOliva, AntonioChoromańska, BarbaraOliva, JoanChou, Tsung-HanOlivari, LauraChoudhary, TusharOliveira Carvalho Rigaud, VagnerChoudhury, DevasmitaOliver Heinrich, FelthausChowdhury, DevyaniOlivera, MarcoChowdhury, IndrajitOliveria, John PaulChowdhury, SourangsuOlmedillas, HugoChowell, GerardoOlmedo-Requena, RocíoChrcanovic, BrunoOlms, ConstanzeChristen, UrsOlofsson, Jan I.Christensen, Louisa MargueriteOlomu, IsokenChristensen, Mark SchramOlsen, Niels ThueChristensen, RobinOlson, KristineChristiansen, Ole BjarneOlson, SarahChristidi, FoteiniOlsson, Erik Martin GustafChristie, StewartOlszewska, AnetaChristodoulides, MyronOlszewska, MartaChristodoulou, Maria-IoannaOmarjee, LoukmanChristoffersen, MetteOmori, TeppeiChristou, Georgios A.Omran, HazemChristou, TerpsitheaOmran, HeymutChrysanthakopoulos, Nikolaos A.Oña Simbaña, Edwin DanielChu, LilyOnder, GrazianoChu, ZhongdiO'neill, FrancisChua, Eng GuanO'Neill, Stacey S.Chua, Joel V.Ong, Adrian W.Chuang, Alice Z.Ong, TerenceChuang, Ming-LungOno, TakashiChudek, JerzyOno, YukoChun, JaeyoungOnofrillo, CarmineChung, FrancesOntoria-Oviedo, ImeldaChung, JinOO, Joo HyunChung, Jong WooOosterlinck, WillemChung, Ki-WookOosterwijk, EgbertChung, Yong AnOpel, NilsChung, Yoo-RiOpie, George M.Church, James M.Opitz, AntjeChvanov, MichaelOrban, MartinChwalisz, BartO'Reilly, Michael A.Chyrchel, MichałO'Reilly, StevenCianci, PasqualeOria, MarcCiancio, GaetanoOriol, AlbertCianciulli, AntoniaOrlacchio, ArturoCianflone, DomenicoOrnello, RaffaeleCiappolino, ValentinaOronce, Carlos Irwin A.Ciardi, Maria RosaOrr, Miranda E.Ciccarelli, MariaOrssaud, ChristopheCicciù, MarcoOrtega, Miguel AngelCicero, ArrigoOrtel, BernhardCicero, GiuseppeOrtensi, LucaCichorek, MiroslawaOrtiz, AlbertoCici, DanielaOrtiz, AndricCiclitira, PaulOrtiz, JorgeCieri-Hutcherson, NicoleOrtiz, MiriamCieslewicz, ArturOrtiz-Toquero, SaraCieślik, BłażejOry, JesseCieślińska, AnnaOsadnik, TadeuszCilluffo, GiovannaOsaki, TakakoCimadamore, AlessiaOsaki, YoshinoriCimmino, GiovanniOsanai, HiroyukiCinone, NicolettaOsawa, SatoshiCinotti, ElisaOsborn, Stephanie L.Cinque, BenedettaOsborne, JaneCiocca, LeonardoOsborne, NickCioffi, IolandaO'Shaughnessy, RyanCioni, GiovanniO'Shea, AileenCipolli, MarcoO'Shea, AmyCipollini, VirginiaOshima, YujiCippà, PietroOstacolo, CarmineCipriani, AlbertoOsto, ElenaCirami, Calogero LinoOstrowska, BożenaCirer-Sastre, RafelOsuna, EduardoCiria, RubénOtani, KojiCiriaco, PaolaOtani, ShinjiCirillo, MarcoOteo, José A.Cirillo, PlinioOthman, Ahmed E.Cisneros, José MiguelO'Toole, Sharon A.Citro, SimonaOtsuki, MasayukiCiudin, AndreaOtt, JohannesCiuffreda, LudovicaOttaviani, StefaniaCives, MauroOttolenghi, SaraClaes, JommeOualha, MehdiClain, Jeremy M.Oudman, ErikClairambault, JeanOuro, AlbertoClapham, HannahOuzaid, IdirClarkson, Becky D.Overtchouk, PavelClaus, ClaudiaOvesen, PerClausen, JohannesØvrehus, Marius AClay, Brian J.Owen, Patrick J.Cleare, AnthonyOwens, Anjali TikuCleland, John G. F.Owens, CormacClemens, Robert K.Owens, P.Clemens, VeraOzaki, ShujiClement, N. D.Ozawa, YokoClerico, AldoÖzdemir, Berna C.Clough, MeaghanOzga, AndrewCoates, Matthew D.Özkurtul, OrkunCoats, LouiseOzturk, ArincCobb, Charles M.Öztürk, CanCobb, SharonPabst, Andreas MaxCobbin, JoannaPacifici, LucianoCoccaro, NicolettaPacifico, Francesco M.Coccheri, SergioPaciotti, MicheleCochetti, GiovanniPackiam, Vignesh T.Cockrell, Robert CPaczesny, ŁukaszCocuzza, SalvatorePadala, Sandeep A.Coelho, AnaPadilla, MarielaCoelho, Joana E.Padilla-Eguiluz, Norma G.Coelho, TracyPados, GeorgeCoenen, MaraikePadua, LucaCoeugniet, EdouardPaduch, RomanCoffey, Michael J.Paepegaey, Anne-CécileCognasse, FabricePafundi, Pia ClaraCohen, Jeffrey M.Paganelli, FranckCohen, Michael S.Pagano, FrancescaCohen, PinchasPagano, GiovanniCohen, SethPagano, IanCoheña-Jimenez, ManuelPagano, SabrinaCohn, ClaudiaPagano, StefanoCohrdes, CarolinePage, MelissaCohrs, Randall J.Paglia, DavidColasanti, RobertoPagonopoulou, OlgaCole, DavidPagourelias, Efstathios D.Cole, Emily D.Pahl, HeikeColeman, CynthiaPai, HyunjooCollado-Mateo, DanielPaiella, SalvatoreCollart-Dutilleul, Pierre-YvesPailhoriès, HélèneCollery, Ross F.Paillard, CatherineCollet, Jean-paulPaisey, Richard BayleyCollier, AndrewPajevic, Paola DivietiCollins, Eileen G.Pajkrt, EvaCollins, Justin W.Pak, KyoungjuneColmenero, IsabelPal Choudhuri, ShreoshiColoma-Carmona, AinhoaPAL, AJAYColombatto, PieroPalaiodimos, LeonidasColquhoun, SamanthaPalaniswamy, SaranyaColson, Serge S.Palau, PatriciaColucci, FrancescoPalazón-Bru, AntonioColucci, GiuseppePaley, Carole A.Colunga Biancatelli, Ruben Manuel LucianoPalioura, SotiriaColuzzi, DonaldPalladini, GiuseppinaComai, GiorgiaPallag, AnnamáriaCometi, LauraPalma, Paulo JorgeCompagnon, RoxanePalmeira-de-Oliveira, AnaCompan, ValériePalmer, AlanCompare, DeboraPalmer, Jacqueline A.Conaghan, PhilipPalmer, MichelleConde, BebianaPalmieri, Lola JadeConil, Jean-MariePalomo López, PatriciaConley, ShannonPalser, Eleanor R.Connaughton, Victoria P.Palumbi, RobertoConnolly, ChristopherPalumbo, AlessioConover, Cheryl A.Palumbo, BarbaraConrad, David MPalumbo, CarlottaConroy, DominicPalumbo, Vincenzo DavideConserva, EnricoPaluszkiewicz, LechConsolo, UgoPamir, NathalieConstant, AymeryPammett, Robert T.Contag, StephenPamoukdjian, FredericContaldo, MariaPampena, RiccardoConte, Antonio HernandezPan, HongyingConte, FerruccioPan, HuizeConti, MichelePan, MingguiConti, SaraPanagiotou, GrigoriosContiero, PaoloPanarese, AlbaContin, MauelaPanatala, RadhakrishnanConversi, DavidPanayi, Adriana C.Conzo, GiovanniPanchal, Hemang B.Cook, Alan D.Panchapakesan, BalajiCook, Alex RichardPandey, AparamitaCook, Alonzo D.Pandhi, AbhiCook, SarahPandit, HarshulCooke Bailey, Jessica N.Pandit, Jaideep J.Coombes, BrookePandolfi, LauraCoombs, Lorinda A.Pandya, AbhilashCooper, Jonathan D.Pane, KatiaCooper, Sara J.Pangestu, MulyotoCooper, Theodore V.Panos, Georgios D.Copland, MhairiPanoutsopoulos, AlexiosCopp, TessaPanova-Noeva, MarinaCoppedè, FabioPantaleo, MariaCoppola, AngeloPantalone, Mattia RusselCoppola, SilviaPantavou, KaterinaCoras, RoxanaPantcheva, Mina B.Corbella, StefanoPanzuto, FrancescoCordano, ChristianPaoletti, GiovanniCordeanu, Elena-MihaelaPaolino, GiovanniCordero, AlbertoPaolucci, TeresaCordido, FernandoPaone, G.Córdova, AlfredoPapa, MicheleCorley, MichaelPapa, RiccardoCormio, GennaroPapadakis, ZachariasCornara, StefanoPapadia, AndreaCorneau, AurelienPapadimitriou, LamprosCornelissen-Guillaume, GermainePapadopoulos, Christodoulos E.Cornelisz, IljaPapadopoulos, Konstantinos S.Cornish, ElisaPapadopoulou, SousanaCorominas Roso, MargaritaPapageorgiou, Kostas A.Corradetti, ValeriaPapaharilaou, YannisCorradi, AnnaPapaioannou, Vasilios E.Corrado, GiovanniPapaioannou, WilliamCorrao, SalvatorrePapakonstantinou, Panteleimon E.Correale, MichelePapaleo, FrancescoCorris, Paul A.Papalia, GiuseppeCorsalini, MassimoPapalia, RoccoCortés, Kamila CaraballoPapalois, VassiliosCortes, RaquelPapamichael, KonstantinosCortez, M. M.Papamichail, KonstantinosCorti, ChiaraPapamitsou, TheodoraCortis, CristinaPapanas, NikolaosCortright, Ronald N.Papanna, RameshaCorver, Wim E.Papazafiropoulou, AthanasiaCosci, IlariaPapazisis, GeorgiosCosentino, Nicola M.Pape, Hans ChristophCosenza, Lucia CarmelaPapi, PieroCosmi, EricPapi, RiginiCosmi, ErichPapiris, Spyros A.Cossin, MarcoPaplińska-Goryca, MagdalenaCossins, JudyPapoutsoglou, PanagiotisCosta, Francisco MoscosoPaprocka, JustynaCosta, PeterPaquette, MaxCosta, RaquelParajuli, NirmalaCostalonga, MassimoParamythiotou, ElizabethCostamagna, GuidoParás-Bravo, PaulaCostan, Victor-VladParashar, BhupeshCostantini, LaraParaskevaidi, MariaCostanza, AlessandraParaskevas, George P.Costanzo, AntonioParatz, JenniferCostanzo, LucaPardi, NorbertCosta-Pinto, Ana RitaParent, RomainCotton, MichaelParente, AlessandroCottreau, Jessica M.Parente, PaolaCottrell, Elizabeth C.Parenti, IlariaCouch, James R.Parfrey, HelenCoufal, NicolePariente, GaliCoughlan, GillianParinaud, JeanCougnoux, AntonyParis-Alemany, AlbaCoulson-Thomas, VivienPark, AelyCoumans, Joëlle V. F.Park, Ah-MeeCouper, KeithPark, Chan KeeCoupland, Lucy A.Park, Chang MinCourtoy, Guillaume E.Park, DonghwiCouturier, YvesPark, EujinCovani, UgoPARK, Eun JeongCovassin, NaimaPark, Eun-YoungCovelli, EdoardoPark, Hae RyounCovino, MarcelloPark, Hae-SimCowie, RaelenePark, HoonCoyle, Patricia K.Park, Hye YunCozar, I.Park, Jae-HyeongCozma, DragosPark, Jae-HyungCozzani, EmanuelePark, JaesikCraig, Jennifer PPark, Ji-ManCrane, Isabel J.Park, Ji-woonCravero, Joseph P.Park, JohnCrawford, SeanPark, Jun YongCrehuá-Gaudiza, ElenaPark, Ki HoCremer, MarionPark, Ki-cheongCrenn, Pascal P.Park, KihanCreuzenet, CarolePark, Kyung WooCrews, FultonPark, Sae WoongCriado, BegoñaPark, Sung-HwanCrimi, ClaudiaPark, Yong-HoCrincoli, VitoPark, Young SeoCripps, MichaelPark, Young-GukCrisafulli, GiovanniPark, Youn-SooCrismale, James F.Parker, ChristinaCristiani, Costanza MariaParker, JackCristofaro, Maria GiuliaParker, M.Cristoferi, LauraParker, William A. E.Croagh, DanielParnes, Lorne S.Croce’, Lory SaveriaParnetti, LucillaCrocini, ClaudiaParnianpour, MohamadCrofts, CatherineParodi, AuroraCrohn, Helen M.Parodi, GuidoCron, Randy Q.Parodis, IoannisCrook, Amy E.Parra-Membríves, PabloCrosbie, EmmaParrinello, GiampieroCrosby, D. A.Parrington, JohnCross, MichaelParrini, SimoneCrosson, JasonParsanathan, RajeshCrouser, ElliottPärsson, HåkanCrowe, KellyPartido, Brian B.Crowle, CathrynPartsch, HugoCruickshank-Quinn, CharmionPascal, PomièsCrus-Topete, DianaPascale, Marco MariaCruz Diaz, DavidPaschall, Mallie J.Cruz, FranciscoPaschos, Nikolaos K.Cruz, Milagros Hidalgo De LaPasciu, ValeriaCsernok, ElenaPascoal, Augusto GilCubellis, MariaPascual-Leone, AntonioCuenca-Barrales, CarlosPasek, MałgorzataCuesta Gómez, AliciaPashikanti, SrinathCui, YingPasquie, Jean-LucCuker, AdamPasquini, LuciaCumbo, CosimoPasta, GianluigiCunningham, LisaPasternak, AlexanderCunningham, PatrickPastor, JesúsCunnington, Aubrey J.Pastore, IdaCuppari, LeaPastori, DanieleCurci, ClaudiaPastuszka, AgnieszkaCurcio, Christine A.Pataka, AthanasiaCurry, ScottPatarrão, RitaCurtis, Joseph E.Patatanian, EdnaCurtis, Michael J.Patel, Ankit B.Curtis, Susanna A.Patel, Daxesh P.Curzen, NickPatel, Hiren C.Cushing, SharonPatel, NibeditaCusick, Marianne V.Patel, NiketaCusimano, Robert JamesPatel, Nimesh A.Cutler, Christopher W.Patel, SamarthCuvillon, PhilippePatel, Tapan P.Cuypers, BartPatel, UrvishCwiklinska, AgnieszkaPatelis, NikolaosCytlak, UrszulaPatella, VincenzoCzarnecka, AnnaPaterakis, Konstantinos N.Czarnecki, PeterPaternoster, GianlucaCzepiel, JacekPathak, DhrubaCzernochowski, DanielaPati, DipaCzesnik, DirkPatini, RomeoCzigany, ZoltanPatnaik, Sourav S.Czyżewski, KrzysztofPatolia, SetuD’Alterio, Maurizio NicolaPatricia, Concheiro-MoscosoD’Ambrosi, NadiaPatrone, CesareD’Amico, CesarePatruno, CataldoD’Angeli, FlorianaPatrussi, LauraD’Angelo, ChiaraPatterson, TiffanyD’Apuzzo, FabriziaPaudel, Keshav RajD’Ascenzo, FabrizioPaul, Brandon TylerDa Cruz, LyndonPaul, NicolasDa Rocha Lopes, LuisPaul, PercoDa´vila, IgnacioPaul, TimirDabizzi, EmanuelePaul, VisheshDa̧browska-Galas, MagdalenaPaul, YaswenDąbrowski, MariuszPaulik, EditDąbrowski, WojciechPaulino, JorgeDaca, AgnieszkaPaulo, SiriDadras, MehranPaulson, Olaf B.Dafer, Rima M.Paulus, Yannis M.D'Aguanno, SimonaPausch, Thomas.Dahlmann-Noor, AnnegretPavasini, RitaDai, YaoPavkov, Meda E.Daïen, ClairePavlovic, NatasaDaifi, ChantalePavone, Mary EllenDal Ben, MatteoPavone, Mary Ellen GDalbeni, AndreaPavone, VitoDalgaard, FrederikPawlaczyk-Kamieńska, TamaraDalin, Martin G.Pawlak, AgnieszkaDalinghaus, MichielPawlak, JoannaDalkiz, MehmetPawlak, KatarzynaDalkner, NinaPawlik, AndrzejDalla Palma, BenedettaPawlitzki, MarcDall'Acqua, StefanoPawlowska, ElzbietaDalm, VirgilPayan-Carreira, RitaDalmases, MireiaPayer, JurajDalton, BethanPeacock, ChristopherDam, GittePearman, Timothy P.Dambaeva, SvetlanaPearson, Claire F.D'Ambrosi, RiccardoPearson, JamesDamian, DionaPeart, JasonDamiani, Gianluca R.Pecci Lloret, María PilarDamiani, Gianluca RaffaelloPecci-Lloret, Miguel R.Damiani, GiovanniPéchiné, SéverineDammacco, RosannaPecker, Lydia H.Dampier, CarltonPeckham, DanielDamsgaard, Else MariePecks, UlrichDamsky, William E.Pecoraro, AngelaDamulevičienė, GytėPedano, Mariano N. SimónDancause, K. N.Peddi, V. RamDand, NickPedemonte-Sarrias, EduardDandoy, ChristopherPedro-Botet, JuanDaneshzand, MohammadPedrotti, EmilioDanesino, CesarePedullà, EugenioDanforth, Teresa L.Peeples, EricDangayach, NehaPeham, JohannesD'Angelo, ARMANDOPeinemann, FrankD'Angelo, StefaniaPeixoto, Maria ManuelaDanias, PeterPejoski, DavidDaniel Israeli-Korn, SimonPeláez, JesúsDaniel, Morales CanoPeles, EinatDanielewicz, AnnaPelin, MarcoDaniels, M. Leigh AnnePelizzari, GiacomoDanks, MarcellaPellat, AnnaDannenberg, LisaPellegrino, GerardoDanson, Edward James FrazerPelliccioni, Gian AndreaD'Antò, VincenzoPelliccioni, PaoloDapunt, UlrikePels, AnoukD'Apuzzo, FabriziaPeltokangas, MikkoDar, Wasim A.Peluso, LorenzoDarmochwał-Kolarz, DorotaPemp, BertholdD'Arrigo, SoniaPendurti, GopichandDarrow, JonathanPeng, ChuanqiDarvizeh, AtanazPeng, DunfaDas, AnshumanPeng, Xianlu LauraDas, ArabindaPeng, YangDas, BibhutiPeng, Yi-JenDas, FalguniPengo, MartinoDas, NupurPenk, JamieDas, RajdeepPenmetsa, Gopi K.Das, RupaliPenna, FabioDas, SumitPenson, PeterDash, Ranjeet PrasadPentaris, PanagiotisDaskalakis, KosmasPepe, FrancoDassios, TheodorePepe, JessicaDastsooz, HassanPeral, AssumptaDatta, RupsaPeraldo-Neia, CaterinaDatta, SiddharthaPeran, IvanaDattola, AnnunziataPerdigoto, David NoivaDaub, SteffenPerdomo, JoseDauby, NicolasPeregud-Pogorzelska, MałgorzataDaunys, GintautasPereira, Ana De FátimaDauwe, DieterPereira, SofiaDavaris, NikolaosPereira, Sofia S.Daveluy, StevenPereira-da-Silva, LuísDavid Cordeiro, SousaPereiro, Arturo X.David, MatthiasPeretti, SaraDavid, Vlad LaurentiuPérez García, Víctor M.Davideau, Jean-LucPérez Prieto, DanielDavies, BenjaminPérez, Felipe OjedaDavis, Timothy M. E.Perez, KimberlyDavoodi-Bojd, EsmaeilPérez, PalomaDavos, ConstantinosPérez-Alba, AlejandroDavoudi, AnisPérez-Caballero, LauraDawra, RajinderPerez-Cabezas, VeronicaDay, Andrew S.Pérez-Calvo, Juan I.Dayal, SanjanaPérez-Castrillón, Jose LuisD'Azzo, AlessandraPerez-Farinos, NapoleonDe Agostini, ArianePérez-Fuentes, María Del CarmenDe Alcantara Camejo, FlavioPérez-Herrero, María A.De Amorim Almeida, HenriquePérez-Ordás, RaquelDe Angelis, BarbaraPerez-Perez, Guillermo IgnacioDe Angelis, NicolaPérez-Ros, PilarDe Angelis, PaolaPerez-Sousa, Miguel A.De Angelis, SaraPeriasamy, RameshDe Backer, GuyPerisetti, AbhilashDe Berardis, DomenicoPerkisas, StanyDe Biase, AlbertoPerlman-Emodi, AlonaDe Boer, AnneloesPero, RaffaelaDe Bruijn, Henriette S.Perretta, Julianne S.De Candia, EricaPerrin, ThomasDe Carvalho, MamedePerrone, Marco AlfonsoDe Ciuceis, CarolinaPerrotta, FabioDe Cock, DiederikPersampieri, SimoneDe Coninck, VincentPersano, StefanoDe Donato, GianmarcoPesce, FrancescoDe Falco, ValentinaPesonen, Eero JuhaniDe Francesco, FrancescoPetcherski, AntonDe Francesco, VincenzoPetelczyc, KrzysztofDe Giorgi, AlfredoPeter, KarlheinzDe Giorgio, RobertoPeterli, RalphDe Gracia, PabloPeters, StefanDe Groef, AnPetersen, EskildDe Gruijl, FrankPetersen, SvenDe Herder, Wouter W.Peterson-Hiller, AmieDe Hollanda, AnaPetiet, AlexandraDe Jong, Pim A.Petito, AnnamariaDe Kort, ElizabethPetito, ValentinaDe La Calle, María I.Petkow-Dimitrow, Pawełde la Torre, Juan C.Petraglia, FeliceDe Lago, EvaPetrakis, Ismene LeonidaDe Lange, JanPetreaca, Ruben C.De Leo, AntonioPetro, Thomas M.De Ligt, KellyPetrosillo, NicolaDe Martin, SaraPetrovski, GoranDe Meritens, Alexandre BuckleyPetta, IoannaDe Miguel-Pérez, DiegoPettorruso, MauroDe Montalembert, MarianePeyronnet, DamienDe Morais, Sharon D.Peyrony, OlivierDe Mulder, MiguelPezone, AntonioDe Nucci, GermanaPezzuto, FedericaDe Oliveira Silva, DaniloPfaff, JohannesDe Paco Matallana, KatyPfeifer, AleksandraDe Paiva, Cintia S.Pfeuffer, SteffenDe Palma, ClaraPham, KienDe Palma, GiuseppePhan, JenniferDe Pasquale, ValeriaPhan, TungDe Pastena, MatteoPhelps, Kenneth R.De Paz Fernández, Jose AntonioPhilippon, Marc J.De Petro, GiuseppinaPhilipsen, RalfDe Prado, Armando PerezPhillips, ChristianDe Rasmo, DomenicoPhilpott, Carl M.De Re, VallìPhu, JackDe Reijke, T. M.Phulera, SwastikDe Rienzo, AssuntaPiacenti, IlariaDe Roo, ChloePiaggesi, AlbertoDe Roock, SophiePiana, SimonettaDe Roock, SytzePiancino, MariaDe Rooij, DirkPiasecka, MałgorzataDe Rooij, Myrna M. T.Piasecki, JessicaDe Sanctis, GiovannettiPiatelli, GianlucaDe Santis, DanielePiatt, Jennifer A.De Santo, CarmelaPicard-Hagen, NicoleDe Sio, MarcoPicardi, AlessandraDe Valk, H.w.Picchianti-Diamanti, AndreaDe Vasconcellos, Adalberto BastosPiccin, AndreaDe Vito, DanilaPiccinin, ElenaDe Vos, JohnPiccinini, HelenaDe Vries, Adrianus J.Piccirillo, RosannaDe Vroege, LarsPiccoli, GiorginaDe Waard, VivianPiccoli, Giorgina BarbaraDe Wilde, Lieven F.Picconi, FabianaDe Wilde, Rudy LeonPicelli, AlessandroDe Zinis, Luca Oscar RedaelliPichon, MaximeDean, JamiePickering, RaeleneDean, Matthew J.Piegeler, TobiasDean, Natalie E.Piemontese, SimonaDearborn, Jennifer LPienkowski, MartinDębska-Ślizień, AlicjaPieralli, FilippoDecaro, NicolaPieralli, StefanoDecarolis, BorisPierfrancesco Francesco, BassiDecker, Brooke K.Pierre, Joseph M.Deckers, Jaap W.Pietiainen, MillaDecuypere, Jean-PaulPietrangelo, TizianaDeda, OlgaPietrasanta, CarloDedy, Nicolas J.Pietropaoli, DavideDeeb, Janina GolobPietzner, MaikDeel, MichaelPiffaretti, GabrieleDeelman, L. E.Pignata, ClaudioDeemer, SarahPignataro, GiuseppeDeery, ChrisPihlblad, Matthew S.DeFelice, NicholasPihut, MalgorzataDeFilippis, Andrew PaulPihut, MałgorzataDeflandre, EricPijls, BartDegan, DianaPikuleva., IrinaDeie, MasatakaPilato, FabioDekhuijzen, RichardPilegaard, Hans KristianDel Ben, MariaPillet, SylvieDel Casale, AntonioPilling, DarrellDel Pinto, RitaPilloud, Marin A.Del Rio, MaguyPilonis, NastazjaDel Rosal, TeresaPilutin, AnnaDel Turco, SerenaPilvankar, MinuDelafield-Butt, JonathanPina, AnaDeLeon-Pennell, Kristine Y.Pinart, ElisabethDelgado Ruiz, RafaelPinazo Duran, Maria DoloresDelgado, LuísPini, Luigi AlbertoDelhem, NadiraPinto, AntonioDeligeoroglou, Efthimios K.Pinto, AshwinD'Elios, Mario MilcoPinto, EleonoraDelitala, MarcelloPinto, José AntonioDeliyanti, DevyPiotin, MichelDella Pepa, GiuseppePiotrowski, DamianDella Rocca, Domenico Giovanni.Piotrowski, WojciechDella Torre, EmanuelPiper, BrianDellinger, E. PatchenPirani, VittorioDelpón, EvaPiras, AlessandroDelprat, BenjaminPirenne, JacquesDelsante, MarcoPiret, JocelyneDemaria, OlivierPirklbauer, MarkusDeme, PragneyaPiroth, LionelDemer, Joseph L.Pirotta, StephanieDemetter, PieterPisaneschi, FedericaDeml, Moritz C.Pisani, Didier F.Demnitz, NaiaraPisanu, ClaudiaDen Uil, CortisaanPisapia, PasqualeDeng, LuPiscione, FedericoDeng, YoupingPishnamaz, MiguelDengler, FranziskaPiskin, SenolDenis, FredericPiszczatowski, SzczepanDenis, KathleenPitchika, VinayDennett, AmyPitt, MirandaDennett, ElizabethPitynski, KazimierzDenning, DavidPiver, EricDennison, Elaine M.Piwocka, KatarzynaDenu, Ryan A.Plachokova, Adelina S.Deraison, CélinePlakht, YgalDerakhshan, MohammadPlanck, TerezaDereke, JonatanPlante, Timothy B.Derfler, KurtPlanté-Bordeneuve, ViolaineDerman, BenjaminPlaudis, HaraldsDermitzakis, Emmanouil V.Playford, Martin P.Derrick-Roberts, Ainslie Lauren K.Plaza, GuillermoDeruelle, PhilippePlebani, MarioDervenis, PanagiotisPletsch-Borba, LauraDerwall, MatthiasPlichta, MartaDesai, AmritaPlo, IsabelleDesando, GiovannaPluta, JanDesantis, VanessaPochet, RolandDeshmukh, Umesh S.Podbielska, HalinaDesideri, LorenzoPoddighe, DimitriDeSouza, Danielle D.Podestà, Manuel AlfredoDespa, FlorinPodfigurna, AgnieszkaDesprez, CharlottePoggio, PaoloDessau, RamPoghosyan, TigranDessein, Patrick H.Pohl, JuliaDestrempes, FrançoisPohlemann, TimDesyatova, AnastasiaPoitrasson-Rivière, AlexisDetry, OlivierPojskić, MirzaDetti, LauraPokorska-Śpiewak, MariaDeUgarte, Daniel A.Polańczyk, AndrzejDeutsch, CorneliaPolano, MaurizioDevaraj, AishwaryaPolanska, AdrianaDevarakonda, SrinivasPoldermans, DonDevarshi, Prasad P.Pole, NnamdiDeveaux, EtiennePolese, LinoDevenney, Lydia E.Polewczyk, AnnaDevesa, JesúsPolglase, GraemeDevlin, HughPoli, GuiseppeDevy, ZismanPolich, GingerDeWald, TracyPöling, JochenDewane, Michael PPolis, BaruhDewey, Rebecca SusanPoliti, Letterio S.Dey, Amit KumarPolito, Anna NunziaDeyu, HuPolizzi, AlessandroDhaenens, LienPollari, FrancescoDhanasekaran, MuraliPoloni, Tino EmanueleDharker, NachiketPolovina, Snezana P.Dharmaraj, RajmohanPolte, Christian LarsDhawan, AndrewPolverino, FrancescaDhillo, WaljitPolyakova, MarynaDhiman, KunalPolzin, AminDhir, ShaliniPommer, BernhardDhooge, Ingeborg Johanna MariaPommier, Rodney F.Di Baldassarre, AngelaPonchel, FrédériqueDi Bella, GianlucaPongratz, GeorgDi Blasio, AlbertoPonikau, Jens U.Di Caprio, GiuseppePons, RoserDi Carlo, MarcoPontejo, Sergio M.Di Cesare, ErnestoPontoriero, AntonioDi Cristofori, AndreaPontrelli, PaolaDi Domizio, JeremyPoole, DavidDi Gennaro, FrancescoPoonia, BhawnaDi Giacomo, RobertaPop, RaoulDi Gianfilippo, RiccardoPop, Tudor LucianDi Giuseppe, DanielaPop, VictorDi Laura, AnnaPope, Daniel H.Di Leo, AlfredoPopławski, PiotrDi Leo, GraziaPopma, JeffreyDi Maio, PasqualePopov, DoinaDi Mario, CarloPopow, MichałDi Martino, OrsolaPoppe, MichaelDi Mascio, DanielePorcheri, CristinaDi Mauro, MichelePorela, PekkaDi Micco, PierpaoloPorpora, Maria GraziaDi Muzio, AntonioPorreca, AngeloDi Nardo, DarioPorritt, JennyDi Nicola, MarcoPortella, AndreDi Paolo, VirginiaPorten, Sima P.Di Pietro, PaolaPortillo, María P.Di Pietro, PietroPortnoy, SigalDi Pino, AntoninoPosada-Quintero, Hugo F.Di Saverio, SalomonePosadas, TomasDi Scioscio, ValerioPosadzy-Malaczynska, AnnaDi Sebastiano, PierluigiPosarelli, ChiaraDi Spiezio Sardo, AttilioPoškus, EligijusDi Tommaso, LuigiPosluszny, Joseph A.Di Zazzo, AntonioPospichalova, VendulaDiab, AdiPostler, Thomas S.Diab, RamiPota, VincenzoDiacinti, DanielePotaczek, Daniel P.Diamantidis, Michael D.Potes, YaizaDiamond, David M.Pott, AlexanderDiane Valeriano, ValeriePottel, HansDias-Ferreira, JoãoPotukuchi, Praveen KumarDiaz Balzani, Lorenzo AlirioPouga, LydiaDiaz, Jose AntonioPoulsen, DavidDiaz-Delcastillo, MartaPountos, IppokratisDiaz-Gomez, Jose LuisPourafshar, ShirinDiaz-Gonzalez, FedericoPoureslami, IrajDiaz-Menendez, MartaPourhassan, MaryamDiBardino, DavidPourié, GrégoryDichtl, WolfgangPourkazemi, FereshtehDickman, RamPourmalek, FarshadDidkowska, JoannaPovoa, PedroDiDonato, Joseph A.Powell, Timothy R.Didonna, AlessandroPower, Dominic MichaelDiederichsen, Louise PyndtPozzato, GabrieleDiedrich, StephanPozzi, AndreaDiel, RolandPozzo, FedericoDiepers, MichaelPrabhu, LakshmiDierckx, TimPradère, BenjaminDieterich, LotharPradhan, DineshDigby, Geneviève C.Pramudita, Jonas A.Dijkstra, JoukePranno, NicolaDik, Willem A.Prasad, RamDika, EmiPrasadh, SomasundaramDikalova, Anna E.Prasitlumkum, NarutDillon, JosephPrat, G.Dillon, Laura W.Prati, CarloDiluvio, GiuliaPrats, Raquel GuillamatDimitri, Giovanna MariaPravettoni, GabriellaDimitriadis, KyriakosPreckel, BenediktDimitroglou, YannisPreininger, BerndDimitropoulos, KonstantinosPrell, TinoDimitroulas, TheodorosPrella, MauraDinas, KonstantinosPresbitero, PatriziaDini, PouyaPrescott, AlanDini, ValentinaPressney, IanDiniz-Freitas, MarcioPreston, CatherineDinkler, LisaPreston, NickDiogo, PatriciaPreston-Campbell, RebeccaDiogo, PatríciaPrete, AlessandroDionysopoulos, DimitriosPrete, MarcellaDisanto, GiulioPreti, GiulioDiserens, KarinPreziosa, PaoloDitsios, KonstantinosPriesemann, ViolaDivaris, KimonPriet, StéphaneDivella, RosaPrimack, WilliamDivine, George W.Primack, William A.Divino-Filho, JosèPrimignani, MassimoDixon, AndrewPrince, MilesDjerada, ZoubirPrincipi, MariabeatriceDjordjevic, IlijaPrins, Maarten R.Djordjevic, JelenaPriola, Stefano MariaD'Lima, DarylPrior, AndersDobrosielski, Devon A.Pritchard, NatashaDobruch, JakubPrivratsky, JamieDobrzański, Leszek A.Probst, MonikaDocampo, A.Probst, PascalDocea, AncaProcopeţ, BogdanDocherty, Kieran F.Prodam, FlaviaDocherty, SharonProdinger, PeterDodd, HelenProdinger, Peter M.Dodd, Rebecca D.Proesmans, MarijkeDoddapattar, PrakashProfirovic, JasminaDodoo, ChristopherProkesch, Bonnie C.Doenyas-Barak, KerenProtheroe, AndrewDoerr, Johanna M.Protonotarios, AlexandrosDoherty, KathleenProvan, Sella A.Dohi, TomotakaProvini, FedericaDohrn, Ing-MariPruenster, MonikaDoi, KentPruett, Brandon S.Doizi, SteevePrystupa, AndrzejDoloff, Joshua C.Prytherch, DavidDolscheid-Pommerich, RamonaPrzekoracka-Krawczyk, AnnaDom, GeertPrzemysław, WielgatDombrowski, TobiasPsarra, Anna-Maria G.Dominguez-Maldonado, GabrielPsarras, SteliosDominguez-Vicent, AlbertoPsarris, AlexandrosDominika, StygarPsychogios, GeorgiosDonadio, CarloPtaszkowski, KubaDonadon, MatteoPtaszynska-Kopczynska, KatarzynaDonahue, PaulaPucci, GiacomoDonal, ErwanPucci, ResiDonat, Francisco Javier SilvestrePuchades, María JesúsDonati, MarcelloPuddu, Paolo EmilioDonato, LuigiPugazhenthi, SubbiahDonders, GilbertPugliese, NicolaDondi, GiuliaPugliese, Nicola RiccardoDondossola, DanielePuhka, MaijaDondzilo, LauraPujol-Borrell, RicardoDonevant, Sara BellePuła, BartoszDong, FengpingPulcinelli, Fabio M.Dong, Huan-JiPulido, RafaelDong, LihuaPulito, ClaudioDong, QinglinPullarkat, VinodDong, WeiPulle, Ranjeev ChrysanthDonker, TaraPulliero, AlessandraDonnez, JacquesPulsatelli, LiaD'Onofrio, LucaPuppo, FrancescaDonohue, TerrencePuranik, Chaitanya P.Donovan, LukePuri, KritiDöppner, Thorsten R.Puri, SonamDorado, PedroPurroy, FranciscoDordevic, MilosPurrucker, Jan ChristophDória, SofiaPurudappa, Prabhudev PrasadDöring, NicolaPusateri, AlessandroDörr, MarcusPuskarich, Michael A.Dorst, JohannesPuślecki, MateuszDos Santos, João Miguel MarquesPussinen, PirkkoDosdall, Derek J.Puthia, ManojDoshi, AnishaPutot, AlainDosiou, ChrysoulaPuzo, JoseDosoky, NouraPyaram, KalyaniDott, Giovanni MarascoPycha, ArminDotzenrath, CorneliaPyke, Robert E.Douglass, JanPyne, John H.Doulamis, IliasPyott, S. J. (Sonja)Doulberis, MichaelPyxaras, StylianosDouris, PeterPyza, ElżbietaDovnik, AndrazQanud, KhaledDowell, Richard C.Qasim, MuhammadDowling, Ryan J. O.Qasim, Syed Saad B.Doyen, DenisQayed, EmadDoyle, Tracy J.Qi, Yanfei (Jacob)Dozio, ElenaQian, FengDragonieri, SilvanoQiao, AijunDrakoulis, NikolaosQin, HuaDream, SophieQin, LuyeDreesen, ErwinQin, Ren YiDreger, HenrykQiu, ZhenDreier, Jens P.Qu, NingDreimann, MarcQuaglino, DanielaDreumont, NatachaQuallich, SusanneDrici, Milou-DanielQuenby, SiobhanDrieu, AntoineQuero, GiuseppeDriscoll, Marcia S.Quigley, RaymondDriul, LorenzaQuinn, Peter M. J.Drosos, Alexandros A.Quinquenel, AnneDrougard, AnneQuintero, Rubén A.Dryden, Matthew S.Quoc Le, Trung (Tim)Drygas, WojciechQuock, RyanDu, YixingQureshi, Waqar A.Duarte, LilianneR. Marx, GeraldDubé, CatherineR. Naik, AkshataDube, PrabhatchandraRaake, Philip W. J.Dubinski, DanielRabant, MarionDubis, Adam M.Rabbat, MarkDubousset, JeanRabec, ClaudioDuca, FranzRaber-Durlacher, Judith E.Duca, YleniaRabiee, AtefehDucarme, GuillaumeRabionet, Silvia E.Duchi, SerenaRabitti, StefanoDuchnowski, PiotrRabkin, Simon W.DuCoin, ChristopherRabuazzo, Agata MariaDuda, MałgorzataRadbruch, AlexanderDudas, BertalanRader, FlorianDuda-Sobczak, AnnaRadhakrishna, GaneshDude, CarolynnRadovanovic, DejanDudek, WojciechRadwan, PawełDudman, SusanneRadwanski, Ryan E.Dudzińska, EwaRafei, MoutihDudzinski, David M.Raffa, GiovanniDuerksen-Hughes, PenelopeRaffa, SalvatoreDuffell, LynseyRafferty, GerrardDuffy, DarraghRaffetto, JosephDuffy, TomasRafikov, RuslanDufour, Jannette M.Ragavan, MukundanDugue, BenoitRaggi, ChiaraDuijnhouwer, ToonRagia, GeorgiaDuitman, JanWillemRagni, EnricoDuits, AnnelienRago, DanielaDuke, JJRago, RoccoDullenkopf, AlexanderRagonese, MauroDuma, Virgil-FlorinRagusa, AntonioDumais, AlexandreRahib, LolaDumic, IgorRahkola-Soisalo, PäiviDumnicka, PaulinaRahman, AzizDumont, Aaron S.Rahmanuddin, SyedDuncan, SteveRai, Nayanjot K.Duncker, DavidRai, TatemitsuDunlop, DouglasRai, VikrantDunne, MargaretRaikes, Adam C.Duntas, LeonidasRaimondi, LauraDuong-Quy, SyRaimundo, Armando Manuel De MendonçaDupieux, CélineRaimundo, MiguelDupuis-Girod, SophieRaimundo, NunoDuque, PatriciaRaina, RupeshDuquette, PierreRainero, InnocenzoDurán, Manuel NovalesRaja, RajikhaDuran, Sue HudsonRajagopalan, AnugrahaDurand, RobertRajakumar, ChandrewDurand-Moreau, QuentinRajamannan, NaliniDuraturo, FrancescaRajoli, Rajith K. R.Durlik, MagdalenaRajpert-De Meyts, EwaDurrieu, FrancoiseRaju, NagarajanD'Ursi, Anna MariaRaju, SnehaD'Urso, GildaRakow, AnastasiaDuryea, ElaineRakowski, TomaszDuschek, StefanRali, Aniket S.Dusetti, NelsonRali, ParthDusi, VeronicaRall, Katharina K.Dusso, AdrianaRalli, MassimoDutta, PranabanandaRallidis, Loukianos S.Duvekot, J. J.Ramalho-Santos, JoãoDuverger, OlivierRamamurthy, ChethanDuzgunes, NejatRamasamy, Prem K.Dwivedi, GarimaRamchandani, DivyaDykun, IrynaRameckers, Eugene A.Dylke, Elizabeth S.Ramel, SaraDymarek, RobertRamiasa, MelanieDyment, D. A.Ramieri, GuglielmoDysli, ChantalRamírez Sánchez, ManuelDziedzic, RobertRamírez, JuliaDziegiel, PiotrRamirez, PedroDziewięcka, EwaRamírez-Vélez, RobinsonDziodzio, TomaszRamiro-Cortijo, DavidDziurkowska, EwelinaRamkhelawon, BhamaEa, Hang-KorngRamnath, RainaEarle, Kenneth A.Ramos Martinez, AntonioEason, JamesRamos, ErivanEastman, Creswell J.Ramos-Garcia, PabloEaton, WilliamRamos-Morcillo, Antonio JesúsEbenbichler, GeroldRamsauer, BabettEblimit, AidenRamsay, Michael A.Ebmeier, KlausRamsay, Michael A. E.Ebner, FlorianRanebo, Mats C.Ebneter, AndreasRanganathan, ShivakumarEboigbodin, KevinRangé, HélèneEbraheim, NabilRanjan, AmareshEbrahimi, DiakoRanjan, KishuEccles, JessicaRanjbar, MahdyEck, Ruben J.Ranstam, JonasEdamakanti, ChandrakanthRanucci, MarcoEddy, Brandon P.Rao, ChalapatiEddy, Kamryn T.Rao, NarsingEdeline, JulienRao, PrethyEdman, MariaRao, ShiwangniEdmonds, MichaelRapacciuolo, AntonioEdwards, GeneaRapi, StefanoEdwards, HelenRapone, BiagioEells, JanisRaschi, EmanuelEfird, Jimmy T.Rashad, AhmadEfrati, YanivRashid, Mohammad HarunEftekhari, AshkanRashid, Naim U.Efthymiou, StephanieRaskov, Hans HenrikEgerman, RobertRasmussen, AndersEgert, MarkusRasmussen, StenEggleston, JeffreyRasmussen, Torben RiisEhler, JohannesRasouli, NedaEhlken, ChristophRasouli, OmidEhrhardt, HaraldRasova, KamilaEhrlich, StefanRassaf, TienushEiber, CalvinRassekh, Christopher H.Eichelmann, Ann-KathrinRassekh, Shahrad RodEichstaedt, ChristinaRatajczak, MonikaEick, SigrunRather, GulamEid, Albert J.Rathinasabapathy, AnandharajanEijnde, Bert OP ’TRätsep, MatthewEikermann, MatthiasRattray, ZahraEiros, RocioRauch, BernhardEisenberg, LaurieRauch, GerdEisendle, KlausRauner, MartinaEisendrath, Stuart J.Ravassa, SusanaEisenga, Michele F.Ravidá, AndreaEitaro, KodaniRavin, James G.Ejaz, AsimRavindra, VijayEjima, KeisukeRawicz-Pruszyński, KarolEkberg, OlleRawla, PrashanthEkeløf Busch, Sarah VictoriaRawlings, Stephen A.Ekstrand, Chelsea L.Ray, SamriddhaEl Hadi, HamzaRayar, MichelElahi, FazleRay-Barruel, GillianElaswad, AhmedRaychev, RadoslavElbadawi, AymanRaz, GalElce, AusiliaRazim, AgnieszkaEldeiry, Leslie S.Re, RebeccaEleftheriadis, TheodorosRead, Jonathan M.Elessawy, MohamedReame, Nancy KingElfadawy, NissreenReams, RomoniaElfvin, AndersReardon, MichaelEl-Gazzar, Mohamed M.Rebelos, EleniElgzyri, TargRebmann, VeraElhai, MurielReca, Octavio LuqueEligar, Vinay S.Recalde, SergioElinder, Carl GustafRechberger, TomaszElis, SebastienRecio, RaúlEljaafari, AssiaRecuero, SandraElkana, OdeliaReczuch, Krzysztof W.Ellinger, IsabellaRedd, AndrewElliott, Jessie A.Reddy, Jay P.Ellis, JanetReddy, KesavaEllis, MatthewReddy, Sudhir PuttyElmarakby, AhmedReddy, YogeshElterman, DeanReed, NicholasElvira-Torales, Laura InésReed, William R.Elyaman, WassimReeder, ScottElzbieta, SmolewskaReers, StefanEmamifar, AmirRees, SharonEmerald, Mila A.Refaai, Majed A.Emmanuelle Le, BarsRefetoff, SamuelEmmerich, Karl-HeinzRega, DanielaEmmert, HilaRegal, JeanEmmett, LouiseRegan, BrianEmoto, KatsuraRegan, ChristopherEmrich, InsaRegazzo, DanielaEmtestam, LennartRegenhardt, Robert W.Enache, DanielaRegis, ClaudiaEnderling, HeikoRegunath, HariharanEndo, AkiraRehman, MichaelEndo, HidekiRehman, ShehzadENDO, KazuhikoReich, David L.Eng, TonyReichenbach, Zachary WilmerEngelhard, Herbert H.Reicher, SofiyaEngelhardt, MonikaReider, Lisa M.Engelhardt, Paul FriedrichReidy, Paul T.Enghard, PhilippReikvam, HåkonEngland, AndrewReimann, Regina R.Engler, AnnaReina Bueno, MaríaEnko, DietmarReindl, MartinEnomoto, HirayukiReindl, WolfgangEnwemeka, ChukukaReinelt, TilmannEnzmann, FlorianReinisch, AndreasEozenou, CarolineReinstadler, SebastianEpelman, SlavaReischer, TheresaEppard, ElisabethReiser, JochenEramo, StefanoReisfeld, SharonErb, CarlReisman, YacovErbel, RaimundReiss, PhilipErger, FlorianReiter, ThomasEri, RajaramanRekow, E. DianneEriksen, Erik F.Rémi, JanErivan, RogerRemková, AnnaErkmen, Cherie P.Remuzgo-Martínez, SaraErnesto, Cortés-CastellRenda, GiuliaEROVIC, Boban M.Rendos, Nicole KErwich, Jan Jaap H. M.Renew, J. RossEschbach, Daphne-AsimeniaRengo, SandroEscribano, JulioRenkema, Kirsten Y.Escribano-Subias, Pilar M.Rennard, StephenEsfandiarpour, FatemeRenner, WilfriedEskildsen, Simon FristedRenwick, NeilEsler, ColinRenz, NoraEsnaut, StephaneRenzoni, ElizabethEsparham, AnnaRepesse, YohannEspedal, HeidiRepici, MariaelenaEspes, DanielResch, BernhardEspinola-Klein, ChristineResch, ThomasEsposito, CiroReschini, MarcoEsposito, GianlucaReschke, FelixEssid, Sumia MohamedRespino, MatteoEsteban, JaimeRestaino, StefanoEstévez, FranciscoReszec, JoannaEstevez, Jose J.Rethlefsen, Susan AEstrugo-Devesa, AlbertRetter, AndrewEtienne-Julan, MaryseRety, StephaneEttenhofer, Mark L.Reverter, EnricEu, PeterReves, RandallEun Kim, JungReyes, LeticiaEun, Jung WooReznik, SandraEva, WillaertRha, Seung-WoonEvans, Gareth R.Rhee, ChinkookEvans, Holly E.Rhee, HandooEvanson, Nathan K.Rhee, June-WhaEveraert, KarelRhee, Ki-JongExacoustos, CaterinaRhee, TaeminExadaktylos, AristomenisRhein, MathiasExbrayat, Jean-MarieRial Rebullido, TamaraExindari, MariaRibaldone, Davide GiuseppeEzzat, ShereenRibas-Maynou, JordiFabbian, FabioRibeiro, AnaFabbo, AndreaRibeiro, João CarlosFabbrocini, GabriellaRibi, CamilloFacciorusso, AntonioRibom, Eva L.Faciotti, FedericaRicci, Angela DaliaFaconti, LucaRicci, GiuseppeFaggiano, PompilioRicci, Kiersten W.Faggioni, MichelaRicco, Jean-BaptisteFaghihi, DanialRiccò, MatteoFagiano, PompilioRice, CharlesFagoonee, SharmilaRice, JohnFaguer, StanislasRichard, BodnarFahraeus, RobinRichard, BrunoFaict, SylviaRichards, JeremyFaille, DorothéeRichards, Stephen J.Faitot, FrancoisRichter, Claus-PeterFalcone, VeronicaRichter, Jillian R.Falconer, HenrikRichter, KatrinFalsini, BenedettoRichter, KneginjaFalsini, GiovanniRichter, Manuel JonasFalzarano, DarrylRick, Caroline E. M.Fan, Arthur YinRicker, EmilyFancellu, AlessandroRico-Villademoros, FernandoFanciulli, AlessandraRidola, LorenzoFanciulli, GiuseppeRiedel, FabianFang, JenniferRiedel, JensFänge, Agneta MalmgrenRieger, GerulfFansa, HishamRieger, UlrichFara, Gaetano MariaRiegman, Peter H. J.Farahmand, DanRiera, MelchorFaralli, Jennifer A.Riera-Mestre, AntoniFarge, PierreRiess, HannoFarhadfar, NoshaRigante, DonatoFarias, Ana RitaRigatelli, GianlucaFarinas, JérômeRigney, GabrielleFarinha, CláudiaRigoard, PhilippeFarkash, Evan A.Rigon, LauraFarquhar-Smith, W. PaulRigoni, MichelaFarr, SebastianRiis, Jenna L.Farràs-Permanyer, LaiaRikhtegar, FarhadFarre, NuriaRiley, EdwardFarrell, Michael K.Riley, LisaFarrell, Scott F.Rim, John HoonFarronato, DavideRing, AlexanderFarronato, GiampietroRiondino, SilviaFarronato, MarcoRiordan, StephenFasciana, TeresaRios, FranciscoFasola, SalvatoreRipandelli, GuidoFatima, IramRipani, Francesca RomanaFatima, NidaRiquelme, InmaculadaFatone, LauraRisbano, MichaelFattore, LuigiRissiek, BjörnFauquert, Jean-LucRita, Galliera EmanuelaFaurie, BenjaminRiteau, NicolasFaus-Matoses, VicenteRittirsch, DanielFava, GiovanniRiva, AlessandraFava, PaoloRivera, FranciscoFavaloro, Emmanuel J.Rivera, José CarlosFavero, GaiaRivera, VictorFavi, EvaldoRivinius, RasmusFavia, GianfrancoRizk, Elias B.Faviana, PinucciaRizos, EmmanouilFavilli, AlessandroRizvi, ImranFavilli, SilviaRizvi, Syed A. A.Fawzi, AmaniRizzello, FrancescaFebbraio, MarkRizzo, AlessandroFedele, FrancescoRizzo, FabianaFedorowski, ArturRizzo, FrancescaFedosov, SergeyRizzo, GianlucaFeeney, ClaireRizzo, GiovanniFeher, AttilaRizzo, RenataFehlings, MichaelRizzolio, FlavioFeichtinger, MichaelRobbins, LoriFeijóo, Yago LeiraRoberts, AmandaFein, Lydia A.Roberts, C MichaelFeistritzer, Hans JosefRoberts, Drucilla J.Feldman, SteveRoberts, Matthew A.Feldman, Steven R.Roberts, Paul A.Feldo, MarcinRoberts, ThomasFeleus, AnitaRoberts, Wilbur E.Felice, RobertoRobinson, Dale L.Felisati, GiovanniRobinson, PaulFelnhofer, AnnaRobles, AnaFeltgen, NicolasRobles-Marhuenda, A.Feng, Hua-JunRoblin, XavierFeng, YinnianRobson, Matthew JamesFenton, TanisRocca, CarmineFeo, RebeccaRoccella, MicheleFérec, ClaudeRocchi, LorenzoFerens-Sieczkowska, MirosławaRocco, Federico DiFerguson, John ScottRocha, FernandaFerguson, Tanner J.Rochani, AnkitFernandes Fagundes, NathaliaRoche, AidanFernandes, BrunoRoche, FrédéricFernandes, Paulo R. B.Rocheleau, GhislainFernandes, Susan M.Ročka, SauliusFernandez Novell, Josep MariaRocque, Brandon G.Fernández- Santiago, RubénRodbard, Helena W.Fernandez, KatharineRodda, SimoneFernandez, MercedesRoderburg, ChristophFernandez, RosaRodilla, EnriqueFernandez, Teresa TórtolaRodolfo, CarloFernández-Alcántara, ManuelRodrigo, ChamiraFernandez-Botran, RafaelRodrigo, EmilioFernández-Ferro, MartínRodrigo, Juan P.Fernandez-Matias, RubenRodrigo, LuisFernández-Parra, AntonioRodrigues, CarinaFernandez-Peñas, PabloRodrigues, Célia F.Fernández-Tena, AnaRodrigues, Filipe B.Fernandez-Vizarra, ErikaRodrigues, Isabel B.Fernando, WyssRodrigues, PatriciaFernell, ElisabethRodrigues, Robim M.Ferracini, RiccardoRodrigues-Diez, RaulFerraiolo, AntonellaRodríguez García, IsabelFerramosca, AlessandraRodríguez Hermosa, Juan LuisFerrannini, EleRodríguez Mañas, LeocadioFerrantino, LucaRodriguez Sosa, Jose R.Ferrara, GiovannaRodríguez Velasco, Francisco JoséFerrara, PietroRodriguez, AnnaFerrara, ZacharyRodriguez, CelestinoFerraris, ClaudiaRodriguez, Edward K.Ferreira, Flávio P.Rodriguez, Francisca S.Ferreira, Manuel MarquesRodriguez, LuisFerreira-Coimbra, JoaoRodríguez-Almagro, DanielFerreira-Pêgo, CíntiaRodriguez-Calvo, TeresaFerreira-Santos, FernandoRodriguez-Cueto, CarmenFerrer, MiquelRodriguez-Gil, JorgeFerrera, CarlosRodriguez-Gonzalez, MoisesFerrer-García, MartaRodriguez-Llanes, Jose M.Ferrero, SimoneRodríguez-Lozano, Francisco JavierFerretti, GeraldRodriguez-Merchan, Emerito CarlosFerri, FlaminiaRodríguez-Pardo, DolorsFerri, NicolaRodríguez-Vallejo, ManuelFerro, Ana SantosRøe, CecilieFerroni, PatriziaRoeder, FalkFerschl, MarlaRoedl, KevinFesslova, VlastaRoelfing, Jan DuedalFesti, DavideRoeters Van Lennep, JeanineFialka, ChristianRogawski, Elizabeth T.Fiamoncini, JarleiRoger, Simon D.Ficca, GianlucaRogers, Laura Q.Fichna, MartaRogers, Tara SFiedler, RomanRogiers, VeraFiehn, Anne-Marie KanstrupRoh, MiinFigee, MartijnRoh, Seung YoungFigueras-Aloy, JosepRohaim, MohammedFigueras-Alvarez, OscarRohde, KerstinFiliou, MichaelaRohde, Palle DuunFilipovic, MiodragRohira, AartiFilippi, FrancescaRohren, Eric M.Filocamo, GiovanniRöhrig-Herzog, GabrieleFilosi, MicheleRohsenow, DamarisFindeklee, SebastianRojo, LuisFine, NoahRojo, RosaFinger, ReginaldRokosh, Donald G.Fink, Jodok MatthiasRolfes, LeoniFink, MaxRolfson, OlaFinkel, Alan G.Rolla, GiovanniFinn, Aloke VirmaniRolle, TeresaFinotti, AlessiaRollino, CristianaFinsterer, JosefRolston, JohnFinzi, AndrésRoma, PaoloFiorentini, Giammaria M.Romagnoli, CeciliaFioretti, AlessandraRoman, JesseFiori, EnricoRomanò, Carlo L.Fiorillo, ClaudioRomano, FedericoFiorillo, LucaRomano, FrancescoFiorina, PaoloRomano, Giovanni LucaFischer, ChristianRomano, MarcoFischer, Nicholas G.Romano, VitoFischer, SebastianRomanov, VictorFisher, BenjaminRomanowicz, JenniferFisher, CynthiaRomao, Vasco C.Fisher, MandyRomeo, EmanueleFisman, SandraRomero Collado, AngelFiz, FrancescoRomero, Juan P.Flaatten, HansRomero, JulianFlaherty, KevinRomero-Martín, MacarenaFlament, Marie-PierreRomeyke, TobiasFlannick, JasonRomiti, Giulio FrancescoFlavell, CarolRommel, FelixFleischman, Angela G.Rommens, Pol M.Fleitas, TaniaRommer, PaulusFlemming, Jennifer A.Rompoti, NataliaFlemming, SvenRonaldson, AmyFleszar, MariuszRondanino, ChristineFletcher, JaneRonen, OhadFleuren, Emmy D. G.Rong, RuichenFleuren, NienkeRönmark, Eva P.Flierl, UlrikeRoohullah, AflahFlint, KelseyRoquilly, AntoineFlippot, RonanRosa, Andreia MartinsFlisiak, RobertRosa, Bruce A.Floh, AlejandroRosa, NicolaFloreani, AnnarosaRosa-Garrido, ManuelFlores, Ann MarieRosato, ValerioFlorescu, MonicaRose, Christopher F.Flores-Guerrero, Jose L.Rose, David Z.Flottmann, FabianRose, DestanieFloyd, Sarah BauerRose, GeoffreyFluge, ØysteinRoselli, FrancescoFluxa, DanielaRosenberg, DanielFoeldvari, IvanRosenberg, MollyFogacci, FedericaRosenberg, NahumFogagnolo, PaoloRosendahl, JonasFoka, PelagiaRosenfeld, DavidFoletto, MirtoRosenkrantz Hölmich, EmmaFolkvardsen, Dorte BekRosenow, TimFonnes, SivRosenthal, KenFonseca, Bruno Miguel Reis DaRosenwasser, LannyFonseca, FernandoRosenzweig, AllisonFonseca, FrancinaRoser, SteveFontana, FrancescoRosés, Josep LupónFoong, Rachel E.Rosner, JanFord, Anthony MRoss, GlynisFord, John C.Ross, Kharah M.Ford, JonRoss, Rachel A.Ford, JonathanRoss-Adams, HelenForget, PatriceRossaint, JanForis, VasileRossi, DavideForlano, RobertaRossi, GianpaoloForlini, MatteoRossi, MichelaFormenti, FedericoRossi, NoreenFormenti, PaoloRossi, Noreen F.Formica, FrancescoRossi, TommasoFornes, RominaRossides, MariosForni, MonicaRossing, KasperForouzanfar, TymourRossini, ZefferinoForshaw, MarkRossman, MiltonForster, JamesonRosso, NataliaForsyth, MarkRossow, Lindy M.Forsythe, RachaelRostagno, CarloFort, Patrice E.Rostoker, GuyForte, AlbertoRoszkowska, AnnaForte, MaurizioRota, MatteoForte, RaimondoRotella, Joe-AnthonyFortin, MaryseRoth, AndreasFortis, Sotirios P.Roth, ChristianFortunato, LeonzioRoth, DominikFortuny, ClàudiaRothe, ThomasFosbøl, Marie ØbroRothhammer, VeitFoschini, M. P.Rothwell, DominicFoschino-Barbaro, Maria PiaRotter, GabrieleFossali, TommasoRotter, IwonaFossmark, ReidarRoudier, EmilieFoster, DavidRoulston, Carli L.Foster, Toshana LauriaRoumeliotis, Theodoros I.Foti, Daniela P.Rouzet, FrançoisFoutch, Brian K.Rovaris, MarcoFowler, Jessica C.Rovas, AlexandrosFox, Erin ElizabethRoviello, GiandomenicoFoy, BrodyRovina, NikolettaFraldi, AlessandroRovo, AliciaFranceschi, AlessandroRowe, Steven P.Franchineau, GuillaumeRowley, JamesFranchini, MassimoRoy, Denis J.Francis, Ian C.Roy, RosaFrancisco, Javier Alvaro-AfonsoRoy, Soumyajit D.Francoeur, Charles L.Royall, Donald R.Frankewycz, BorysRoyds, JonathanFranko, AndrasRoyster, Roger L.Franko, JanRoza, Badr-EslamFransen, PaulRozalski, MarcinFranza, LauraRóżańska-Walędziak, Anna M.Fraser-Bell, SamanthaRozec, BertrandFrasoldati, AndreaRozek, WojciechFrassanito, Maria AntoniaRozen, RimaFrater, John L.Ruano, RodrigoFrauenfelder, ThomasRubattu, SperanzaFreedberg, Daniel E.Rubello, DomenicoFreedman, Mark S.Ruben, PeterFreeman, Michael D.Ruben, Robert J.Freitas De Souza, RaphaelRubin, MosheFrenkiel, SaulRubino, ElisaFreo, UldericoRubio, GabrielFreund, AnneRubio-Rivas, ManuelFreund, MatthiasRubis, PawelFrey-Law, Laura A.Ruchała, MarekFreyschlag, Christian F.Rudert, MaximilianFrich, Lars HenrikRudrappa, MohanFricker, MichaelRuebner, Rebecca L.Frickmann, HagenRueda-Domínguez, AntonioFrieboes, HermannRuelle, JeanFried, JustinRuet, AlexisFriedman, ElliottRuf, Tobias F.Friedman, Kevin G.Ruf, Viktoria C.Friedman, Matthew J.Ruffinatti, Federico AlessandroFriedrich, GuyRufini, AlessandroFriis-Hansen, LennartRuggieri, AnnaFrimodt-Møller, NielsRuggieri, PietroFrisch, Steven M.Ruggiero, AntonioFrisso, GiuliaRuggiero, BarbaraFrizelle, FrankRuggiero, MarcoFröhlich, HannaRugonyi, SandraFrohnhofen, HelmutRuiz Giardin, Jose ManuelFrosi, AlbertoRuiz Ibán, Miguel A.Frostegard, JohanRuiz, SantiagoFrucht, Steven J.Ruiz-Fernández, María DoloresFrugé, Andrew D.Ruíz-Muñoz, MaríaFrye, BjörnRullo, RosarioFuchs, Hans F.Rumrich, Isabell K.Fuchs, Hans FriedrichRunkle, IsabelleFuchs, Piotr A.Runtuwene, Lucky RonaldFudulu, DanielRuoppolo, GiovanniFuegger, ReinholdRuppert, ElisabethFugger, GernotRuprecht, Ruth M.Fuggle, NicholasRurali, EricaFuji, ShigeoRusciano, Maria RosariaFujii, HidekiRuss, Mark J.Fujii, ShoichiRussell, David G.Fujimori, NaoRussell, RichardFujimoto, KazukiRusso, AlessandroFujimoto, KeisakuRusso, Francesca MariaFujimoto, YuhiRusso, MarcoFujimura, ShintaroRusso, Mark W.Fujioka, KazumichiRusso, SuzanneFujisawa, MasanoriRustagi, TarushFujita, KazutoshiRustgi, Vinod K.Fujita, KoheiRusy, Lynn M.Fujita, MitsuguRuszkowska-Ciastek, BarbaraFujiwara, KenjiRutkowski, Joseph M.Fujiwara, NatsumiRutkowski, PiotrFukagai, TakashiRutkowski, PrzemysławFukuda, MinoruRutkowski, SebastianFukuhara, AtsunoriRutland, Catrin SianFukuhara, YasuyukiRutledge, DanaFukui, ShinjiRuwanpura, Saleela M.Fukumura, YukiRuzsanyi, VeronikaFukunaga, AtsushiRyan, Neil A. J.Fukunaga, NaotoRybakowska, IwonaFukushima, HiroshiRybka, Jakub DaliborFukuta, HidekatsuRybtsov, StanislavFulciniti, FrancoRychter, Anna MariaFuller, MariaRyder, Robert E. J.Fuller-Tyszkiewicz, MatthewRymarz, AleksandraFung, Isaac Chun-HaiRynio, PawełFung, MonicaRyscavage, PatrickFurian, MichaelRysz, JacekFurlan, AndreaRyu, ChangwanFürnau, GeorgRyu, Ji-wonFuruichi, KengoRyu, Seon YoungFurukawa, HiroshiRzońca, PatrykFuruta, TakuyaS. Dalal, DeepanFurutani, KentaSaada, AnnFusaroli, PietroSabatino, LauraFusar-Poli, LauraSabbagh, CharlesFusco, AugustoSabbatinelli, JacopoFusco, VittorioSabbatucci, MichelaFushida, SachioSabet, ShereenGabaldón, ToniSacanella, Emilio S.Gaballah, AhmedSaccardi, CarloGabor, Alin-GabrielSacchetti, BenedettoGabr, Refaat E.Sacco, EmilioGabriel, AuraSacco, GuillaumeGabriel, EmmanuelSachar, David BGabriel, MaximilianSachithanandan, NirupaGabriel, MichaelSacitharan, Pradeep KumarGabriela, Da Silva XavierSadaghianloo, NirvanaGabryelska, AgataSadaoka, TomohikoGadad, Bharathi S.Sadasivam, MohanrajGadad, Bharathi ShrikanthSadeghi, HosseinGaddam, Ravinder ReddySadeghpour Heravi, FatemahGadjeva, MihaelaSadowski, MarcinGaetani, EleonoraSaeed, FahadGaffo, Angelo L.Saeed, SahraiGagliano, CaterinaSaeki, ChisatoGagneux, PascalSaeki, KumikoGagnon, Joseph CalvinSáez-González, EstebanGaillard, C. A. J. M.Safavi-Naeini, PayamGaitskell, KeziaSaglietti, FrancescoGajewski, Jerzy B.Sagong, MinGajos-Michniewicz, AnnaSah, NirnathGala, Manish K.Saha, Amit KumarGalan, MariaSaha, NirmalyaGalanti, GiorgioSaha, ProgyaparamitaGalasso, GiovanniSahu, Kamal KantGalazios, GeorgiosSaibene, Francesca LeaGaleazzi, Gian MariaSaisho, YoshifumiGalelli, LucaSaito, AyaGalgani, MarioSaito, MayukoGaliniak, SabinaSaito, SatoruGalipeau, JacquesSaito, TakaoGallamini, MicheleSaito, YoshinobuGallardo, M. EstherSaito, YuichiGallardo-Vara, EunateSaito, YukihiroGallavardin, ThibaultSaitoh, TakejiGallego-Colon, Enrique J.Saiwai, HirokazuGalletti, FrancescoSak, Jarosław J.Galli, AndreaSakai, ArataGalli, JacopoSakai, FumikazuGallie, BrendaSakai, TetsuroGALLO, AntonioSakakura, KenichiGallo, GaetanoSakamaki, AkiraGallotta, ValerioSakamoto, MasayaGalosi, AndreaSakhaee, KhashayarGaltung, Hilde KanliSakkas, Lazaros I.Galvan, CristinaSakoda, Lori C.Galvez Sánchez, Carmen M.Sakuma, ShigemitsuGaly-Fauroux, IsabelleSaladini, FrancescoGambardella, ClaudioSalazar, Juan J.Gambardella, JessicaSalazar-Mendiguchía, JoelGambarini, GianlucaSalazar-Torres, Jose De JesusGambaruto, AlbertoSaleh, AarashGame, Frances L.Salem, AhmedGammie, AndrewSalerno, SergioGamus, DoritSalim, Sohail AbdulGan, Gregory N.Salit, Irving E.Gandhi, Sagar S.Salkini, MohamadGandini, PaolaSallustio, FabrizioGandolfi, StefanoSalm, Adrienne K.Gandomkar, ZibaSalom, Caroline L.Ganea, DoinaSalomone, AlbertoGanger, RudolfSalonia, AndreaGangula, PanduSalton, FrancescoGani, FedericaSalvadori, NicolaGanini, CarloSalvatore, SilviaGantz, Marie G.Salvetat, Maria LetiziaGao, DingchengSalvi, FabioGao, DongxuSalvi, MarioGaragiola, UmbertoSalzano, AndreaGaraicoa-Pazmino, CarlosSalzwedel, AnnettGarber, Carol EwingSamaan, Mark A.Garcia Doval, IgnacioSamadi, Sayyed AliGarcía Iglesias, DanielSamantaray, SupritiGarcia Iglesias, PilarSamarska, IrynaGarcia, Alejandro V.Samavati, LobeliaGarcía, AngelSambataro, SergioGarcia, Emilio GutierrezSambri, AndreaGarcia, SamuelSamidurai, ArunGarcía-Álvarez, YolandaSammi, ShreeshGarcía-Aranda, MarilinaSammut, Ivan A.García-Arranz, MarianoSamochocki, ZbigniewGarcía-Azorín, DavidSamochowiec, JerzyGarcía-Barrado, María JoséSamy, RaviGarcía-López, SantiagoSanada, HiromiGarcía-Malinis, Ana JuliaSanak, MarekGarcía-Marcos, LuisSancassiani, FedericaGarcía-Muñoz Rodrigo, FerminSánchez, EnricGarcía-Pola, María JoséSánchez-Bueno, FranciscoGarcia-Quintanilla, AlbertSánchez-Castellano, CarmenGarcía-Rey, EduardoSánchez-Ferrer, Maria L.García-Romero, JeronimoSánchez-Garcés, María ÁngelesGarcin, ThibaudSánchez-González, José-MaríaGard, PaulSánchez-González, María CarmenGardner, Thomas W.Sanchez-Perez, ArturoGardrat, SophieSanchez-Solis, ManuelGareth, NyeSánchez-Torres, AlbaGaretz, Susan LynnSanchiz, ÁfricaGarg, KawishSancho-Chust, Jose N.Garg, TullikaSancisi, ValentinaGarin, NicolasSandblom, GabrielGarneau, JonathanSanders, Jan StefanGarner, AngieSandhu, Satinder K.Garofalo, EugenioSandrini, LeonardoGarrabou, GloriaSandritter, Tracy L.Garriboli, MassimoSands, Jeff MGarrison, Roger C.Sandset, P. M.Garvin, Kevin L.Săndulescu, MihaiGarweg, Justus G.Sandy-Hodgetts, KylieGarzon-Muvdi, TomasSangiao-Alvarellos, SusanaGarzorz-Stark, NatalieSangineto, MorisGasana, JanvierSango, KazunoriGąsior-Perczak, DanutaSanguigni, ValerioGaspar, RuiSanidas, EliasGasparini, PatriziaSankar, TejasGasparini, SaraSankarasubramanian, VishwanathGasparro, RobertaSanna, FabrizioGasparski, AlexanderSano, DaisukeGasparyan, ArmenSantamaría, RafaelGassensmith, JeremiahSantangelo, Kelly S.Gastman, Brian R.Santarelli, AndreaGaston, Tyler E.Santarpino, GiuseppeGatta, RobertoSanter, DavidGatto, RobertoSanthanam, AbiramiGatz, MatthiasSanti, DanieleGatzoulis, Konstantinos A.Santorelli, Filippo M.Gaudet, AlexandreSantoro, AnnaGaudet, DanielSantoro, JonathanGauer, StefanSantos Vizcaíno, EdortaGaul, CharlySantos, FernandoGautam, MukeshSantos, Josefa GonzálezGautam, SnehaSantos, LuisGautheron, JérémieSantos, MarianaGauthier, SergeSantosa, HendrikGauthier, TristanSantos-Cortez, RegieGava, GiuliaSantos-Gallego, CarlosGavaruzzi, TeresaSantos-Juanes, JorgeGavriilaki, EleniSantos-Lozano, AlejandroGawęcki, MaciejSantos-Rosales, GuidoGawlikowski, MaciejSantulli, GaetanoGaze, David C.Santus, PierachilleGaze, MarkSanz, AlejandroGazouli, MariaSanz, RebecaGe, XiaodongSanz-Corbalán, IreneGea-Banacloche, Juan CarlosSanzo, AntonioGebhardt, RolfSapalidis, KonstantinosGedamu, LashitewSaponaro, ChiaraGedeon, TomasSaponaro, F.Gee, Heon YungSapudom, JiranuwatGehr, Todd W. B.Sarasija, ShaarikaGeijer, MatsSardu, CelestinoGeisinger, MariaSarecka-Hujar, BeataGeller, James I.Sarigiannis, DimosthenisGelsomino, SandroSaritas, TurgayGelzo, MonicaSarkar, AmritaGembillo, GuidoSarkar, ArijitaGembruch, OliverSarker, MarjanaGennarelli, GianlucaSarmati, LoredanaGenovesi-Ebert, FedericaSarnthein, JohannesGentile, CarmineSarrias, AxelGentile, LucaSartipy, UlrikGentile, MattiaSarzi-Puttini, PiercarloGentileschi, StefanoSasabuchi, YusukeGentzler, Ryan D.Sasai, HiroyukiGeoffroy, Pierre A.Sasaki, KaoruGeorgakarakos, EfstratiosSasaki, YousukeGeorge, KoumantakisSasamoto, NaokoGeorgiev, StanimirSasanabe, RyujiroGeorgiou, IoannisSasegbon, AyodeleGerasimenko, OlegSatake, HironagaGerbershagen, Mark UlrichSato, AkiraGerding, HeinrichSato, TakuichiGerke, OkeSato, TatsuhikoGerlach, HerwigSatou, RyousukeGerocarni Nappo, SimonaSatterstrom, KyleGerreth, KarolinaSattler, SusanneGerson, TrevorSaturnino, CarmelaGerstenecker, AdamSaul, DominikGerussi, AlessioSaulacic, NikolaGesi, CamillaSauro, SalvatoreGesuita, RosariaSaus, J. ArthurGeva-Zatorsky, NaamaSausaman, Robert W.Gevirtz, RichardSavarino, VincenzoGezer, DenizSavaşan, SüreyyaGhabril, Marwan S.Savioli, GabrieleGhadieh, Hilda E.Savolainen-Peltonen, HannaGhafouri, BijarSawada, AkinariGhaisas, ShivaniSawada, KojiGhamrawi, RimaSawahata, MichiruGhanaati, ShahramSaxena, SugandhaGhandour, RashedSaxer, FranziskaGhantoji, Shashank S.Sayar, HamidGharbiya, MagdaSayour, Elias J.Ghayee, HansScacchi, MassimoGhazani, MaryamScalfari, AntonioGhaznavi, SanaScannapieco, Frank A.Ghidini, AlessandroScanu, Antonio MarioGhiggia, AdaScarano, AntonioGhigliotti, GiorgioScarinci, FabioGhilli, MatteoScarlata, SimoneGhimessy, Áron KristofScavello, IreneGhorbani, MortezaSchaaf, HeidrunGhosh, AsishSchaefer, BenediktGhosh, Paramita M.Schaefer, MichaelGhosh, SumanaSchaefer, Sebastian D.Ghosh, Tarini ShankarSchäfer, AndreasGhouri, YezazSchapher, MircoGiacobbe, Daniele RobertoSchar, Ralph T.Giacobbe, PeterScharffenberg, MartinGiacobini, MaiBrittSchattman, Glenn L.Giacomelli, AndreaSchaub, Günter A.Giacomelli, LucaSchechter, Alan NeilGiallauria, FrancescoScheepers, RenéeGiamberardino, Maria AdeleScheffel, JörgGianfaldoni, SerenaScheinker, DavidGiangrande, Gian PaulSchellart, René PGiani, MarcoSchellenberg, Morgan A.Giannakopoulos, Georgios F.Schellino, RobertaGiannakopoulos, NikolaosSchemmer, PeterGiannecchini, SimoneScherer, ThomasGiannetto, ClaudiaSchermer, BernhardGiannì, Aldo BrunoScherrer-Crosbie, MarielleGiannone, GaiaScherschel, KatharinaGiannoni, PatriziaSchiavi, Michele CarloGiannotta, StevenSchiavon, SoniaGianturco, LuigiSchiavone, CosimaGiardina, EmilianoSchiavone, NicolaGiardino, AlessandroSchiefer, Jennifer L.Gibbons, AllisterSchierz, OliverGibbons, JeffSchiffer, LinaGibbs, KevinSchiffner, ReneGibson, MarkSchildberg, Frank A.Gibuła-Tarłowska, EwaSchilsky, MichaelGierula, MagdalenaSchiltenwolf, MarcusGiessing, MarkusSchindlbeck, Katharina A.Gigante, AntoniettaSchini-Kerth, ValérieGigli, Gian LuigiSchiøttz-Christensen, BeritGil, JustynaSchippers, AngelaGil, PedroSchiweck, CarmenGil, ZivSchizas, A. M. P.Gilbert, Duncan C.Schizas, NikosGilbert, FrédéricSchlabritz-Loutsevich, NataliaGilchrist, NigelSchlatter, AndreasGildner, Theresa E.Schleim, StephanGill, ChandlerSchleiss, MarkGillard, Baiba K.Schlembach, DietmarGillet, GermainSchlereth, Simona L.Gillingham, Melanie B.Schluger, Neil W.Gimenez, Maria RubiniSchmälzlin, ElmarGimeno, HortensiaSchmelter, CarstenGimeno, Juan RamónSchmidberger, HeinzGinevra, ChristopheSchmidl, DoreenGingele, StefanSchmidt, AlexanderGioia, Laura C.Schmidt, Peter ThelinGiollo, AlessandroSchmidt, SamuelGiordano, CarlaSchmidt-Braekling, TomGiordano, FlavioSchmitt, Charles P.Giovanni, FrisulloSchmitt, JoachimGiovanni, MerlinoSchmitter, MarcGiovingo, Michael CarloSchmitz, RalfGirard, JulienSchmitz, UlfGirardi, PaoloSchmoch, ThomasGirardis, MassimoSchmutzler, AndreasGiraudeau, NicolasSchnaubelt, SebastianGiraudo, ChiaraSchneider, BirkeGisselbrecht, ChristianSchneider, DavidGiudice, AmerigoSchneider, KristianGiudice, Giuseppe LoSchneider, MatthiasGiuffrè, MauroSchneider, Uffe VestGiuntoli II, Robert L.Schneiderhan-Marra, NicoleGiuseppe, VarvaraSchnell, FrédéricGiusti, AndreaSchnetzke, MarcGkantidis, NikolaosSchnutenhaus, SigmarGkolfakis, ParaskevasSchoemaker, Minouk J.Głąbska, DominikaSchoen, Robert T.Gladding, Patrick A.Schoenecker, Jonathan G.Gladysheva, Inna P.Schoenhagen, PaulGlassman, Patrick M.Scholer, Anthony J.Glaus, JenniferScholey, JamesGleeson, NigelScholtens, AsbjørnGlenn, WendySchoon, JanoschGlibert, MaartenSchoon, YvonneGlinkowski, WojciechSchöttle, DanielGlize, BertrandSchrage, BenediktGlocker, Michael O.Schramm, PatrickGlockner, KarlSchredl, MichaelGloor, BeatSchreinlechner, MichaelGlossop, NeilSchroeder, Harry W.Glover, Anthony R.Schuettfort, GundolfGlowniak, AndrzejSchulman, SamGluck, NathanSchulthess, BettinaGlue, PaulSchulz, JaneGlueck, Amanda C.Schulz, SusanneGnanamony, ManuSchulze, HaraldGniadecki, RobertSchulze-Tanzil, GundulaGöbl, Christian S.Schulz-Kornas, EllenGodfrey, RineshSchumacher, UdoGodino, CosmoSchure, MarkGodsel, Lisa MarieSchutt, AmyGoehring, TobiasSchwameis, MichaelGoel, GauravSchwartz, Robert A.Goel, ShreyaSchwartz, StevenGoetz, ChristopherSchwartzman, MonicaGoffart, SteffiSchwarz, AnkeGogulamudi, Venkateswara ReddySchwarze, VincentGohy, SophieSchweiger, MartinGoicoechea, CarlosSchweigkofler, UweGoldfarb, MichaelSchwierzeck, VeraGoldhoorn, Robert Jan BerendSciomer, SusannaGoldstein, TraceyScioscia, GiuliaGoldsweig, Andrew M.Scofield, Robert Hal A.Gołębiowski, TomaszScorziello, AntonellaGoliasch, GeorgScott, DanielGöllner, StefanieScott, EmmaGolpe, RafaelScott, SimonGomatou, GeorgiaScotto D'Abusco, AnnaGomes-da-Silva, Lígia C.Scuderi, GianlucaGomez, Maria LauraScudiero, RosariaGomez-Aristizabal, AlejandroSdrulla, Andrei D.Gómez-Barrena, EnriqueSeaby, EleanorGómez-Casares, Maria TeresaSeals, Brenda F.Gomez-Gil, VeronicaSeáñez, IsmaelGómez-Huelgas, RicardoSebastian, AimyGómez-Mayordomo, VíctorSebastianelli, ArcangeloGomez-Pastrana, DavidSebastiani, MarcoGomez-Pinilla, Pedro J.Secchi, FrancescoGómez-Urquiza, Jose L.Secco, Gioel GabrioGomi, FumiSeco-Calvo, JesusGompel, Anne A.Secor, EricGon, YasufumiSecord, Angeles AlvarezGonçalves, HernâniSedaghat, AlexanderGong, KuangSedda, GiuliaGoniewicz, MariuszSedlak, LechGono, TakahisaSeeherunvong, TossapornGonoi, WataruSeeman, Mary V.Gonsalves, Wilson I.Seers, ChristineGonska, TanjaSegala, DiegoGonthier, Marie-PauleSegatto, MarcoGonzales, MelissaSeghaye, Marie-ChristineGonzales, Mitzi M.Seguí-Ripoll, José MiguelGonzález Rodríguez, AguedaSegura, Juan-JoseGonzález, JerónimoSegura, Leal G.González, Jose AntonioSeheult, Jansen N.Gonzalez, Raul S.Seibert, Franz JosefGonzalez-Barcala, Francisco-JavierSeidler, RachaelGonzález-Bulnes, AntonioSeifert, Hans-HelgeGonzález-Clemente, José MiguelSeiff, Stuart R.Gonzalez-Dunia, DanielSeike, MasahiroGonzález-Escribano, María FranciscaSeiler, StephenGonzalez-Juanatey, CarlosSeixas, AdéritoGonzalez-Menendez, PedroSekino, YoheiGonzález-Muñiz, RosarioSelberherr, AndreasGonzalez-Reyes, Luis E.Selk, AmandaGonzalez-Rodriguez, AlexandreSellarés, JacoboGood, PhillipSellner, FranzGoodall, StuartSelva-O'Callaghan, AlbertGoodla, LavanyaSelvaraju, VaithinathanGoodman, Lauren F.Sembrano, Jonathan N.Goodman, Michael D.Semczuk, AndrzejGoodwin, RobinSeminari, ElenaGoonewardene, SanchiaSemmler, MarionGoossens, QuentinSemova, IvanaGopalakrishnan, RaghavanSen, H. N.Gorczyca, IwonaSen, Monokesh KumerGorczynski, AdamSen, RwikGordish-Dressman, HeatherSena, ArmandoGordon, John D.Senbonmatsu, TakaakiGorgen, AndreSenderovich, HelenGori, TommasoSendra-Portero, FranciscoGorini, GiuseppeSenesi, PamelaGoris, TobiasSengupta, NeilGörlich, DennisSenneville, EricGornowicz, AgnieszkaSeo, BenedictGorodzinsky, AyalaSeo, Cheong HoonGorrell, MarkSeo, Eun JiGorrell, RebeccaSeo, HyungseokGorringe, KylieSeo, JinsooGorriz, JoseSeo, KyoungYulGorshkov, KirillSeo, SatoruGórska, KatarzynaSeo, YoshihiroGórska, SabinaSeok, HyeriGorski, BartlomiejSeok, HyunGorski, Justin W.Sepúlveda, Ana R.Gosav, Evelina MariaSeracchiani, MarcoGosciniak, GrazynaSeracchioli, RenatoGossage, James R.Serafini, SimoneGoto, HiroshiSerebruany, VictorGotta, VerenaSerena, GloriaGötte, MatthiasSergi, ConsolatoGottschalk, AllanSerralheiro, PedroGottschalk, William KirbySerrano, AntonioGoulet, Jean-PaulSerruys, Patrick W.Goumenou, MarinaSeto, Arnold H.Goussia, Anna C.Seto, ToshiyukiGovetto, AndreaSetoguchi, ShuichiGowda, Siddabasave B.Seubert, Christoph N.Goździewska-Harłajczuk, KarolinaSeverini, CinziaGrabarek, Beniamin OskarSeverino, PaoloGrabczak, Elżbieta MagdalenaSevilla, IsabelGracco, AntonioSévoz-Couche, CarolineGracia Garcia, PatriciaSeward, EdwardGraco, MarnieSewerin, PhilippGradalski, TomaszSexton, EithneGrady, Erin E.Sfakianaki, MariaGraef, FrankSferrazza Papa, Giuseppe FrancescoGraf, NorbertSgambat, KristenGragnaniello, CristianSgarzani, RossellaGragnano, FeliceShaffer, Virginia O.Graham, Michael M.Shafiee, AbbasGrambow, EberhardShafique, MichaelGrammatico-Guillon, LeslieShah, AdityaGrammatopoulos, GeorgeShah, Ashish H.Grand, JohannesShah, NeerajGräni, ChristophShah, Samir A.Grant, Gerald T.Shah, SamitGrape, SinaShahmoradi, MahdiGras, DelphineShaker, AnisaGrasl, StefanShalayel, IbrahimGrassi, Federico A.Shamah, Steven P.Gratz, KlausShamberger, RobertGrau, Juan B.Shamieh, SaidGrau, VeronikaShan, BerginGraupner, OliverShan, YiGrautoff, SteffenShanahan, FergusGray, Peter B.Shander, AryehGrazette, LuandaShangguan, SiyiGrazia Piancino, MariaShank, Lisa M.Greco, MartaShankar, EswarGreen, ClaraShankarappa, Sahadev A.Greenberg, ArthurShanmugam, MuruganandanGreenberg, JonathanShao, DangdangGreengard, Emily G.Shapiro, RonGreenhalgh, DavidSharabi, KfirGreening, NeilSharma, AnuragGreenland, JohnSharma, AshuGregg, LucileSharma, AtulGreggi, TizianaSharma, Bhesh RajGregori, GiovanniSharma, GayatriGregori, MarkusSharma, GeetanjaliGregorini, MarilenaSharma, IshaGreinacher, AndreasSharma, RavindraGreiner, Jack V.Sharma, Samin K.Greiner, StefanSharma, ShwetaGreitemeyer, TobiasSharma, Tasneem P.Gremmel, ThomasSharma, Vibhash D.Grennan, TroySharma, VikramGridnev, Vladimir I.Sharman, Edward H.Grieshaber, PhilippeSharp, Willard W.Griffin, MoragShaw, JamesGriffioen, MariekeShayesteh, ShahabGriffith, MayShearer, Aiden EliotGrill, EvaShearer, Freya M.Grillo, AndreaShefer, GabiGrillon, AntoineSheha, HosamGrimm, Marcus O. W.Sheikh, Søren PaludanGrimm, W. D.Sheils, OrlaGrinberg, KerenSheiman, LauraGrippaudo, CristinaSheldrick-Michel, TanjaGris, Jean ChristopheShelley, BenjaminGriswold, DavidShemtov-Yona, KerenGröbe, AlexanderShen, HuiGrochot-Przeczek, AnnaShen, LongGroeschel, SamuelShen, MengchengGrohs, Josef GeorgShen, YangGromoll, JörgSheng, YueGrossini, ElenaShenhar-Tsarfaty, ShaniGrothe, ClaudiaShenoy, AkhilGrover, SandeepShepard, Blythe D.Gruartmoner, GuillemShephard, Mary N.Grubitzsch, HerkoSherif, Zaki A.Gruessner, Rainer W. G.Sheshadri, AjayGrundy, LukeShetty, AmithGrunert, IngridShetty, Sudarshan A.Grunewald, RichardSheu, Eric G.Grünweller, ArnoldShi, HaifeiGruter, BasilShi, JunchaoGrützner, Paul AlfredShi, LuGrymowicz, MonikaShibata, TomoyukiGrywalska, EwelinaShibata, YasushiGrzech-Lesniak, KingaShigeta, ShogoGrzela, TomaszShimada, HiroyukiGrzybowski, AndrzejShimada, MasayukiGrzywacz, AnnaShimada, YoshiakiGu, BinShime, NobuakiGu, YingliShimizu, FumiakiGuadagni, StefanoShimizu, TakayoshiGuala, AndreaShimizu, TakeyukiGuaricci, AndreaShimizu, YujiGuarnieri, GabriellaShimoda, KazuyaGuarnieri, MarcelloShimoda, ShinjiGubbi, SriramShimohira, MasashiGuber, IvoShimura, TatsuoGuber, JosefShin, Byung-CheulGuccione, JuliusShin, Hae-WonGuccione, PaoloShin, Hyoung-shikGuedeney, PaulShin, HyungooGuehring, ThorstenShin, Myung-junGuenancia, CharlesShin, SangahGuenther, UlfShin, Seung YongGuerini Rocco, ElenaShin, Yong UnGuerra, FrancescoShin, YooseokGuerraz, MichelShinde, AparnaGuerrero-Aspizua, SaraShingu, YasushigeGuerrero-Mosquera, CarlosShinoda, KeiGuerriero, IlariaShinohara, ShogoGuetl, KatharinaShirai, JyunsukeGuhathakurta, SubhrangshuShirai, ToshihiroGuicciardi, MarcoShiraishi, NaruGuilbaud, TheophileShirvalkar, PrasadGuillamat-Prats, RaquelShitara, HitoshiGuillén-Del-Castillo, AlfredoShiva, SrutiGuillon, AntoineShivakumar, PranavGuilpain, PhilippeShivanna, BinoyGuirguis, AmiraShivapurkar, NarayanGuisado-Vasco, PabloShiwarski, Daniel J.Gulati, KaranShmidt, EugeniaGulati, RohitShoaib, AhmadGulati, SwatiShoemark, AmeliaGuljé, Felix LouisShoji, FumihiroGullick, NicolaShook, Robin P.Gunaje, JayaramaShore, SusanGunasekar, Susheel K.Shores, Darla RoyeGundamaraju, RohitShosha, EsraaGundermann, Karl JosefShpiner, DanielleGundlach, Jan-PaulShrira, AmitGunn, Jarmo MikaelShteinberg, MichalGunning III, William T.Shukla, AshutoshGuns, Pieter-Jan D.Shukla, KirtikarGünther, AndreasShukla, Surendra KumarGuo, Haiwei HenryShulman, MatisyahuGuo, XingyiShultz, SandyGuo, Zhi-LingShung, DennisGupta, AbhaShyamsundar, MuraliGupta, KuldeepSiafakas, NikolaosGupta, Sushilkumar SatishSiddappa, ByrareddyGursoy, UlviSideris, SkevosGursoy, Ulvi KahramanSidiropoulos, ChristosGuru, Pramod K.Sidiropoulos, KonstantinosGurung, Pratik M.Sidorkiewicz, IwonaGustafsson, PeikSidtis, John J.Gutaj, PawełSié, PierreGuthoff, MartinaSiegel, ShannonGuthrie, ElseSiegler, SorinGutiérrez Abejón, EduardoSievers, Laura KatharinaGutieŕrez, EduardoSievi, Noriane A.Gutmann, James LeoSifakakis, IosifGuyton, DavidSigdel, TaraGuzeloglu-Kayisli, OzlemSignore, AlbertoGuzmán-Vélez, EdmarieSignorile, AnnaGuzzi, FrancescoSijts, AliceGyselaers, WilfriedSikora, MariuszH. Wein, TheodoreSiles, EvaHa, Min-SeongSilva, AbigailHa, YeonjungSilva, Ana RitaHa, Yun-SokSilva, Anabela G.Haapasalo, HeidiSilva, Frutuoso G. M.Haarmann-Stemmann, ThomasSilva, JoanaHaas, NikolausSilva, MarcoHaas, VerenaSilva, RebekaHaase-Fielitz, AnjaSilva, Scott R.Habel, UteSilverio, Sergio A.Habiba, MarwanSilverstein, MadisonHachisu, YoshimasaSilvestre-Rangil, JavierHadamitzky, CatarinaSilvestri, GiovanninoHadar, EranSim, Hao-WenHadden, Jodi A.Simao, TatianaHadid, TarikSimar, DavidHadj-Moussa, MiriamSimard, J. MarcHadley, GinaSimecka, Jerry W.Hadzik, JakubSimianu, Vlad V.Haecker, Hans R.Simmen, Hans-PeterHafer, CarstenSimms, VictoriaHage, CamillaSimoes, Davina Camargo MadeiraHage, RenéSimon, Jessica R.Hagemann, JanSimon, JorgeHagendorff, AndreasSimon, LukeHagihira, SatoshiSimon, Michael S.Hagiwara, AkifumiSimone, GiuseppeHagiwara, KatsuroSimone, LaniniHagiwara, ShuichiSims, JaneHagiwara, YoshihiroSimula, SakariHagiya, HideharuSinakos, EmmanouilHagler, Donald J.Sinclair, DavidHagmann, SébastienSinex, Donal G.Hahnel, SebastianSingal, NeeraHaider, AhmedSinger, GeorgHaider, LukasSingh, AnandHaig, DavidSingh, BhajanHain, Timothy C.Singh, ChandraHaindl, RichardSingh, FavilHaj-Hosseini, NedaSingh, KamayaniHajjar, Emily R.Singh, MandeepHajra, AdrijaSingh, NaveenHakansson, CaritaSingh, NishaHakenberg, Oliver W.Singh, ParamHakim, AlanSingh, RahulHakovirta, Harri H.Singh, RajbirHakuta, RyunosukeSingh, Reetu R.Halachmi, SarelSingh, SandeepHaldorsen, Ingfrid S.Singh, SeemaHale, BeverleySingh, ShantanuHalfon, MatthieuSingh, SukhvinderHalim, MohammadSingh, VijayHalimi, Jean-MichelSingla, BhupeshHall, Jeffrey W.Sinha, NiharikaHall, Nathan C.Sinha, TirthankarHallak, MordechaiSiniscalco, DarioHalle, Mari K.Sinjab, KhaledHallegraeff, JoannesSinnathurai, PremaraniHalliday, DavidSions, J. MeganHalliday, Drew W. R.Siontis, George CMHallifax, RobSiontis, Konstantinos C.Halloran, KieranSiotos, CharalamposHalperin, JohnSiqueira, RafaelHalsey, ChristinaSiraj, AishaHalvachizadeh, SaschaSiravegna, GiuliaHalvatsiotis, Panagiotis G.Sirignano, PasqualinoHam, Won-sikSirvent, RamonHamaguchi, ShogoSisley, Stephanie R.Hamamoto, TakaoSistla, SrinivasHamanishi, JunzoSiudak, ZbigniewHamann, SteffenSivapalan, PradeeshHamaoka, TakutoSivaprakasam, SathishHamaoui, KarimSivaprasad, SobhaHamasaki, HidetakaSjögren, MagnusHamasaki, YoshifumiSjöström, MatsHamazaki, NobuakiSkarzynski, Piotr HenrykHamblin, Peter SSkiba, Dominik S.Hamdi, MoustaphaSkinner, BenHameed, IrbazSkinner, Jonathan RHamid, Abdullah AlSkoglund, KristoferHamilton, Mark G.Skopouli, FotiniHamlett, Eric D.Skorska, AnnaHammer, AnneSkov, MarianneHammer, AstridSkrifvars, MarkusHammer, SuntreaSkrifvars, Markus BHamprecht, KlausSkrinjar, IvanaHampton, AdrienneSkrobot, WojciechHampton, Lance J.Skrzypecki, JanuszHamrefors, ViktorSkvarc, DavidHan, AlexSlade, Jill M.Han, Ian C.Slart, Riemer H. J. A.Han, JinmingSlatter, M. A.Han, Kyung EunSlavin, KonstantinHan, LingSleigh, AlisonHan, MichaelSleigh, JamesHan, MinhoSlieker, Roderick C.Han, PingpingSlimano, FlorianHan, Yueh-YingŚliwińska-Mossoń, MariolaHan, YuyanSloan, J. MarkHanas, JaySloane, Jacob A.Handen, Benjamin L.Slobodin, GlebHandoko, M. LouisSlof-Op 't Landt, Margarita C. T.Handschu, RenéSlominska, Ewa M.Handschuh, LuizaSłomka, ArturHaneke, EckartSlomovitz, BrianHank, ThomasSłowikowska-Hilczer, JolantaHann, Hie-WonSluka, KathleenHanna, AmgadSlukvin, IgorHannaert, PatrickSmaglo, Brandon G.Hannan, Anthony JSmalling, Richard W.Hannawi, BasharSmedler, ErikHannon, EilisSmiroldo, ValeriaHano, TakuzoSmith Jr, Randall J.Hans, GuySmith, AndraHansen, HenrikSmith, Ashley DeanHansen, KeithSmith, ElliotHansen, Kristoffer LindskovSmith, EricHansrivijit, PanupongSmith, Glenn EHanss, MichelSmith, Hayden L.Hansson, Stefan R.Smith, JaredHantschel, OliverSmith, Loren E.Hanyuda, AkikoSmith, MarkHaponiuk, IreneuszSmith, PatrickHaque, Muhammad E.Smith, Rebecca LeeHarada-Shiba, MarikoSmith, Sinead M.Harano, TakashiSmith, Stacy V.Harb, EliseSmith, Stephen W.Hardesty, BrandonSmith, Zachary R.Hardt, SebastianoSmolarczyk, RomanHardtke-Wolenski, MatthiasSmolle, JosefHarduin-Lepers, AnneSmyrnias, IoannisHardwick, James P.Snell, Cudore L.Hargreaves, IainSnelson, MatthewHarila, VirpiSnyder, Diana L.Haring, BernhardSnyder, Orman L.Harjes, ElenaSoares, Fernanda CunhaHarkel, Arend Derk Jan TenSobel, JackHarkin, DamienSobhani, NavidHarky, AmerSöbke, HeinrichHarley, DavidSocea, BogdanHarman, Andrew N.Sochalska, MajaHarmsen, Martin C.Soderlund, GoranHarris, IanSödersten, PerHarris, MarkSoendergaard, MetteHarris, Norman R.Soff, GeraldHarrison, ChristopherSoftic, SamirHarrison, David GlennSofue, TadashiHarrison-Bernard, LisaSoga, YoshihikoHart, RobSogkas, GeorgiosHartemink, Koen J.Sohma, YoshiroHartgers, Merel L.Sohn, Ju-TaeHartikainen, Juha E KSoiza, RoyHartl, ArnulfSokal, PawełHartleb, MarekSokol, Jason A.Hartman, MariuszSokolowska, MilenaHartman, Richard E.Solaberrieta, EnekoHartmann, MatthiasSolaimanzadeh, IsaacHartog, HermienSolal-Giboin, LouisHasan, ProttoySolanki, AshishHasan, Syed ShahzadSolano, PalmaHascoet, SebastienSolano, RubénHasegawa, HirotakaSolarino, GiuseppeHasegawa, MinoruSola-Ruiz, Mª FernandaHasegawa, TakaakiSoldan, Samantha S.Hasegawa, YasushiSolé, CristinaHashida, RyukiSoler, Maria JoseHashim, Ibrahim A.Soles, GillianHashimoto, DaisukeSolinas, CinziaHashimoto, KazuhikoSolís García Del Pozo, JuliánHashimoto, YoshitakaSolomon, LucianHashimoto, YosukeSolopov, PavelHasle, HenrikSolski, JanuszHasni, IssamSoltesz, StefanHasni, SarfarazSommerfeldt, Mark F.Hasoon, JamalSomoza, Rodrigo AHaspula, DhanushSon, KeunBaDaHassan, Ebrahim BaniSoncini, MarcoHassan, Sheref ESones, Amerian D.Hastie, Claire ESong, CherynHata, J. StevenSong, In GyuHatano, KojiSong, Jae MinHatkevich, ClaireSong, Jong-wookHattori, ToshioSong, KunhuaHatzopoulos, StavrosSong, Kyoung SeobHaubruck, PatrickSong, LiujiangHauer, Richard N. W.Song, MingHaug, MarkusSong, Min-juHaugan, KristinSong, Myeong-junHauguel-Moreau, MarieSong, PingHauser, Sheketha R.Song, RuiHauser, W. AllenSong, Young GooHausmann, JohannesSong, YoungseokHaut, KristenSong, YuyuHautz, TheresaSoni, Kiran KumarHavens, Shane J.Sonkar, KanchanHaviv, YaronSonkin, DmitriyHawkins, ShannonSonne, James W.Haxel, Boris R.Sonowal, HimangshuHay, CharlesSophocleous, FrosoHayakawa, KazuichiSoran, Z. OzlemHayakawa, YokuSorbello, MassimilianoHayashi, KatsumaSorbye, Sveinung WergelandHayashi, KazuhikoSörensen, Nils A.Hayashi, MikioSorge, MatteoHayashi, RyujiSoriano-Mas, CarlesHayes, GemmaSorigue, MarcHayton, ConalSorrell, J. MichaelHazama, ShyouichiSorrentino, FlaviaHazan, YenonSorrentino, Francesco SaverioHazeldine, JonSorrentino, SabatoHazrati, Lili-NazSorsa, TimoHe, ShaoqiuSosic, AliceHe, YiSosne, GabrielHealy, AoifeSotiriou, Sotirios K.Heard, CharlesSoto-Cámara, RaúlHeath, P. R.Sotomayor, Camilo G.Hebant, BenjaminSotto, AlbertHebert, Jeffrey RSoudry, EthanHecker, MatthiasSoulage, Christophe O.Heckmann, JosefSoulika, AthenaHedenstierna, GoranSoupene, EricHedges, JodiSousa, DavidHedjazi-Moghardi, MehdiSousa, SóniaHeeg, MaximilianSouth, AndrewHegde, VijaySouthwell, DerekHeger, MichalSouwer, Ibo H.Hehlgans, StephanieSowerby, Leigh J.Hehlmann, RüdigerSoya, HideakiHeiberg, JohanSoyer, PhilippeHeiduschka, PeterSpaans, Anne J.Heijnsdijk, EvelineSpada, FrancescaHeil, Sandra G.Spadaro, SavinoHeinrich, HenrietteSpaggiari, GiorgiaHeir, GarySpagnuolo, RoccoHeise, DanielSpalletta, GianfrancoHeiss, ChristianSpanakis, Elias K.Heitmar, RebekkaSpang, ChristophHejsek, LiborSpanoudakis, EmmanouilHeleniak, ZbigniewSpartalis, EleftheriosHelfrich, Yolanda R.Spartalis, MichaelHeller, JanoschSpearman, PaulHellewell, SarahSpeeckaert, MarijnHelling, Britney A.Spella, MagdaHelliwell, PhilipSpelman, LyndaHellmann, MarcinSpennato, PietroHellström, IngridSperber, GHHellström, StenSperling, Mark A.Helpman, LimorSpiegel, EfratHempel, GuntherSpiegel, MartinHemprich, AlexanderSpielmanns, MarcHendel, HelleSpiering, WilkoHenderson, EmilySpina, Catherine S.Henderson, ReneySpinazzola, GiorgiaHenderson, Robert D.Spinner, Christoph D.Hendicott, Peter L.Spoletini, GabrieleHenen, MorkosSpoletini, GiuliaHenke, OliverSportoletti, PaoloHenkes, HansSprague, Briana N.Hennequin, MartineSprengel, KaiHennerici, Michael G.Spriano, GiuseppeHennessey, James V.Springer, FabianHennig, EwaSpyrantis, AndreaHenningsen, EmilySpyridopoulos, IoakimHenningsson, MarkusSquillaro, TizianaHenrichs, Marcel-PhilippSquires, Ray W.Henriksen, MariusSridharan, MeeraHenriques, JoséSrinivasagan, RamkumarHenrot, PaulineSripathy, SmithaHenry, Brandon MichaelSrivastava, PranayHenry, ClaireSriwastva, Mukesh KumarHensvold, AaseSrouji, SamerHentges, RochelleSt Pierre, YvesHerbolsheimer, FlorianStaats, KevinHeredero-Bermejo, IreneStabell, Alex C.Hermans, CedricStack, Brendan C., Jr.Hermida Prieto, ManuelStaderini, EdoardoHermida, Jean SylvainStadlbauer-Köllner, VanessaHernández Boluda, Juan CarlosStadnicki, AntoniHernández, ÁngelStaerman, FredericHernández-Andrade, EdgarStafford, Francis WilliamHernandez-Tejada, MelbaStafford, JamesHernández-Torres, FranciscoStagg, Brian C.Hernandez-Vaquero, DanielStagnaro-Green, AlexHerold, FabianStål, PerHerold, JörgStalder, Mark W.Herold, NikolasStallings-Smith, SericeaHeron, Courtney ElizabethStanciu, Luminita A.Herr, Daniel L.Stanek, AgataHerraiz, IgnacioStanghellini, VincenzoHerrera-Gómez, FranciscoStangl, Bethany L.Herring, NeilStanirowski, Paweł JanHerring, SusanStanojevic, Dragana M.Herrmann, Thomas R. W.Stansbury, RobertHerschbach, PeterStaples, LaurenHerten, MonikaStarace, MichelaHertz, Marla I.Starbuck, ChelseaHerzberg, Daniel L.Stark, Felicity C.Herzog, DavidStarostik, PetrHeslegrave, AmandaStasiowski, Michał JanHess, CorneliusStaskin, DavidHeß, JochenStathopoulos, GeorgiosHesse, MortenStauber, TobiasHestbaek, LiseStaud, RolandHettich, GeorgStaudacher, DawidHétu, SébastienStaudacher, Jonas JaromirHeusdens, Christiaan H. W.Stauss, Harald M.Heussler, HoneyStavinoha, Peter L.Hew-Butler, TamaraStavropoulos, Nikolaos A.Hewer, WalterSteardo, LucaHey, JeremiasSteca, PatriziaHeyse, AlexStecco, AlessandroHibbard, PaulSteels, ElizabethHibbert, BenjaminSteenbakkers, Roel J. H. M.Hida, TokimasaStefanaki, CharikleiaHidalgo-García, CésarStefanescu, CarmenHideo, WadaStefano, MasieroHiebert, LindaStefanski, HeatherHieda, MichinariSteffen, ArminHigaki, KatsumiSteffi, ChrisHiggins Neyland, MaryStefura, TomaszHighton, Patrick JamesSteiger, Hans-JakobHiguchi, SatoshiStein, Mark A.Hilhorst, MarcStein, Paul D.Hill, Lisa J.Steinbach, RobertHill, Matthew J.Steinberg, ElyHillen, ThomasSteinberg, Martin H.Hillengass, JensSteinemann, Daniel C.Hill-Kapturczak, NathalieSteinhoff, AnnekatrinHillman, Sara L.Steinmetz, SylvainHill-Yardin, Elisa L.Stell, BillHiltermann, T. Jeroen N.Stella, Giulia M.Hiltunen, Timo P.Stellos, KonstantinosHimanen, Sari-LeenaStelmach-Mardas, MartaHimmerich, HubertusStenberg, ErikHimpens, JacquesStengaard, CarstenHinchcliff, MoniqueStengel, AndreasHingorani, DinaStensballe, JakobHingorani, RittuStephan, DominiqueHinkelbein, JochenStephen, AbishHintermann, BeatStephens, JaclynHioki, HirofumiStepien, Karolina M.Hirai, TakashiStergios, KonstantinosHirani, RenaStergiou, George S.Hirano, TsunahikoSterkenburg, Anthe SuzanHiraoka, SakikoSterns, Richard H.Hirase, TakashiStetler-Stevenson, William G.Hirayama, KenjiStevens, Hieronymus P. J. D.Hirbe, Angela C.Stevens, Peter M.Hirono, KeiichiStevens, RebekahHiroshi, UreshinoStevenson, WilliamHirota, KiichiStewart, AnneHirsch, Mark A.Stewart, JayHirten, Robert P.Stewart, Jay M.Hirtler, LenaStewart, KirstyHirtz, CristopheStieger, BrunoHisada, YoheiStiles, AshleeHisako, YoshidaStiller, Carl-OlavHisamatsu, TadakazuStillman, ChelseaHishimoto, AkitoyoStingeni, LucaHitos, KerryStingl, GeorgHiyama, AkihikoStobdan, TseringHizel, CandanStocco, ChiaraHizoh, IstvanStocker, Claire J.Ho, Chiu-MingStoebe, StephanHo, JasmineStoffman, JaysonHo, NamkoongStokkeland, KnutHo, PrahladStöllberger, ClaudiaHo, ThanhStolz, Andrew AbbaHo, WinsonStonecipher, Karl G.Hoang, Thanh D.Storck, MartinHöbaus, ClemensStorsberg, JoachimHobbs, StephenStraccia, PatriziaHobson, SebastianStradner, Martin HelmutHocking, KyleStrange, CharlieHodaie, MojganStratmann, BerndHodax, JonathanStrauss, VictoriaHoellwarth, JasonStrawbridge, BecciHofer, IraStreckfuss-Bömeke, KatrinHofer, Matthias D.Streutker, Catherine JoanneHofer, MichaëlStrnad, PavelHoff, PaulaStröck, VivekaHoffman, Hunter G.Strohbach, AnneHoffmann, Michael B.Strøm, ThomasHoffmann, Michele J.Stromberg, ZacharyHofman, PaulStrong, Cristina De GuzmanHohensinner, PhilippStronge, PaulHolbrook, Eric H.Struglics, AndréHołda, Mateusz KrystianStruhal, WalterHolien, JessicaStruyf, SofieHolingue, CalliopeStubbs, EvanHolland, NeginStuckenschneider, TimHolland, ThomasStumpfe, Florian M.Hollenbeck, ScottStutz, BernardoHolliday, RichardStyczyński, JanHollmann, Markus W.Su, Chun-HungHolloway, GrahamSu, Edwin P.Holm, IngerSu, RuiHolm, Jesper GrønlundSuami, HirooHolman, NaomiSuárez García, InésHolmes, Clifton J.Suarez-Carmona, MeggyHolmes, S. A.Suarez-Cunqueiro, María MercedesHolmstrup, MichaelSuárez-Ibarrola, RodrigoHolodinsky, JessalynSuarez-Mantilla, BrianHolsinger, DamainSubbaraman, Meenakshi S.Holt, DarcySubbiah, RameshHolt, James D.Subhi, YousifHolt, StephenSubramanian, Manju L.Holvik, KristinSubramanian, Prem S.Holvoet, PaulSubramanian, VeedamaliHolwerda, Seth W.Suciu, Bogdan AndreiHoly, RichardSuda, KenjiHolzapfel, Boris MichaelSuen, Jacky Y.Holzman, RobertSugama, JunkoHomma, YasuhiroSugawara, YasuhikoHomman-Ludiye, JihaneSugaya, NagisaHong, ChangwanSugimoto, MasahikoHong, Chien-TaiSugimoto, MasahiroHong, Hyun SookSugimoto, MitsushigeHong, JamesSugishita, YodoHong, Ji YoungSugiyama, AtsuhikoHong, Tae HoSugiyama, KemmyoHongo, TakashiSugiyama, TetsuyaHonig, Lawrence S.Suh, Chang-HeeHonigmann, PhilippSuh, Dong InHonrao, ChandrashekharSuhr, FrankHonvo, GermainSui, YongjunHoogeveen, Ron C.Sukegawa, ShintaroHooker, Angelo B.Sukhal, ShashvatHool, Livia C.Sukhovershin, RomanHooper, GarySuki, Wadi N.Hoorntje, AlexanderSukumar, PiruthiviHopp, RussellSuliman, Hagir B.Horai, YoshiroSullivan, A. DavidHorgusluoglu, EmrinSullivan, Matthew J.Hori, SatoshiSultan, Ahmed S.Horibe, MasayasuSultan, Laith R.Horie, HisanagaSummer, RossHoriuchi, SatoshiSumner, Anne E.Horiuchi, TetsuyaSun, ChengwenHorne, Vincent E.Sun, ChongkuiHorny, MichalSun, JunjiangHorowitz, GiladSun, KunfengHorowitz, JamesSun, Lisa R.Horstkorte, RüdigerSun, WeiHorstmann, MarcusSun, XiaofeiHortobagyi, TiborSun, XiaolunHorton, JohnSun, XinghuiHorton, Jonathan C.Sun, XingminHorvatits, ThomasSun, YapingHoseini-Yazdi, HoseinSun, YeHoshina, TakayukiSun, ZhengwangHosohata, KeikoSun, ZhonghuaHossain, Mohammad ZakirSunakawa, YuHossain-Ibrahim, KismetSundaramoorthy, PasupathiHothi, HarrySundling, VibekeHöti, NaseruddinSung, Yon K.Hotta, YoshihiroSung, Yoon-KyoungHotter, GeorginaSunjaya, Anthony PauloHouchens, Nathan W.Supervía, MartaHoughton, DamonSuppan, LaurentHoughton, MichaelSurber, ChristianHoushdaran, SaharSurdacka, AnnaHouzé, SandrineSurdacki, AndrzejHove, Jens DahlgaardSuren, ChristianHoward, Ileana M.Surendran, SushithaHowden, SaraSureshkumar, Kalathil K.Howes, Laurence GuySurguchov, AndreiHoxha, ElionSuryadevara, VidyaniHoye, AngelaSuryavanshi, SantoshHoying, James B.Susami, TakafumiHoyos, CamillaSuttle, Catherine M.Hozumi, HironaoSutton, JillHsiao, Sheng-MouSuttorp, NorbertHsieh, MichaelSuwan, KeittisakHsu, FrankSuzuki, AyakoHsu, Kevin JSuzuki, HiromiHsu, Shu-MinSuzuki, KeisukeHsu, Te-YaoSuzuki, MaikoHtay, ThweSuzuki, MotofumiHu, BaoliSuzuki, ShinichiHu, YanmeiSuzuki, ShinyaHu, YingSuzuki, SusumuHu, YinlingSuzuki, YoriyasuHuang, G. Khai LinSuzuki-Kerr, HarunaHuang, George T.-J.Svaco, MarkoHuang, Henry D.Svardsudd, KurtHuang, JianshengSvendsen, Claus BoHuang, JijunSviggum, Hans P.Huang, KaiSvirsky, MarioHuang, LeiSwami, UmangHuang, LongshuangSwamy, GaneshHuang, TimothySwanström, Lee L.Huang, XiaobinSwaroop, AnandHuang, YongzhiSwarup, IshaanHübel, ChristopherSweatman, W. MarkHuber, KurtSweeney, TimothyHuber, ReneSweeting, Arianne N.Hubková, BeátaSwegen, AleonaHudlikar, RasikaSwenne, Cees A.Hudson, GeoffreySwenson, Cynthia CupitHuebner, HannaŚwiątkiewicz, IwonaHueso, MiguelŚwiderek-Matysiak, MariolaHugelshofer, MichaelSwijnenburg, R. J.Hughes, Christopher V.Swistel, Daniel G.Hughes, David T.Świtońska, MilenaHughes, JenniferSyan, RaveenHuh, Joo YoungSyed, Johar R.Huhn, RagnarSygit, KatarzynaHuling, JenniferSymmank, JuditHull, ChrisSymoens, SofieHull, LouiseSymonds, Michael E.Hüls, AnkeSyn, Wing-KinHulsebosch, Claire E.Sytnyk, VladimirHultcrantz, RolfSyx, DelfienHultdin, JohanSzade, KrzysztofHultin, MagnusSzakmany, TamasHultqvist, JennySzamotulska, KatarzynaHumenberger, MichaelSzczepanek, JoannaHumpel, ChristianSzczepankiewicz, BarbaraHumrich, Jens Y.Szczepanski, ArturHung, KennethSzczepek, Agnieszka J.Hung, Li-FangSzczerbinski, LukaszHunt, Beverley JaneSzeliga, JacekHunt, William R.Szer, JeffHunter, Elizabeth G.Szetpizeowski, JacekHuotari, MattiSzewczuk, Myron R.Hupin, DavidSzewczyk, KatarzynaHuppler, Anna R.Szkodziak, Piotr RobertHurford, WilliamSzmuda, TomaszHurtado-Fernández, ElenaSzodoray, PeterHurtubise, KarenSzóstek-Mioduchowska, AnnaHusby, Paul J. A.Sztanke, KrzysztofHuselstein, CélineSzulc, AgataHussain, MulazimSzychowski, JefferyHussain, RashadSzylińska, AleksandraHussain, Sultana MoniraSzyluk, Karol J.Husson, OlgaSzymczak, AleksandraHutt, AxelSzyszkowicz, MieczyslawHuttner, InkenSzyszkowicz, MieczysławHutton, George JosephTabatabaei-Jafari, HosseinHuynh-Do, UyenTacke, FrankHwang, Hun-GyuTada, NaoyaHwang, Hyeon SeokTada, YukoHwang, Jae-JoonTadmor, TamarHwang, Ki-ChulTadros, MichealHwang, MarkTaefehshokr, NimaHwang, Seung RimTafuri, DomenicoHwang, Yong IlTaga, ArensHwu, Ruth S.Tagarakis, GeorgiosHyatt, HaydenTagawa, TsuyoshiHyer, SteveTaghizadeh, HosseinHyink, Deborah PinsonTagliamento, MarcoHynnekleiv, TorfinnTAGUCHI, KENSEIHytti, MariaTahvildari , MaryamIaccarino, GuidoTaiar, RedhaIaccino, EnricoTaishi, NakamuraIacono, PierluigiTakabatake, KiyofumiIacopi, ElisabettaTakagi, ToshioIaculli, FlaviaTakahara, MitsuyoshiIaniro, GianlucaTakahari, DaisukeIannaccone, AndreaTakahashi, HidenoriIannuzzi, ClaraTakahashi, JunIasevoli, FeliceTakahashi, KenIatí, GiuseppeTakahashi, MamoruIbáñez-Costa, AlejandroTakahashi, NaokiIchibori, YasuhiroTakahashi, ToshifumiIchida, FukikoTakahashi, YusukeIchikawa, HajimeTakahiro, WadaIchikawa, TetsuoTakai, HidekiIckmans, KellyTakamaru, HiroyukiIdicula, Titto TTakamatsu, YasuyukiIdolazzi, LucaTakami, AkiyoshiIdrovo, Juan-PabloTakami, TaroIerardi, EnzoTakao, MasatoIfantides, CristosTakasawa, KenIga, JunichiTaked, TakayukiIglesias-Osma, Maria CarmenTakedatsu, HidetoshiIglseder, BernhardTakegahara, NorikoIgnee, AndreTakehara, KimieIgnjatovic, VeraTakei, ReotoIgoe, AnnTakemoto, JodyIhemelandu, ChukwuemekaTakenaka, MamoruIijima, KazutoshiTakeoka, MasanoriIijima, TakehikoTakeuchi, HisashiIjare, Omkar B.Takeuchi, KazuhikoIkada, YoshitoTakura, TomoyukiIkeda, KeigoTalati, ArdesheerIkeda, YoukoTalento, AlidaIkeda, YukiTallman, Dina A.Ikegaya, NaokiTambaro, FrancescoIkezawa, KenjiTambuwala, MurtazaIkuta, ToshikazuTamkovich, SvetlanaIlatovskaya, DariaTamma, RobertoIldefonso, Cristhian J.Tampe, DésiréeIliadi, AnnaTamura, YasuhisaIliescu, Cezar A.Tan, Ai LynIliou, Marie ChristineTan, Bee K.Illek, BeateTan, JoanneIlluminati, GiulioTan, LeeIlves, OutiTan, Toh LeongImai, ChihayaTan, WenbinImai, HisaoTan, YutingImai, YoichiTana, ClaudioImamura, MasahiroTanabe, NaoyaImamura, TeruhikoTanaka, AkihitoImamura, YoshikiTanaka, AtsushiImataki, OsamuTanaka, HiroshiImbert, VéroniqueTanaka, Hiroyoshi Y.Imbery, Terence A.Tanaka, HiroyukiImmins, TikkiTanaka, IchidaiImpellizzeri, AlessandraTanaka, YasuhitoImpellizzeri, PietroTanaka, YoshikazuInaki, NoriyukiTanbo, TomInama, MarcoTanda, Maria LauraInamo, YasujiTang, BenjaminInbar-Feigenberg, MichalTang, ChrisInder, Maree LTang, NingInder, Warrick J.Tang, Peter H.Indino, CristianTang, ShiyuIndolfi, CiroTang, ThomasIndraccolo, UgoTangl, StefanInfante, MarcoTanigawa, TakeshiInfante-Cossio, PedroTaniguchi, HiroakiIng, Steven W.Tani-Ishii, NobuyukiIngendoh-Tsakmakidis, AlexandraTanini, DamianoIngraham, Christopher R.Tanini, MarcoInnocent, GilesTanino, YoshinoriInohara, TakuTaniyama, YusukeInoue, JunTanizaki, ShinsukeInoue, JunichiroTanner, MichaelInoue, TakamitsuTanner, TarjaInserte, JavierTanoubi, IssamIntra, Francesca SangiulianoTan-Showyin, JackieInvernizzi, MarcoTansley, Sarah L.Inwads-Breland, DavidTao, ChenqiIoannou, LeonidasTao, Ya-XiongIoannou, PetrosTapper, ElliotIobst, ChristopherTaradaj, JakubIocca, OresteTarannum, MubinIommelli, FrancescaTarantini, GiuseppeIonescu, Mihaela IleanaTarantino, GiovanniIonnadis, ArgyriosTarantino, UmbertoIonta, SilvioTaranto-Montemurro, LuigiIorio, VincenzoTarasov, AndreiIovane, AndreTardella, MarikaIovino, ClaudioTargońska-Stępniak, BożenaIqbal, M. AnwarTarkkila, PekkaIrani, MehraboonTarnowski, MaciejIraqi, FuadTarrant, Seth M.Ireland, Anthony J.Tarsitano, AchilleIris, FrançoisTartaglia, GianlucaIrisawa, AtsushiTartey, SarangIrisawa, HiroshiTashbayev, BehzodIrlbeck, MichaelTashima, ToshihikoIrving, Melita D.Tashiro, MitsuruIrving, Peter M.Tatullo, MarcoIrving, William L.Taurino, MaurizioIsakoff, Michael S.Tausk, Francisco A.Isaza, NataliaTavakoli, JavadIsemura, MamoruTawil, Bill J.Ishigami, JunichiTaxbro, KnutIshiguchi, HironoriTaylan, FulyaIshihara, TakayukiTaylor, JulianIshihara, ToruTaylor, KirkIshii, HidekiTaylor, Peter WilliamIshii, KenichiroTaylor, SophieIshii, MasakazuTaylor, William R.Ishii, YumikoTchah, HungwonIshikawa, NobutsuneTchivileva, Inna E.Ishiyama, HiroyukiTe Marvelde, LucIsiadinso, IjeomaTe Nijenhuis, JanIskra, MariaTeague, Shawn DIslam, Mohammad MirazulTeague, Warwick J.Islam, SorifulTecco, SimonaIsobe, MasanoriTecson, Kristen M.Isoherranen, KirsiTedesco, MarinellaIsola, GaetanoTeh, Andrew W.Itatani, YoshiroTeich, NielsItenov, Theis SkovsgaardTeissier, NatachaIto, FumitakeTejada Giráldez, Miguel A.Ito, HiromuTeles, AlissonIto, KazuhiroTelleus, Gry KjaersdamIto, ShigekiTen Berg, JurriënIto, TakamichiTen Cate, HugoItoh, YasushiTena, BeatrizItokazu, YutakaTenbrock, KlausIvanišević, AnaTendulkar, KetkiIvanova, EkaterinaTenforde, Adam S.Ivey, Malina J.Tenhunen, MirjaIvica, AnjaTennstedt, PierreIvorra, BenjaminTenore, GianlucaIwahashi, ChiharuTentolouris, NikolaosIwakoshi, ShinichiTeo, AndrewIwamoto, NaokiTeofili, LucianaIwamoto, SeanTeotia, PoojaIwamoto, ShotaroTeper, SlawomirIwano, HidetomoTerada, JiroIwano, HiroyukiTeragawa, HirokiIwasa, ToruTerai, ShujiIwasawa, ShunichiroTerentes-Printzios, DimitriosIwata, HiroshiTeresiński, GrzegorzIwata, KentaroTerlizzi, MichelaIwata, KouichiTerradas, MarionaIwona, NiedzielskaTerreni, EleonoraIyer, ShilpaTersigni, ChiaraIzawa, Kazuhiro P.Teruel-Montoya, RaúlIzydorczyk, BernardettaTerzic, MilanIzzetti, RossanaTesfaye, Wubshet H.Izzo, RaffaeleTesio, ValentinaIzzo, ValentinaTesta, AlexanderIzzy, Manhal J.Testa, GianlucaJabbour, Tony ElTesta, GiulianoJabłonowski, ZbigniewTestarelli, LucaJabłoński, Marcin JacekTestori, TizianoJablonski, Renea P.Tetè, GiuliaJablonski-Momeni, AnahitaTeteloshvili, NatoJacka, MichaelTetz, GeorgeJacob, HavjinThacker, DeepikaJacob, Helder BThaker, Youg RajJacob, Sharon E.Thakkar, PrashantJacob, SophieThakur, Basant KumarJacobs, ColinThakur, ChitraJacobs, Michelle M.Thakur, ShilpaJacobs-Cachá, ConxitaThankam, Finosh G.Jacobson, Moriah L.Tharkeshwar Raghunath, Arun KumarJácome, CristinaThavamani, AravindJacquemin, M.Thavendiranathan, PaaladineshJacquemont, SebastienTheil, Jan ChristophJadlowiec, CarolineTheilmann, LorenzJaehne, Anja KathrinThekkinkattil, Dinesh K.Jaekel, JuliaThemistocleous, MariosJaeschke, Rafał R.Then, CorneliaJafari, Mohammad H.Theocharidou, AnnaJafferany, MohammadTheodoraki, KassianiJaffrain-Rea, Marie-LiseTheorell, TöresJagadish, Pooja S.Therese, RiedemannJagga, SupriyaThiagarajan, RaviJaggavarapu, SiddharthThiele, ThomasJagielski, MateuszThielen, BethJahangiri, LeilaThielke, StephenJahr, HolgerThierry, LeblancJaillard, SylvieThillai, MuhunthanJain, DeepanshuThirumavalavan, NannanJain, DiwakarThiruvenkatarajan, VenkatesanJain, ShanuThomas, Anusha ShirwaikarJakaria, Md.Thomas, Bolaji N.Jakiel, GrzegorzThomas, PeterJakimovski, DejanThomas, Randal J.Jalali, NedaThomas, Rebecca L.Jalava, KatriThomas, Robert J.Jallad, SamerThomas, VineetJalou, HasnaaThomas, WilliamJamal-Hanjani, MariamThomasy, Sara M.James, ChloeThompson, Andrea D.James, Erica L.Thompson, BrennanJames, Rob S.Thompson, Campbell H.James, ShermanThompson, ChristopherJamesdaniel, SamsonThompson, Emily R.Jamy, Omer HassanThompson, Miles D.Janakiraman, HarinarayananThompson, NickJaneh, OmarThompson, Rebecca R.Jang, Chul HoThompson, RobinJänig, WilfridThomsen, Allan RandrupJanis, Jeffrey E.Thomsen, Jesper S.Janiszewska, MariolaThomsen, ThomasJanket, Sok-JaThomson, Neil C.Janosi, Rolf AlexanderThomson, SimonJansen, ChristianThong, Melissa S. Y.Januszek, RafałThorne, ClaireJape, GayatriThorsen, Anders LillevikJaramillo, David E.Thrower, EdwinJarnecke, Amber M.Thuesen, Anne CathrineJarrell, JohnThuillier, RaphaëlJarstfer, Michael BruceThunnissen, ErikJasdanwala, SarfarazThurm, AudreyJaskula-Sztul, RenataThürmann, PetraJaver, Amin R.Thurnheer, ThomasJavid, MahsaTian, WeihuaJavier Jiménez-Jiménez, FelixTian, YuanJavor, SanjaTiano, LucaJawad, Muhammad A.Tiboni, Gian MarioJawinski, PhilippeTicconi, CarloJayanthi, SankarTicinesi, AndreaJayappa, Kallesh D.Tick, HeatherJayaprakash, PriyamvadaTiedje, VeraJayawardena, DulariTiefenboeck, Thomas M.Jedrzejczak, WiktorTietjen, GregoryJedrzejewski, GrzegorzTietjens, MaikeJee, Yong-SeokTiganescu, AnaJeffcoate, William J.Tilevik, DianaJefferson, Jonathan A.Timmons, Mark K.Jeffery, SteveTinazzi, ElisaJeffrey, CampsenTinelli, GiovanniJeffrey, ElmendorfTiniakou, EleniJehn, UlrichTinti, FrancescaJelin, EricTisch, Stephen H. D.Jelinek, LenaTischer, ThomasJellis, ChristineTiteca-Beauport, DimitriJelski, WojciechTiziana, ManciniJeltsch, MichaelTlemsani, CamilleJenks, Christopher L.Tliba, OmarJennewein, LukasTobias Sejbaek, MathiesenJensen, Jens-UlrikTobin, KatyJensen, Kai OliverTobin, MartinJentjens, SanderTobin, StephanieJeon, Byeong HwaTocmo, RestitutoJeon, IkchanTodt, DanielJeon, Jae YongToffoli, AndreaJeon, Jin-hunTofler, GeoffreyJeon, SoheeToft-Bertelsen, Trine L.Jeong, Byeong-HoTognarelli, SeleneJeong, Seok HoonToguchida, JunyaJeong, Young-HoonTohme, Samer T.Jeppesen, Janni VikkelsøToida, ChiakiJerzy, MosiewiczTojo, NaokiJesús Gustavo, Ponce GonzálezTokarek, TomaszJewett, AnahidTokarz, Debra A.Jhun, Byung WooTokas, TheodorosJi, LingyunTokodai, KazuakiJiang, QuanTokuhashi, YasuakiJiang, ShaoningToledano, YoelJiang, XinyinTölle, AngelikaJiang, XuejuanTollenaar, Lisanne S. A.Jickling, Glen C.Toma, ClaudioJillella, DineshTomás-Aragonés, LucíaJimbow, KowichiTomaselli, VeneraJiménez Garcia, RodrigoTomasev, NenadJimenez, Patricia T.Tomatsu, ShunjiJiménez, WladimiroTomba, ElenaJiménez-Bonilla, Julio FranciscoTomescu, Mirela CleopatraJiménez-Del-Barrio, SandraTomita, YoheiJiménez-Jiménez, Félix JavierTomizawa, NobuoJiménez-Mateos, Eva MaríaTomkin, Gerald H.Jin Ho, WonTomlinson, Gail E.Jin, HongTomycz, Luke D.Jin, Hye JinTomycz, Nestor D.Jin, TaoTonetti, JeromeJing, RanTong, WeiJinnouchi, HiroyukiToniutto, PierluigiJinwal, Umesh K.Tonni, GabrieleJirapatnakul, ArtitTonoyan, LilitJneid, HaniTontini, Gian EugenioJo, Dong HyunToor, AmirJo, Sang-HoTorcia, MariaJoachimiak, EwaTorella, DanieleJoão-Pereira, MariaToriello, FilippoJochmanova, IvanaToriello, HelgaJochum, ChristophTorlinska, BarbaraJockel-Schneider, YvonneTorlone, ElisabettaJødal, LarsTorné, RamonJoffe, ErelToro, Mario DamianoJohannesson, LizaTorras, JuanJohansson, AndersTorras-Ambròs, JoanJohn, Mike T.Torre, GuglielmoJohn, Robert SneydTorre, Jorge Arias-de LaJohns, Terrance G.Torreggiani, MassimoJohnsen, JillTorregrosa, Javier FerrerJohnson, DanielTorrente-Segarra, VicençJohnson, Dylan M.Torres, Maria LeilaniJohnson, Martin K.Torres, NapoleonJohnson, PaulTorres, RuiJohnson, Thomas MTorres-Russotto, DiegoJohnson-Mann, CrystalTorres-Sánchez, IreneJohnston, Callum MichaelTorriglia, AliciaJohnston, Thomas PTorti, MargheritaJohnstone, MurrayToruner, Gokce AltayJolliffe, David A.Tosi, DavideJolly, MKToskich, BeauJoly, Berangere S.Totary-Jain, HanaJonak, KamilToth, RachelJones, AnneTóth-Molnár, EditJones, ChrisTottman, Anna CatherineJones, Corinne A.Totzeck, AndreasJones, Deryk G.Totzeck, MatthiasJones, James F. X.Touboul, CyrilJones, JenniferToulis, VasileiosJones, John K.Toumpanakis, ChristosJones, MatthewTournier, MarieJones, RobinToutounchi, SadeghJonnalagadda, Anil KumarTowbin, JeffreyJoos, ErikaTownend, JonathanJoossens, MarieToyokawa, TatsuyaJoosten, AlexandreToyomasu, YoshitakaJoosten, Koen Felix MariaToyos, MelissaJordan, ElizabethToyoshima, DaisakuJordan, JensTraboulsi, EliasJørgensen, Anders PalmstrømTrahair, TobyJosé Tauler Riera, PedroTraiffort, ÉlisabethJose, MatthewTrambas, ChristinaJose, Pedro A.Tramonti, CaterinaJose, PerezTran, HuyentranJoseph, DesalineTran, Nguyen ToanJost, DanielTran, Susan T.Jost, PhilippTran, Thai HoaJoubert, Lydia-MarieTran, Tú AnhJoubert, MadeleineTrauer, JamesJöud, AnnaTraynelis, Vincent C.Joung, Bo YoungTrejo, ImeldaJoyner, MichaelTremont-Lukats, Ivo W.Jozwiak, MathieuTrendelenburg, MartenJu, Man KiTrenti, LorisJu, TingtingTrentini, AlessandroJud, PhilippTreskatsch, SaschaJue, Joshua S.Trevillyan, JanineJuffermans, NicoleTrevisan, CaterinaJuhn, AlexanderTrifirò, GianlucaJulián, EstherTriggiani, MassimoJulius, UlrichTrikalinos, NikolaosJulkunen, IlkkaTrimaille, AntoninJull, AndrewTrimarco, BrunoJumean, Marwan F.Trimboli, PierpaoloJun, Jae-BumTrimigno, AlessiaJun, Se-RanTriolo, GiacintoJuncos-Rabadán, OnésimoTripathi, Jitendra KumarJung, ChristianeTriplett, R. GilbertJung, Hwi-DongTripodi, DomenicoJung, Jin-HwaTriponez, FredericJung, JinseiTriposkiadis, FilipposJung, JiyeonTrippel, Tobias DanielJung, Ki JinTrisolino, GiovanniJung, Kyoung InTritapepe, LuigiJung, Se YongTriunfo, StefaniaJung, Se-YounTrivanovic, DrenkaJung, SungmokTrivedi, DarshanJung, Yuh-SeogTrohman, Richard G.Junglee, Naushad AliTrojano, GiuseppeJurczak, AnnaTrojsi, FrancescaJustice, Jamie N.Tronnier, Volker MartinKaburaki, ToshikatsuTroost, JonathanKachadourian, Lorig K.Troyer, RyanKachrimanidou, MelinaTruchetet, Marie-ÉliseKacperczyk-Bartnik, JoannaTrudel-Fitzgerald, ClaudiaKaczmarek, KrzysztofTruong, UyenKadambi, Pradeep V.Trybek, PaulinaKadonosono, KazuakiTsai, Han-MouKaesmann, LukasTsai, JamesKafadar, KarenTsai, Ming-HorngKahn, Nicolas CarlosTsai, TeresaKai, HirofumiTsakiridis, IoannisKai, KeitaTsakiris, Dimitrios A.Kaindl, AngelaTsalouchos, ArisKaiser, ThorstenTsapogas, PanagiotisKajiyama, HiroshiTsarouhas, KonstantinosKakeji, YoshihiroTsatsakis, AristidisKakinuma, YoshihikoTscharre, MaximilianKakizawa, YukinariTse, Chung SangKalafat, ErkanTsekoura, MariaKalasauskas, DariusTseng, EvaKalaska, BartlomiejTsikouras, PanagiotisKalinina, OlgaTsinopoulos, IoannisKalinowski, JolaadeTsitlakidis, StefanosKaliszewski, KrzysztofTsitsilonis, Ourania E.Kalladka, MythiliTso, For YueKallen, KarinTsolakis, Apostolos V.Kalogeropoulos, AndreasTsouh Fokou, Patrick ValereKalogiannidis, Ioannis A.Tsoukalas, DimitrisKalpadakis, ChristinaTsoulfas, GeorgiosKalsi, TaniaTsuboi, TomomasaKamal, HarisTsubota, AkihitoKameyama, AtsushiTsugawa, HitoshiKamijo, KeitaTSUJI, DaikiKamimura, HiroteruTsuji, ShojiKamiński, KarolTsuji, YousukeKamiński, Tomasz W.Tsukahara, HirokazuKaminsky, David A.Tsukahara, KengoKamiya, TakeshiTsung, AllanKammerlander, AndreasTsur, Avishai MichaelKamp, KendraTsushima, HidetoshiKamp-Becker, IngeTsuzuki, KenzoKampen, UweTuccari, GiovanniKampmann, UllaTucci, MarcoKampolis, Christos F.Tucker, LarryKanaka-Gantenbein, ChristinaTuddenham, Edward G. D.Kanaporis, GiedriusTuinder, Stefania M. H.Kanaujiya, JitendraTumedei, MargheritaKanavakis, GeorgiosTumilty, SteveKanclerz, PiotrTumini, StefanoKanczler, JanosTumscitz, CarloKanda, TatsuoTünde, TarrKandasamy, SanjivanTunduguru, RagadeepthiKandpal, ManojTunon, JoseKane, EleanorTuomilehto, JaakoKaneko, SyuzoTurajlic, SamraKang, Hee-GyooTural, ÜmitKang, Hee-TaikTurco, LaurenKang, JeehoonTurcotte, Lucie M.Kang, Jeong-HyunTurek, Paul J.Kang, Ji YoungTuretta, MatteoKang, Ju-HeeTuri, StefanoKang, KyonghwaTurnbull, Marion T.Kang, Kyung LhiTurner, Alice MKang, Nyeonju (NJ)Turnquist, CasmirKang, Yoon-JungTurri, MaraKang, YuTurturice, Benjamin ArthurKanno, ToruTutino, Vincent M.Kanno, YuichiroTuttolomondo, AntoninoKansal, VinayTuynman, Jurriaan B.Kanungo, ShibaniTvrdik, PetrKanwar, AnubhavTwilt, MarinkaKanzaki, GoTwomey, PatKanzaki, HiroyukiTyler, Patrick D.Kanzaki, RyuTysarowski, AndrzejKanzaki, ShoTzaridis, SimoneKao, IminTzekov, RadouilKao, PeterTzortzi, Anna S.Kapan, AliUccelli, LiciaKapellos, Theodore S.Uccelli, RaffaellaKaplan, Lewis JUcciferri, ClaudioKapoor, PrashantUchida, HajimeKapritsou, MariaUchida, KeikoKapsogeorgou, E. K.Uchino, HarutoKapur, NeerajUchiyama, KojiKapur, PayalUddin, RiazKapur, RickUebanso, TakashiKaraduta, OlegUeda, KaoriKarafin, MatthewUehara, MasashiKaragiannakis, DimitriosUehleke, BernhardKarakaidos, PanagiotisUemasu, KiyoshiKarakiewicz, PierreUetake, HiroyukiKarakula-Juchnowicz, HannaUgrankar, RupaliKaralis, Dean G.Uh, YoungKaram, BoutrosUhelski, Megan L.Karaman, SinemUhl, EberhardKarantanos, TheodorosUhlig, HolmKarapanayiotides, TheodoreUhm, Jae-SunKarasik, DavidUkleja-Sokołowska, NataliaKaratas, BerfinUlanja, Mark B.Karatolios, KonstantinosUlivi, PaolaKarim, JahangirUlivieri, CristinaKarim, ShahidUlke, ChristineKario, KazuomiUlrich, Jorge Luis MéndezKarkhanis, AnushreeUmeda, KatsutsuguKarkowska-Kuleta, JustynaUmemura, NaokiKarl, MatthiasUmgelter, AndreasKarl, TimUmmarino, SimoneKarligkiotis, ApostolosUndas, AnettaKarlinger, KingaUnger, ElizabethKarm, Myong-HwanUngerstedt, Johanna S.Karnatovskaia, Lioudmila V.Ungurianu, AncaKarolewski, KazimierzUnni, Arun M.Karoor, VijayaUnno, MichiakiKarska-Basta, IzabellaUnsworth, AmandaKarst, SonjaUpadhyay, KiranKasai, TakatoshiUpadhyaya, JasbirKaschina, ElenaUppugunduri, Chakradhara RaoKashiouris, MarkosUpshaw, Jenica N.Kashiwagi, ManabuUranga, JoseKashou, AnthonyUrban, BeataKaski, Juan CarlosUrbanek, TomaszKaski, Juan PabloUrbaniak, AlicjaKasperczyk, SławomirUrbano, Ana MargaridaKaspi, EliseUrick, Mary EllenKasprzak, JaroslawUrra, XabierKassiri, ZamanehUrsic-Bedoya, JoséKassubek, JanUsategui-Martín, RicardoKasten, Mary J.Usuelli, FedericoKasumov, TakharUsui, AkihikoKatagiri, HiroshiUsui, YoshihikoKataoka, HiroshiUtley, AdamKataoka, YokoUziębło-Życzkowska, BeataKataoka, YuVaajanen, AnuKato, HideoVacchiano, VeriaKato, JoVach, KirstinKato, KatsuhikoVadakara, JosephKato, KenVagge, AldoKato, MasaruVagnildhaug, Ola MagneKato, MotoyasuVaičiūnienė, RūtaKato, SoVaidya, RahulKato, TakamitsuVaiman, DanielKato, TakaoVaira, BernardinoKatodritou, EiriniVaisman, BorisKatoh, HiroyukiVaismoradi, MojtabaKatsagoni, Christina N.Vakrou, StylianiKatsanis, EmmanuelVakulin, AndrewKatsavos, SerafeimValdepenas, BenitoKatsuyama, TakayukiValderrabano, VictorKatta, NiteshValdes, GilmerKattoor, Ajoe JohnValdés-Olmos, RenatoKatus, HugoValdivielso, Jose ManuelKatyal, SachinValdivielso, PedroKatz, Ben Z.Vale, Filipa F.Katz, JasonVale, NunoKau, Chung HowValente, JoanaKauke, MartinValente, MariarosariaKaur, GurvinderValente, Nicola A.Kaur, ManinderValente, Nicola AlbertoKaurani, LalitValenti, PieraKauric-Klein, ZoricaValentinčič, Nataša VidovičKaushik, ShellyValentine, Rudy J.Kawabata, HideakiValentiner, UrsulaKawabori, MasahitoValera-Garrido, FermínKawaguchi, NanakoValerio, AdrianoKawai, MarikoValettas, NicholasKawai, ShinichiValiño-Rivas, LaraKawai, ToshiyukiValipour, ArschangKawakami, AkinoriVallabhajosyula, SaraschandraKawakami, HisatoValli-Pulaski, HannaKawamoto, ShinichiroVallvé-Juanico, JúliaKawanami, DaijiValmaggia, Lucia R.Kawano, SatoshiValmaseda-Castellõn, EduardKawano, YawaraValmasoni, MicheleKawasaki, RyoValstar, Matthijs H.Kawata, KazumiValverde, ClaudiaKawczynski, AdamVan ’t Hul, AlexKawecki, DamianVan Alfen-van Der Velden, JanielleKay, JasonVan Beekhuizen, Heleen J.Kay, SimonVan Bodegraven, Ad A.Kayani, Waleed TVan Bortel, LucKaye, ElizabethVan Bussel, Bas C. T.Kazui, ToshinobuVan Caam, ArjanKearney, StephanieVan De Bruaene, AlexanderKeaver, Laura M.Van De Graaf, StanKedia, SatishVan Den Akker, GuusKeeling, AndrewVan Den Anker, JohannesKeenan, Robert T.Van Den Berg, BernardKeeton, Adam B.Van Den Berge, M.Keidar, ZoharVan Den Bergh, W. M.Keisaku, SatoVan Den Bogaard, EllenKejík, ZdeněkVan Den Brink, Floris S.Kekecs, ZoltanVan Den Meiracker, Anton H.Kelada, LaurenVan Der Bent, M. LeontienKelle, SebastianVan Der Doef, Hubert P. J.Keller, Jesse J.Van Der Geest, Kornelis S. M.Keller, MonikaVan Der Hage, Jos A.Kellett, John G.Van Der Heijden, JeroenKelly, Caleb J.Van Der Hoorn, Marie Louise P.Kelly, ClareVan Der Jagt, MathieuKelly, Maryellen S.Van Der Kuy, HugoKelsey, MeganVan Der Meer, Pieter F.Kelstrup, LouiseVan Der Merwe, JohannesKemler, EllenVan Der Pluijm, I.Kemmel, VéroniqueVan Der Schueren, BartKemmner, StephanVan Der Velden, JacobusKemp, EmmaVan Der Vliet, Quirine M. J.Kemp, GrahamVan Der Wekken, AnthonieKempińska-Podhorodecka, Agnieszka D.Van Der Windt, MelissaKempster, Sarah L.Van Devanter, Donald R.Kenanidis, EustathiosVan Diepen, SeanKendall, MichelleVan Dijk, J. Marc C.Kenet, GiliVan Dijkum, Elisabeth J. M.NieveenKenmochi, TakashiVan Dongen, Ivo M.Kennedy, LindseyVan Doorn, RemcoKentrup, DominikVan Dort, Christa J.Kerbage, YohanVan Dronkelaar, CarlieneKerr, BethanyVan Duijvenboden, StefanKerr, Nadine A.Van Duinkerken, EelcoKerschbaum, JuliaVan Eeden, StephanKerz, ThomasVan Eijk, MarcoKesavalu, Lakshmyya N.Van Gemert, Willemijn A. M.Kesavan, MuraliVan Gisbergen, Marike W.Keshari, SunitaVan Gool, Stefaan W.Keski-Rahkonen, PekkaVan Goudoever, HansKesselring, JürgVan Hees, StijnKessler, Alexander T.Van Hoof, Joris J.Kessler, AndreasVan Kempen, LeonKessler, DorothyVan Kimmenade, Roland R. J.Kessner, Simon S.Van Lanen, StevenKesterson, Joshua P.Van Lankveld, WimKetter, RalfVan Lieshout, EstherKevin, ZornVan Loenhout, JorisKeymeulen, AnneliesVan Londen, MarcoKezic, SanjaVan Loon, Bert Jan PotterKhacho, MireilleVan Loon, Fredericus H. J.Khaddaj Mallat, RayanVan Ochten, John M.Khakoo, ShelizeVan Oort, PoulineKhalaf, FatimahVan Oosterom, Matthias N.Khalaileh, AbedVan Rhee, FritsKhalifa, MahmoudVan Riet-Nales, Diana A.Khalil, AndréVan Rosendael, Alexander R.Khan, Mohd ParvezVan Royen, Barend JKhan, Muhammad AhmedVan Saen, DorienKhan, UzmaVan Setten, Gysbert BothoKhanna, Amber D.Van Stavern, Gregory P.Khashayar, PatriciaVan Tienhoven, GeertjanKhawaja, Imran T.Van Tilborg, MirjamKhella, HebaVan Trijffel, EmielKhemani, Robinder G.Van Veldhuisen, Dirk JanKhettab, MohamedVan Veldhuisen, EranKhlopas, AntonVan Weyenbergh, JohanKho, Alvin T.Van Wingerden, Jan PaulKhochbin, SaadiVan Zyl, MartinKhodamoradi, KajalVanacore, NicolaKhokhar, BilalVanakker, Olivier M.Kholmukhamedov, AndalebVanatta, JasonKholová, IvanaVancheri, FedericoKhong, Jwu JinVandamme, TimonKhor, YetVandenbriele, ChristopheKhor, Yet HongVandenBussche, Christopher J.Khorgami, ZhamakVandeputte, JoanKhoshpouri, PegahVandooren, JenniferKhouzam, Rami N.Vandroux, DavidKhraiche, MassoudVanni, DanieleKhurana, NamrataVannucchi, Maria-GiulianaKia, Seyed MostafaVanoni, SimoneKiciński, PawełVanwolleghem, ThomasKiely, Patrick D. W.Vaporidi, KaterinaKierkegaard, MarieVarady, Nathan H.Kikkert, WouterVarela, GonzaloKikuchi, MasahiroVarela, LuisKikuchi, SatoruVarga, Andrew W.Kikuchi, ShogoVarga, GergelyKikuta, KazutakaVarga, Matthew G.Kikuyama, MasatakaVargas, Ashley J.Kilian, CarolinVarghese, Alex C.Kilty, Shaun J.Varghese, MathewsKim, Bong ChulVargo, MaryKim, Boo-YoungVarma, NirajKim, BrianVarpula, MarjutKim, Byung-WookVarrassi, GiustinoKim, Chan YunVarricchio, EttoreKim, Chang HunVarshavsky, JuliaKim, Dae WonVartak, AbhishekKim, DasomVarughese, Kottayil I.Kim, Dong JoonVasaitis, LilianKim, Dong YoonVasconcelos, Ana Carina NogueiraKim, Du HwanVasconcelos, Manuel PestanaKim, Eung DonVasdev, NikhilKim, Gi BeomVashist, AseemKim, Grace Hyun J.Vasilescu, CatalinKim, Hack-LyoungVasilopoulou, ElisavetKim, Ha-NeuiVaškelytė, JolantaKim, Harry K. W.Vasquez, Karen M.Kim, HonghyokVass, ClemensKim, Hwa-jungVassallo, JosanneKim, Hyeun SungVassetzky, Yegor S.Kim, Hyo SongVassilakopoulos, TheodorosKim, Hyo YeolVassiliou, Aliki GeorgiaKim, Hyoun (Andrew)Vasudevan, SreelakshmiKim, Hyun KooVasuri, FrancescoKim, Hyun WooVatanen, TommiKim, HyungjinVatopoulos, AlkiviadisKim, Hyunjae RVatsayan, AnantKim, Hyun-seokVaughan, AnnaliciaKim, Jae HuiVavalle, John P.Kim, Jae-gyuVavasour, Irene M.Kim, Jae-HongVavourakis, VasileiosKim, Jae-JinVäyrynen, Sara A.Kim, Jee HwanVázquez-Borrego, Mari C.Kim, Jee TaekVeatch, Olivia J.Kim, Jennifer A.Vecchio, DavideKim, Jeong-YeonVechetti, IvanKim, JieunVediappan, Rajan S.Kim, Jin-SeokVeedu, JaneeshKim, Jin-WooVeelken, RolandKim, Joon MoVega, AlmudenaKim, Joung SooVega-Rubín-De-Celis, SilviaKim, June-SungVehreschild, Maria J. G. T.Kim, JunghoonVeit-Rubin, NikolausKim, Kang HoVekrellis, KostasKim, KijinVelasco, Carlos E.Kim, KisookVelasco, InésKim, Kye HunVelier, MélanieKim, Kyung MinVelikova, TsvetelinaKim, Lenise JiheVella, VeronicaKim, LeoVellante, FedericaKim, Mi NaVellecco, ValentinaKim, MinVemula, HarikaKim, Mi-NaVenetsanos, DimitriosKim, Min-HeeVenkataraman, Abinaya PriyaKim, NamheeVenner, Christopher PaulKim, NamjuVentura, LuigiKim, NayoungVenuthurupalli, Sree K.Kim, Ok-KyungVera Sempere, Fco JoseKim, Sang HunVerbraak, FrankKim, Sang WanVerdelis, KonstantinosKim, Seonghun HoonVerdina, TommasoKim, Seon-OkVerdú, JoséKim, SeungtaekVerdugo-Paiva, FranciscaKim, Sin-youngVergara, DanieleKim, So-YounVergara, M. NataliaKim, Su JinVergara, RubenKim, Sun WoongVerguts, JasperKim, Sung EunVerhoeven, AdrieKim, Sung GyunVerhoeven, FrankKim, Sung HuhnVerma, NupurKim, SunilVerma, PriyankaKim, Tae IlVermeulen, NathalieKim, Tae KonVerna, GiulioKim, Tae-BumVernero, MartaKim, Tae-WooVerschakelen, JohnyKim, TaisunVerschuren, Emmy W.Kim, TeayounVervaeke, StijnKim, WangdoVervoort, GeraldKim, WoojinVerweij, KarinKim, YemiVescio, AndreaKim, YerimVetrano, MarioKim, Yong HoonVetrugno, LuigiKim, Yong HwyVetter, SvenKim, Yong-GilVetto, John T.Kim, Yong-GooVeyer, DavidKim, Yoon-ChulViazis, NikosKim, Young RanViazzi, FrancescaKim, Young SeokVicenzi, MarcoKim, Young-NamVidal, Pia M.Kim, Young-TaekVider, JelenaKim, Yun-HeeVidot, HelenKimber-Trojnar, ŻanetaVieira Baptista, PedroKimura, HidehitoVielgut, InesKimura, NorikoVielsmeier, VeronikaKimura, ToshikazuViennot, StéphaneKinami, ShinichiVienola, Kari V.Kinard, BrianViers, Boyd R.Kindel, TammyVieth, MichalKing, CaraVieyra, Jorge ParedesKingsley, KarlViganò, MauroKinoshita, RyoVigne, JonathanKinoshita, TakahiroVignes, StéphaneKinoshita, TomomariVignini, AriannaKintzelé, LaurentVignon, MargueriteKipar, AnjiVijayakumar, SarathKirino, YoheiVik, TennleyKirsch, Alexander H.Vikhorev, PetrKirsch, ThorstenVilela, Eduardo M.Kirschneck, ChristianVille, YvesKiselev, Valerij GennadevicVinai, PiergiuseppeKishida, Kenneth T.Vinall-Collier, Karen A.Kishimoto, DaigaVincent, Jean-LouisKisiel, John B.Vincitorio, FrancescaKislitsina, OlgaVindigni, VincenzoKisor, David F.Vingolo, Enzo MariaKiss, RobertVinturache, AngelaKisu, IoriViola, FrancescoKitagawa, SeiichiVirmani, RenuKitaguchi, YoshiyukiVirto, LeireKitahara, TadashiVirumbrales-Muñoz, MaríaKitai, TakeshiVischer, AnninaKitamura, HiroshiVischini, GisellaKitamura, MineakiVischioni, BarbaraKitazato, KaioViscido, AngeloKitsko, DennisVisconti, DanielaKitta, TakeyaVisentin, MicheleKittel-Schneider, SarahVisgauss, JuliaKitzmüller, ClaudiaVisser, FransKiuchi, ShunsukeVissers, Kris C. P.Kiviruusu, Olli H.Vissink, ArjanKlaassen, Curtis D.Viswanathan, SowmyaKlareskog, LarsVita, RobertoKlaritsch, PhilippVitacca, MicheleKlaus, KatharinaVitale, AlessandroKleczynski, PawelVitale, GiovanniKleeff, JörgVitale, Marina ConsueloKlein, IrwinVitale, MarioKlein, Janet DVittecoq, OlivierKlein, JochenVittori, AlessandroKleinlugtenbelt, Ydo VincentVivien, BenoitKleiter, IngoVizza, Carmine DarioKlemm, PhilippVladimirov, VladimirKlemp, AlexVlajnic, TatjanaKlersy, CatherineVlk, MartinKlettner, AlexaVoderholzer, UlrichKleveland, OlaVoelkl, JakobKlich, SebastianVoermans, Rogier P.Kliewer, KaraVogel, MatthiasKlim, SebastianVogel-Ciernia, AnnieKlimczak, AleksandraVohra, HunaidKlimkowska, MonikaVoican, CosminKlinge, UweVoland, RickKlingemann, HansVolarevic, VladislavKlingenberg, RolandVollbrandt, TillmanKlinger, MarianVollmer, MarcusKlinke, AnnaVon Der Thüsen, JanKlip, AmiraVon Eyben, Finn EdlerKlobuch, SebastianVon Lewinski, DirkKloesel, BenjaminVon Lilienfeld-Toal, MarieKlonizakis, MarkosVon Piekartz, HarryKlontzas, MichailVon Roth, PhilippeKlopfleisch, RobertVon Und Zu Fraunberg, MikaelKlosterhalfen, BerndVoorhees, Timothy J.Klotz, Anna-LuisaVos, Alinda G.Klughammer, JohannaVoskuil, MichielKluzek, StefanVoss, Jan OliverKnafl, DanielaVoss, UlrikkeKnapik, PiotrVouros, IoannisKneer, JonasVoutsadakis, IoannisKneijber, M. C. J. (Martin)Voutsadakis, Ioannis A.Knezevic, Nebojsa NickVoza, AntonioKnier, BenjaminVrachatis, Dimitrios A.Knight, Richard J.Vrachimis, AlexisKniha, KristianVrakas, GeorgiosKnippa, EmilyVriezekolk, Johanna (Joke) E.Knott, RachelVuckovic, IvanKnutsson, Karl AndersVuilleumier, NicolasKo, Byuk SungVujasinović, MiroslavKo, Hyun-SunVujosevic, StelaKobaek-Larsen, MortenWabnegger, AlbertKobayashi, HiroakiWachowska, MałgorzataKobayashi, HirokoWade, Charles E.Kobayashi, TetsuroWadman, Renske IKobiela, JarosławWafa, HazemKoch, MatthiasWagener, GebhardKoch, StefanWagner, DaleKochhar, RohitWagner, Nikolaus B.Kodadek, Lisa M.Wahaia, FaustinoKodama, HideyaWåhlander, HåkanKodziszewska, KatarzynaWahlin, StaffanKoehl, BérengéreWajda, AnnaKoga, HisashiWakatsuki, MasaruKoh, ByumseokWaksmundzka-Hajnos, MonikaKoh, Jae ChulWald, DavidKohga, AtsushiWaldner, MaximilianKohno, TakashiWalecka, IrenaKoie, TakuyaWalenga, Jeanine M.Koirala, RanjanWalewicz, KarolinaKoivunen, Jussi P.Walia, HarneetKojima, TsuyoshiWalker, DavidKok, WouterWalker, SteveKoka, SreenivasWalker, ValerieKokkinos, AlexanderWall, DonnaKokot, NielsWallace, Daniel J.Kokubun, KeisukeWallace, HamishKolasa-Wołosiuk, AgnieszkaWallace, KedraKolben, ThomasWallwiener, MarkusKolbenschlag, JonasWalsh, KatrinaKolch, WalterWalsh, RichardKoletas, EvangelosWalters, Eugene HaydnKoli, SwanandWalton, Brian L.Koliarakis, IoannisWalton-Moss, Benita JeanneKoliesnik, IevgenWambier, CarlosKolios, AntoniosWammes, LindaKölle, MarkusWand, SaskiaKoller, AllisonWanderer, StefanKollert, FlorianWang, Chia-SiuKolling, StefanWang, Chun-HouKollmuss, MaximilianWang, FupengKolls, Jay K.Wang, Joshua JianxinKolluru, GopiWang, JunKolodziej, MalgorzataWang, KankanKołodziejczak, MichalinaWang, LinKolonko, AureliuszWang, LiyeKoltz, Michael T.Wang, LuKomatsu, DavidWang, MengyuKomatsu, HiroakiWang, Nan-kaiKomatsu, MasanobuWang, ShaobinKombo, NinaniWang, Xin ShelleyKomenaka, Ian K.Wang, XinkunKomnenov, DraganaWang, YijieKomosinska-Vassev, KatarzynaWang, YingKompa, Andrew R.Wang, YumengKondo, TadakazuWarburton, GaryKondo, TatsuyaWard, MarkKonialis, ChristopherWard, PeterKonieczny, MariuszWard, Peter A.Konigsberg, Beau S.Ward, Roberta J.Königstein, KarstenWardlaw, JoannaKoninckx, Philippe R.Wardowska, AnnaKonishi, MasaakiWarnakulasuriya, SamanKono, MasahiroWarner, SusanneKono, YutaWarren Waks, JonathanKonopka, TomaszWarren, Cirle A.Konrad, LutzWarren, Russell F.Konrads, ChristianWarrillow, StephenKonstantakou, Eumorphia G.Warsame, RahmaKonstantinidi, AikateriniWarszawik-Hendzel, OlgaKonstantinidou, Maria KalliopiWarzecha, ZygmuntKonstantinos, TepetesWashio, KenKontos, Christos K.Wasowicz, MarcinKontovounisios, ChristosWästerlid, ToveKontro, MikaWaszczykowska, ArletaKonvalinka, AnaWaszkiewicz, NapoleonKoo, Do HoonWatanabe, AtsuyukiKoo, Ki-TaeWatanabe, EiichiKook, DanielWatanabe, KeikoKoopman, MaudWatanabe, MegumiKoos, BjörnWatanabe, MikikoKopp, SaschaWatanabe, YoshioKopsaftis, ZoeWatanabe, YuichiroKordek, AgnieszkaWatson, Anthony W.Korell, MatthiasWatson, DionysiosKorinthenberg, RudolfWatson, Hugh RobertKornblith, EricaWatt, KathleenKornblith, Lucy Z.Watt, PeterKorona-Glowniak, IzabelaWattchow, DavidKortüm, K. MartinWatz, HenrikKorzeniewski, StevenWaung, MaggieKoscielny, SvenWautier, Jean-LucKosinska-Kaczynska, KatarzynaWawrzkiewicz-Jałowiecka, AgataKosinski, PrzemyslawWax, MarkKosinsky, Robyn LauraWaxman, Aaron B.Kosior-Jarecka, EwaWa̧ż, PiotrKoska, JurajWazana, AshleyKosmala, AleksanderWdowiak, ArturKosmidis, ChrisWeatherley, NicholasKosola, JussiWeaver, Donald J., Jr.Kost, GeraldWeaver, Frances M.Kostareva, AnnaWebb, Carol F.Köstenberger, MarkusWebb, LucyKostner, Gert M.Weber, Christian DavidKoszelewski, DominikWeber, HeinrichKotanidou, AnastasiaWebster, Keith A,Kotarski, JanWeghofer, AndreaKotchoubey, BorisWegrzyn, GrzegorzKotfis, KatarzynaWei, YonglongKothari, Vishal M.Weichert, AlexanderKotloski, RobertWeigand, KilianKotsakis, GeorgeWeil, Yoram A.Kotsakis, Stathis D.Weilenmann, DanielKotsiou, Ourania S.Weimers, PetraKotula-Balak, MałgorzataWeinberg, Seth H.Kougioumtzopoulou, AndromachiWeiner, Howard L.Kouidrat, YoussefWeiner, JulianeKouji, HaradaWeinmann, KarolinaKoulen, PeterWeinstein, David A.Koupenova, MilkaWeiss, GunterKoustas, EvangelosWeiss, MarenKoutouzis, MichaelWeisz, AlessandroKoutsarnakis, ChristosWeledji, Elroy PatrickKovac, Anthony L.Welker, Martin-WalterKovacevic, AlexanderWellenberg, Ruud H. H.Kovács-Öller, TamásWelling, MickKövamees, OskarWellner, Ulrich FriedrichKovvuru, KarthikWellons, MelissaKowal, MateuszWells, JessicaKowalewski, MariuszWells, TimKowalik, ArturWelsch, ThiloKozakiewicz, MarcinWelsh, TomasKozarek, RichardWelty, FrancineKozieł, MonikaWen, JianjunKozik, Teri M,Wen, RongKraeuter, Ann KatrinWen, XiaominKraft, Timothy W.Wendelboe, Aaron M.Krahmer, FelixWenning, M.Krajewska, MagdalenaWennogle, Sara A.Krajewska-Włodarczyk, MagdalenaWenzl, RenéKrajewski, Stefanie SandraWerbel, WilliamKrakowski, PrzemysławWeres, AnetaKramer, Phillip R.Werneke, UrsulaKramer, Robert S.Wernig, FlorianKramer, TobiasWernly, BernhardKrampitz, GeoffreyWerth, Victoria P.Krantz, David S.West, JanneKrasińska, BeataWest, NicholasKrasiński, ZbigniewWest, RachelKrasniqi, EriseldWester, KnutKrassioukov, Andrei V.Westin, JohanKraus, ArminWestmark, Cara J.Kraus, Courtney L.Westwood, Mark A.Kraus, VirginiaWewalka, MarleneKrawczyk, PawełWhillier, StephneyKrawczyński Maciej, RobertWhitaker, Amanda T.Krawiec, PaulinaWhite, BarbaraKrebs, MarkusWhite, Christopher J. M. D.Krela-Kaźmierczak, IwonaWhite, KatharineKrengel, Maxine H.Whitehead, Kevin J.Kretsovali, AndronikiWhitelaw, MelissaKreuter, JustinWhiteley, Mark S.Kreyer, StefanWhitlam, JohnKribben, AndreasWhitman, Barbara Y.Krieghoff-Henning, EvaWhitman, Isaac R.Krintus, MagdalenaWhitney, Daniel G.Krishna, GokulWhitney, JulieKrishna, JyotiWiborg, OveKrithardies, LeonardWidera, MarekKroese, Frans G. M.Widhalm, Harald KurtKröger, EdeltrautWieckiewicz, MieszkoKrois, JoachimWieczorek, EdytaKrok-Schoen, JessicaWiedenroth, Christoph B.Kronberger, IrmgardWielosz, EwaKronbichler, AndreasWiers, Corinde E.Kronreif, GernotWiggins, Barbara S.Kroon, JeffreyWijdicks, Coen A.Kropp, MartinaWijeratne, TissaKroushev, AnnieWijnen, MarkKrstic, JelenaWijnholds, JanKrueger, Gerald G.Wijns, WilliamKrug, RalfWikstrom, ErikKrug, SebastianWilczek, BrigitteKrüger, DavidWilczek, PiotrKrüger, Renata L.Wilczyński, Jacek R.Kruger, Tillmann H. C.Wilder-Smith, EinarKrupinski, Elizabeth A.Wiley, LukeKruszelnicka, OlgaWilfried, SchroyensKruuse, ChristinaWilhelm, KerstinKrychtiuk, Konstantin A.Wilhelm, ThomasKrysinska, KarolinaWilk, AleksandraKrysko, KristenWilkie, KathleenKryst, ŁukaszWillen, ShainaKrysta, KrzysztofWilliams, Brent A.Krzanowski, MarcinWilliams, CarolynKrzesiński, PawełWilliams, CylieKrzykawska-Serda, MartynaWilliams, Evan J.Krzystek-Korpacka, MalgorzataWilliams, NefynKrzysztof, GorynskiWilliams, Paul F.Krzyżak, Artur TadeuszWilliams, Sian AndreaKrzyżek, PawełWilliams, TimKrzyżewski, RogerWilliamson, OwenKsienski, DoranWilliamson, Tom H.Ku, Seung-YupWillis, ErinKubesch, AliciaWilm, MatthiasKubota, MegumiWilson Pastuck, TawnyaKubota, NaotoWilson, DonnaKuboto, KazuoWilson, DuncanKuchakulla, ManishWilson, JamesKucharz, EugeneWilson, Jeffrey M.Küchle, ClaudiusWilson, Melissa LeeKuchnia, AdamWilson, R. DouglasKuchta, AgnieszkaWilson, Robin A.Kucia, KrzysztofWinbo, AnnikaKucukseymen, SelcukWinder, Gerald ScottKuczera, PiotrWindhager, ReinhardKuechle, JosephWindpessl, MartinKuhn, LouiseWiner, Ira S.Kühn, TilmanWinkels, RenateKühnel, ThomasWinkler, HeinzKulandavelu, ShathiyahWinkler, Sandra L.Kular, LaraWinkler, TobiasKulbacka, JulitaWinocur, EphraimKulczyk, TomaszWinship, AmyKulecka, MariaWinters, JeffreyKulkarni, Krishnaji R.Wise, LynKulshrestha, RichaWisman, G. Bea A.Kulu, YakupWisniewski, AdamKum, Kee-YeonWistow, Graeme J.Kumar Mishra, AjayWiszomirska, IdaKumar, AkhileshWitchel, Harry J.Kumar, GauravWitkowska-Piłaszewicz, OlgaKumar, SajeeshWitte, KerstinKumar, ShajiWittkopf, Priscilla G.Kumar, SujeetWittmann, MariaKumar, SunilWittsack, Hans-JörgKumar, VasanthWitzgall, PeterKumarasamy, SivarajanWłodarczyk, MarcinKumari, AshaWłoga, DorotaKumari, SonamWojakowski, WojciechKumari, SudhaWojcik, GrzegorzKume, KensukeWójcik, MałgorzataKumrungsee, ThanutchapornWojciuk, BartoszKuna, EwelinaWojnar, MarcinKunicki, MichałWojsyk-Banaszak, IrenaKunkle, BrianWołczyński, SławomirKuno, ToshikiWoldeamanuel, Yohannes W.Kupczyńska, KarolinaWoldeamanuel, Yohannes WoubishetKuphal, SilkeWolf, NicoleKurajoh, MasafumiWolf, PeterKurasawa, KazuhiroWolf, RisaKurata, MieWolf, StevenKurath-Koller, StefanWolff, A. P.Kure, ShokoWolffenbuttel, Bruce H. R.Kurian, SunilWolin, EdwardKuroda, YasuhiroWollebo, Hassen S.Kuroki, HiroshiWolley, MartinKuroki, LindsayWollheim, Frank A.Kurre, PeterWolter, TilmanKurumaddali, AbhinavWolyniec, WojciechKuruvilla, DeenaWon, Chang WonKurz, EduardWong, AngelKurz, JonathanWong, Ching-OnKurz, KatharinaWong, Christopher X.Kusa, JacekWongboonsin, JanewitKusejko, KatharinaWongtrakool, CherryKushibiki, ToshihiroWood, Andrew JamesKushima, MikiWood, Bradford J.Kushiyama, AkifumiWood, StephenKusumbe, AnjaliWoodbury-Smith, MarcKusztal, MariuszWoods, Thomas CooperKuwabara, MasanariWoodside, BlakeKuwata, ShingoWoopen, HannahKvist, ThomasWormser, UriKwak, Sang WonWöstmann, BerndKwok, Seung-KiWouthuyzen-Bakker, MarjanKwon, DeukwooWrenger, SabineKwon, Soon KilWrensch, FlorianKwong, DianaWrigge, HermannKyba, MichaelWright, LauraKylat, RanjitWright, Nicole C.Kyriakides, Tassos C.Wright, Patricia P.Kyriazis, George A.Wright, Rebecca J.Kytö, VilleWróblewski, KarolLa Grutta, StefaniaWrona, EwaLa Manna, GaetanoWrzesinski, JanLa Mantia, IgnazioWrześniok, DorotaLa Rocca, Renato V.Wu, ChunsenLa Starza, RobertaWu, JianhongLa Torre, GiuseppeWu, LeiLabaig-Rueda, CarlosWu, Melinda D.Labbé, AntoineWu, MingfuLabib, Peter L.Wu, MinghuaLabilloy, AnataliaWu, Monica S.Labrosse, RoxaneWu, Pei-HsunLacalzada-Almeida, JuanWu, PingshengLacatusu, Cristina MihaelaWu, RongxueLace, John W.Wu, Rongxue (Rosie)Lachapelle, KevinWu, TeddyLachkar, SamyWu, TianyingLachman, HerbertWu, ZeguangLacitignola, LucaWu, Zhongjun JonLackner, KarolineWuilleme, SorayaLacomba, María TorresWulffraat, Nico M.Lacroix, AndréWulfman, ClaudineLacy, Dana BordenWunderink, RichardLacy, JillWundsam, HelwigLadores, SigridWurm, MarkusLadyman, SharonWychowaniec, JacekLaessle, ReinholdWychowański, PiotrLaeyendecker, OliverWylegala, EdwardLaFountaine, MichaelWyndaele, MichelLafranceschina, StefanoWynne, KatieLagalwar, SaritaWyrobek, Andrew JLaganà, Antonio SimoneWystrychowski, GrzegorzLaghezza, AntonioXanthou, GeorginaLagneaux, LaurenceXi, GuifaLagrange, JeremyXiao, Shu-YuanLaguna, María PilarXiao, WenzhongLagundzin, DraganaXie, BingxianLaheij, Alexa MXie, WankunLahiji, Shayan FakhraeiXie, YibinLahner, EdithXin, YinLai, SilviaXiong, XiaoliLai, YandongXu, GuofangLai, YanhaoXu, JunwangLaine, MikaXu, WilliamLaird, Amanda M.Xu, YanLairez, OlivierXu, YuexinLajolo, CarloXue, XiangLakhan, RamYa’Qoub, LinaLakshmikuttyamma, AshakumaryYadav, DhananjayLal, SaurabhYadav, LalitLam, MaiYadavalli, TejabhiramLam, Wai-ChingYagishita, AtsuhikoLamarca, AngelaYaku, HidenoriLambert, JohnYamada, HirotsuguLambert, Scott R.Yamada, SatoruLambert, SylvieYamada, YoshiyukiLambert, ThomasYamaguchi, KoheiLamberti, NicolaYamaguchi, SatoshiLamberts, Jennifer T.Yamakado, KotaroLambregts, Merel M. C.Yamakawa, HideakiLameijer, Charlotte M.Yamamoto, HideyaLameiras, MaríaYamamoto, HironoriLamhamedi-Cherradi, Salah-EddineYamamoto, KenjiroLammens, JohanYamamoto, NorioLammer, JanYamamoto, ShuheiLamort, Anne-SophieYamanashi, HirotomoLamote, KevinYamanoi, KazuhiroLamy, Pierre-JeanYamasaki, ShoLan, James H.Yamashita, KennosukeLancaster, Cynthia LYamashita, KentaroLancee, JaapYamashita, SatoshiLand, HelenYamauchi, Melissa S. W.Landau, DanielYamazaki, SusumuLandgraf, MirjamYan, ShengminLandgren, OlaYanagisawa, SatoshiLane, TristanYang, Angela Wei HongLang, AnnemarieYang, BoLang, Cajetan ImmanuelYang, Byoung-eunLang, Mark L.Yang, Jong-ChulLang, MichaelYang, Joshua Y. C.Lang, Nikolaus WilhelmYang, Kwang IkLang, Sven ArkeYang, MihiLange, JeppeYang, Nan-PingLange, Margaret J.Yang, ShanLange, Philipp S.Yang, WeiguangLanger, FlorianYang, YangLang-Roth, RuthYang, YichiLangsjoen, PeterYang, ZhaohaiLangsteger, WernerYang-Hartwich, YangLanks, Charles W.Yanik, Elizabeth L.Lanktree, Matthew B.Yano, YoshihikoLanna, MarianoYao, Qizhi CathyLanot, AntoineYaoeda, KiyoshiLansink-Hartgring, Annemieke OudeYap, Sing-ChienLantta, TellaYarlas, AaronLanza, AlessandroYaron, Jordan R.Lanza, Gaetano AntonioYarur, Andrés J.Lanza, GiuseppeYashkin, Arseniy P.Lanza, MicheleYasmeen, AmberLanzillotta, MarcoYasuhiko, KogaLao, ChunhuanYates, Jennifer A.Laoudj-Chenivesse, DalilaYates, Nathanael J.Łapaj, ŁukaszYawn, BarbaraŁapińska, BarbaraYayan, JosefLaplante-Lévesque, ArianeYazawa, MasahikoLapole, ThomasYazdanyar, AmirfarbodLaporte, SébastienYderstræde, Knud BonnetLara, LoboYe, Young-MinLaranjeira, CarlosYeh, Chien HungLarner, AndrewYeh, HeidiLarner, Andrew J.Yeh, Kun-yunLarrañaga, PedroYeh, Te-HueiLarsen, Erik RojYessayan, Lenar TatiosLarsson, ChristelYeung, DarwinLarussa, TizianaYeung, Jacky T.Lasa, Javier J.Y-Hassan, ShmasLascano, JorgeYi, Jin WookLaselva, OnofrioYi, Joo MiLashley, Lisa E. E. L. O.Yilmaz, MusaLaskaratos, Faidon-MariosYilmaz, Sevilay TümkayaLaski, DariuszYimlamai, DeanLaskin, DanielYin, KanhuaLasko, PaulYin, LiLaskowska, MarzenaYin, WeiyanLass, RichardYinon, YoavLassalle, AmandineYip, Bon HamLassalle, Henri-PierreYokomichi, HiroshiLastra, Ricardo R.Yokoo, HirokiLászló, PajorYokoyama, TadafumiLatif, AyseYombi, Jean CyrŁątka, DariuszYoneda, MasahiroLatour, Lawrence L.Yong, TuckLatronico, NicolaYonker, LaelLatsios, GeorgeYoo, Dae HyunLau, ChunghoYoo, Jae-SungLaubli, HeinzYoo, JoonsangLaukkanen, Jari AnteroYoo, Jun-IlLAURETANI, FulvioYoon, Hye EunLauretta, RosaYoon, Hyung-InLauridsen, Kasper GlerupYoon, Kang SupLauridsen, Mette MunkYoon, UzungLauritano, DorinaYordanov, AngelLauro, DavideYoshida, HiroshiLauschke, Volker M.Yoshida, ManabuLaux, Christoph J.Yoshida, NaoyasuLavalle, CarloYoshida, TaketoshiLavazza, MatteoYoshida, ToshikoLavie, CarlYoshida, YuriLavie, LenaYoshie, MikihiroLavine, Steven J.Yoshigaki, JunkoLawson, JamesYoshihiro, TakamuraLay-Flurrie, Sarah L.Yoshikawa, YusukeLazarević, VladimirYoshikazu, GotoLazaris, AnthoulaYoshimatsu, HidehikoLázaro Pérez, CristinaYoshimoto, SeiichiLazo-Langner, AlejandroYoshimura, AtsutoshiLazzaroni, Maria GraziaYoshino, OsamuLe Barz, MélanieYoshioka, ShinichiroLe Saux, OlivierYoshiyama, TakashiLeach, KatieYoshizumi, MasanoriLeal-Blanquet, JoanYost, Christian ConLeão, TeresaYoun, Hyun JoLeavis, Helen L.Young, AllanLeavy, BreiffniYoung, BryanLeavy, OliviaYoung, Cara E. CapitenaLebenthal, YaelYoung, GraemeLeblebicǐoǧlu, BinnazYoung, MichaelLebleu, BernardYoungster, IlanLeclerc, EstelleYoungstrom, Daniel W.Lecointre, LiseYu, Hyeong WonLeconte, ClaireYu, JeremyLecureux, JonathanYu, Margaret K.Leddy, JohnYu, WenguiLedeganck, Kristien J.Yu, XijieLedesma, Juan CarlosYu, YanboLedford, Dennis K.Yu, Yong-mingLedin, TorbjörnYu, ZhipingLee, Byung-WanYuan, BaoyinLee, Chang HoonYue, John K.Lee, Chang-minYumoto, HiromichiLee, Chu-YuYumoto, TetsuyaLee, Dae HyunYun, ChawonLee, DarrenYun, Sook JungLee, David D.Yunusa, IsmaeelLee, Eun JiYurkewich, AaronLee, EunkyungYuste-Sánchez, Maria JoséLee, Hae KyungYusuf, Syed WamiqueLee, HaneulYuzhalin, Arseniy E.Lee, HeeyoungZaami, SimonaLee, Hui JaiZabad, Rana K.Lee, In-SeopZabetakis, IoannisLee, Jae-HyunZaborina, OlgaLee, JeongminZacchia, MiriamLee, JessicaZachariae, ClausLee, JeungchanZacharioudakis, Ioannis M.Lee, JieZachow, StefanLee, JongminZacks, David NLee, Joon HyopZadik, YehudaLee, JuEunZaffagnini, StefanoLee, Jun NyungZagalaz-Anula, NoeliaLee, JungwooZahnreich, SebastianLee, JunyeopZaidi, Syed Tabish R.Lee, JuyeunZajączkowska, RenataLee, Kyung-AhZakaraya, RaziaLee, Kyung-YilZakrocka, IzabelaLee, Man-JongZakrzewska-Koperska, JoannaLee, MariaZaleska-Kociecka, MartaLee, Matthew Meng YangZalewski, Bartłomiej M.Lee, Min YoungZalewski, JaroslawLee, Min-WooZalud, IvicaLee, Moo YeolZaman, KhalilLee, Pyung-BokZamani, AkramLee, Randall J.Zambrano, Byron A.Lee, Sang J.Zamora, RubenLee, Sang-MokZampieri, NicolaLee, Seo-JoonZampini, MatteoLee, Seung-GeunZampino, RosaLee, Seung-HwanZampogna, AlessandroLee, Shoo K.Zampogna, BiagioLee, Si HyungZanella, AlbertoLee, Sonny T. M.Żaneta, CiosekLee, SookyungZanetto, AlbertoLee, StellaZange, SabineLee, Tai-PingZannella, CarlaLee, Teresa M.Zannin, EmanuelaLee, VivianZannoni, Gian FrancoLee, WachtelZanotti, RenzoLee, WooCheolZanza, ChristianLee, Yong SeukZapolski, TomaszLee, Young JinZaraca, FrancescoLee, Young-kyunZaręba, KorneliaLee, ZhenghongZarone, FernandoLeem, JaechanZaucha, JanLeenen, Luke L. P. H.Zavattaro, ElisaLees, Jennifer S.Zavattini, AngeloLees, JohnZayat, RashadLefering, RolfZaza, GianluigiLeftheriotis, GeorgesZbeidy, ReineLeftwich DO, Heidi K.Zbroch, EdytaLegaz, IsabelZeebari, ZanginLéger, BertrandZegrean, MihaelaLeǵer, DamienZehnder, MatthiasLegnani, ClaudioZeidler, ReinhardLegrand, KarineZeigerer, AnjaLehmann, Lorenz H.Zeigler-Johnson, Charnita M.Lehner, MałgorzataZeiler, MatthiasLehto, MikaZeilig, GabrielLei, WeiZeinali, MinaLeibovitz, EyalZeki, SebastianLeibowitz, AkivaZeller, Alexander-N.Leibowitz, AvshalomZellers, Jennifer A.Leinung, MatthewZeman, Catherine L.Leire, UnzuéZengin, AyseLeirós-Rodríguez, RaquelZenk, JohannesLeite, Fábio R. M.Zerahn, BoLeivonen, S. K.Zerbini, GianpaoloLekoubou, AlainZervou, Maria I.Lemaire, AnthonyZeuschner, PhilipLemaire, AntoineZeyer, AlbertLembo, GiuseppeZghebi, Salwa S.Lemma, EnricoZhalniarovich, YauheniLemmer, BjörnZhand, SarehLempereur, MathieuZhang, ChiLeng, TiandongZhang, DuoLengyel, ImreZhang, HanmingLenihan, Daniel J.Zhang, HuiLennartsson, FredaZhang, LanjingLenschow, C.Zhang, LingLeon Mateos, LuisZhang, MichaelLeonard, FransiscaZhang, QianLeonardi, MathewZhang, ShuLeonardi, RosaliaZhang, TonyLeonelli, FabioZhang, WencaiLeong, Tracey LZhang, XiaotaoLeong, Yew AnnZhang, XiaowenLeonida, AlessandroZhang, XinLeopoulou, MariannaZhang, XinminLepage, CecileZhang, XiujuanLepercq, JacquesZhang, Xu-ShengLepiller, QuentinZhang, Yin HuaLeprince, JulianZhang, YixuanLe-Rademacher, Jennifer G.Zhang, Zheng GangLerma, ClaudiaZhang, ZhiguoLesauskaitė, VitaZhang, ZhiqiLeslie, MonicaZhao, Hong-BoLeszczak, JustynaZhao, LeeLeszczyński, BartoszZhao, MinLetarte, MichelleZhao, MingjunLethaus, BerndZhao, MingtaoLeube, AlexanderZhao, QianLeucker, Thorsten M.Zhao, WenxiuLeung, CharZhao, YingxinLeung, VannessaZhao, ZhanqiLeusen, JeanetteZhao, ZhenLev, ShaulZheng, WenxinLevesque, EricZhornitsky, SimonLevesque, MarioZhou, ChiLevin, Lawrence ScottZhou, JerryLevinson, Cheri A.Zhou, JianLevis, MarioZhou, LingliLevitz, Lewis M.Zhou, MingchuanLevkovich, InbarZhou, XiaofengLevy, JeremyZhou, XiaohaiLevy, JerroldZhou, YanjiaoLewald, HeidrunZhou, ZhichaoLewalle, PhilippeZhou, ZhijunLewis, ChandaniZhu, ChengjingLewis, Frances M.Zhu, DongshanLewis, GwynZhu, JunjieLewis, James H.Zhu, LinLewis, Janina P.Zhurakivska, KhrystynaLewko, AgnieszkaZięba, MariuszLi, ChunyingZieger, MarinaLI, Hai-changZiegler, Kenneth R.Li, HaoboZielinska, MagdalenaLi, HuaZielińska, Monika A.Li, HuinanZiemssen, FockeLi, HuiquanZieniewicz, KrzysztofLi, LiZiff, RobertLi, Mei-LingZilberman, UriLi, SongZimmer, SebastianLi, TieshiZimmerli, WernerLi, TimmyZimmerman, KurtLi, Tin-chiuZimmermann, MarkusLi, WeiZimran, AriLi, XiangZińczuk, JustynaLi, XiaotaoZiogas, IoannisLi, XinboZipfel, SvanteLi, YanZissel, GernotLi, YikeZitta, SabineLi, YupengZittermann, ArminLi, ZiruZivotofsky, AriLiamis, GeorgeZlotta, Alexandre R.Liampas, IoannisZmarzły, NikolaLiao, XudongZmijewski, MichalLiapikou, AdamanthiaZmysłowska, AgnieszkaLiarski, VladimirZopf, EvaLiauw, WinstonZorzi, ManuelLibonati, LauraZou, ChunbinLicari, LeoZouhal, HassaneLichte, PhilippZrinzo, Ludvic U.Lichtenauer, MichaelZubair, HaseebLichtenstein, EricZubizarreta-Macho, ÁlvaroLichtenstein, Mia BeckZucchiatti, IlariaLicini, CaterinaZuhl, MicahLidbury, BrettZuk, MalgorzataLiddell, Robert P.Zur, DinahLie Fong, SharonZurawa-Janicka, DorotaLieberman, ScottŻurawska-Płaksej, EwaLiebman, HowardZusterzeel, PetraLiebschner, Michael A. K.Zweifel, DanielLiefferinckx, ClaireZweiker, David

